# Acyclic nucleoside phosphonates with adenine nucleobase inhibit *Trypanosoma brucei* adenine phosphoribosyltransferase in vitro

**DOI:** 10.1038/s41598-021-91747-6

**Published:** 2021-06-25

**Authors:** Eva Doleželová, Tomáš Klejch, Petr Špaček, Martina Slapničková, Luke Guddat, Dana Hocková, Alena Zíková

**Affiliations:** 1 Institute of Parasitology, Biology Centre, Czech Academy of Sciences, Branišovská 31, 37005 České Budějovice, Czech Republic; 2 The Institute of Organic Chemistry and Biochemistry of the Czech Academy of Sciences, Flemingovo nám. 2, 166 10 Prague 6, Czech Republic; 3 The School of Chemistry and Molecular Biosciences, The University of Queensland, Brisbane, QLD 4072 Australia; 4 Faculty of Science, University of South Bohemia, Branišovská 31, 37005 České Budějovice, Czech Republic

**Keywords:** Biosynthesis, Enzymes, Nucleic acids, Small molecules, Target identification, Parasite biology, Pathogens

## Abstract

All medically important unicellular protozoans cannot synthesize purines de novo and they entirely rely on the purine salvage pathway (PSP) for their nucleotide generation. Therefore, purine derivatives have been considered as a promising source of anti-parasitic compounds since they can act as inhibitors of the PSP enzymes or as toxic products upon their activation inside of the cell. Here, we characterized a *Trypanosoma brucei* enzyme involved in the salvage of adenine, the adenine phosphoribosyl transferase (APRT). We showed that its two isoforms (APRT1 and APRT2) localize partly in the cytosol and partly in the glycosomes of the bloodstream form (BSF) of the parasite. RNAi silencing of both APRT enzymes showed no major effect on the growth of BSF parasites unless grown in artificial medium with adenine as sole purine source. To add into the portfolio of inhibitors for various PSP enzymes, we designed three types of acyclic nucleotide analogs as potential APRT inhibitors. Out of fifteen inhibitors, four compounds inhibited the activity of the recombinant APRT1 with Ki in single µM values. The ANP phosphoramidate membrane-permeable prodrugs showed pronounced anti-trypanosomal activity in a cell-based assay, despite the fact that APRT enzymes are dispensable for *T. brucei* growth in vitro. While this suggests that the tested ANP prodrugs exert their toxicity by other means in *T. brucei*, the newly designed inhibitors can be further improved and explored to identify their actual target(s).

## Introduction

Trypanosomatid parasites (e.g. *Trypanosoma* spp., *Leishmania* spp.) are incapable of purine synthesis de novo and acquire purine derivates from their environment^1^. As a consequence of the complete dependency on an external purine source, these parasites have developed a complex purine salvage pathway (PSP) that enables them to uptake, metabolize and incorporate any naturally occurring purine nucleobases and nucleosides into their nucleotide pools. Therefore, purine derivatives have been considered as a promising source of anti-parasitic compounds. They can inhibit the PSP enzymes or they can become toxic (e.g. cordycepin, tubercidin) when activated by these enzymes^2–5^. Several PSP enzymes (i.e. GMP synthase, hypoxanthine–guanine-(xanthine) phosphoribosyltransferases HG(X)PRT) were recently validated experimentally as promising therapeutic targets^6,7^.

Acyclic nucleoside phosphonates (ANPs) represent a group of compounds whose biological activity is based on their structural resemblance to the natural nucleotides^8,9^. Their flexibility enables them to adopt a conformation suitable for the interaction with the active site of the nucleotide binding enzymes. Structurally, this type of nucleotide analog is characterized by a heterocyclic base linked to a phosphonate group by various acyclic chains mimicking sugar moiety. These nucleotide analogs are excellent templates for the drug design because of the absence of the labile glycosidic bond and the stability of the phosphonate moiety compared with the phosphate ester bond that can be easily enzymatically or chemically hydrolyzed^10^. The presence of the phosphonate group in the ANPs is responsible for their highly polar character and deprotonation at physiological pH. The prodrug approach has been developed to mask the charge of the phosphonate group and to facilitate the transport across the cell membranes independently from the nucleoside transporters, therefore, improving their pharmacological properties^11,12^. The similarity to the natural nucleotides predestines ANPs as suitable inhibitors of the PSP enzymes^13–16^ and as tools to study intertwined PSP with the potential of becoming promising chemotherapeutics against diseases caused by parasites incapable of the purine synthesis de novo.

**Figure 1:**
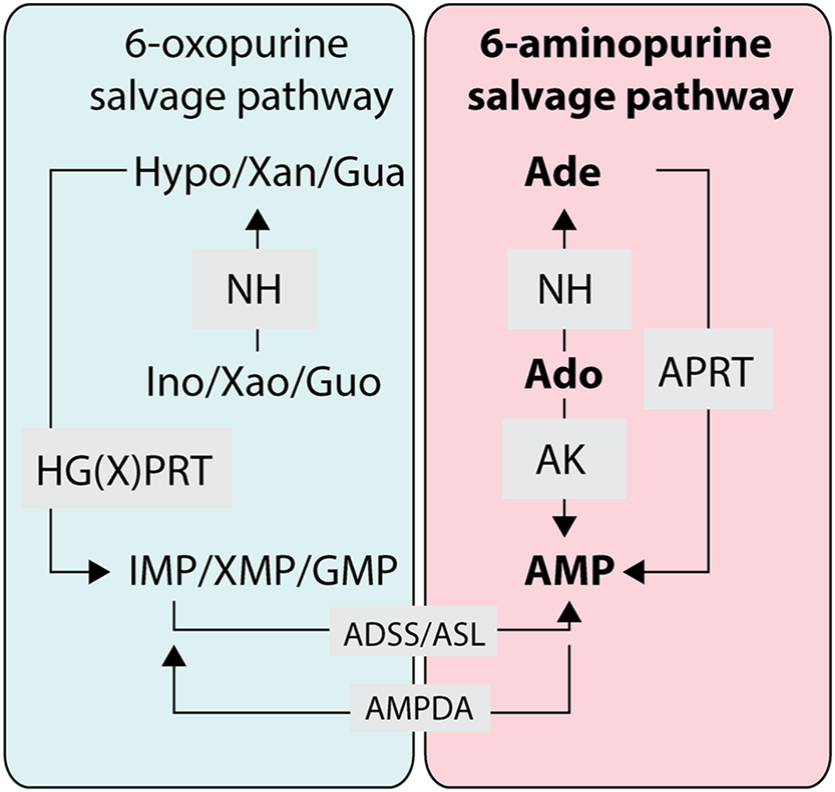
Simplified scheme of purine salvage pathway in *Trypanosoma brucei.* *APRT* adenine phosphoribosyltransferase, *HG(X)PRT* hypoxanthine–guanine (xanthine) phosphoribosyltransferase, *AK* adenosine kinase, *ADSS* adenylosuccinate synthetase, *ASL* adenylosuccinate lyase, *AMPDA* AMP deaminase, *NH* nucleoside hydrolase, *Ado* adenosine, *Ade* adenine, *Ino* inosine, *Hypo* hypoxanthine, *Guo* guanosine, *Gua* guanine, *Xao* xanthosine, *Xan* xanthine. 6-oxopurine metabolism is tinged with light blue, while the 6-aminopurine metabolism is tinged with light pink. The figure was drawn using Adobe Illustrator CS6 (www.adobe.com).

In our previous work, we determined the crystal structures of two *T. brucei* PSP enzymes, 6-oxopurine PRTases, in the complex with several ANPs, and showed that the prodrugs of the selected inhibitors possess strong anti-trypanosomal activity in the cell-based assay^6,17,18^. Inspired by these results, here we focused on the 6-aminopurine salvage route, which is linked to the 6-oxopurine pathway via an AMP deaminase that converts AMP to IMP (Fig. 1).

In *T. brucei*, hypoxanthine is a preferential source of 6-oxopurines, while adenosine feeds the aminopurine-based salvage pathway. Upon its entry to the cell, the adenosine can be converted to the adenosine monophosphate (AMP) by an adenosine kinase (AK)^19,20^ or it can be cleaved to the adenine by a nucleoside hydrolase (NH)^21^ and then be converted to the AMP by an adenine phosphoribosyltransferase (APRT)^22^. The APRT catalyzes the formation of the AMP via reaction of the adenine and the 5-phospho-α-d-ribosyl-1-pyrophosphate in the presence of a divalent metal ion (in vivo magnesium), an essential co-factor of this enzymatic reaction. The APRT is encoded by two genes in the *T. brucei* genome (APRT1 and APRT2), which differ significantly at the nucleotide level sharing only 23% identity at the amino acid level. In the *T. brucei* insect procyclic form (PCF), the APRT1 is localized to the cytosol while the APRT2 is a glycosomal enzyme. Their simultaneous deletion did not affect the growth of the PCF cells in vitro^22^.

To get more insights into the physiological role of these enzymes in the bloodstream form (BSF) of the parasite, we assessed the cellular localization of APRT1 and APRT2 enzymes and their importance for the growth of BSF *T. brucei* cells. We also evaluated the enzymatic properties of the recombinant *T. brucei* APRT1 and tested several types of adenine-bearing ANPs as potential inhibitors of the APRT1 activity in vitro.

## Results and discussion

### In *T. brucei* BSF parasites, the APRT1 is localized in the cytosol and the APRT2 shows a partial distribution between the cytosolic compartment and glycosomes

**Figure 2:**
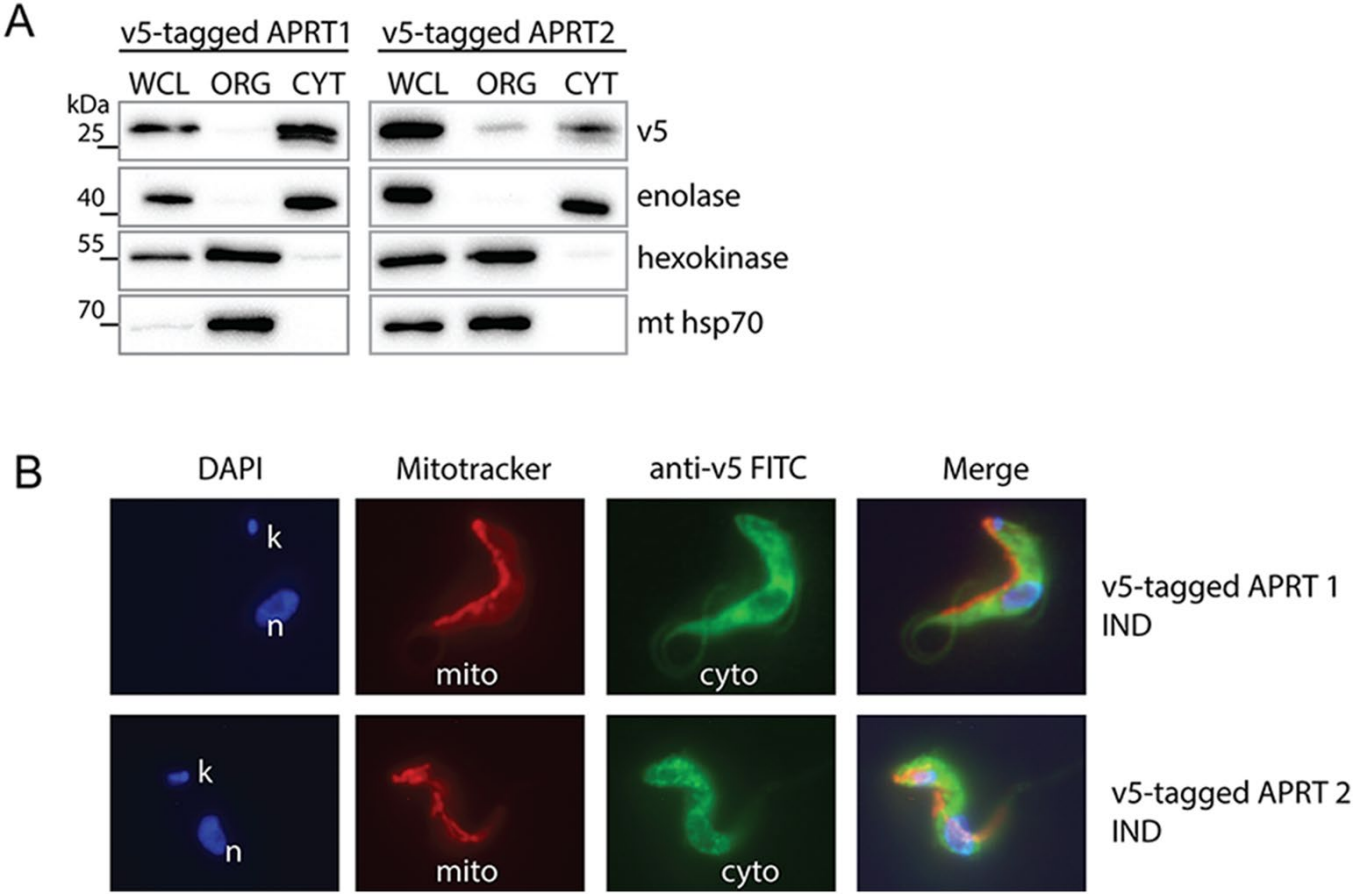
Subcellular localization of APRT1 and 2 in the BSF *T. brucei* cells. (**A**) Western blot analysis of the BSF cells overexpressing V5-tagged APRT1 and 2 which were treated with digitonin to obtain cytosolic and organellar fractions. The purified fractions were analysed with the following antibodies: anti-V5, anti-enolase (cytosol), anti-hexokinase (organellar fraction, glycosomes), and anti-mt hsp70 (organellar fraction, mitochondria). The protein marker is indicated on the left. (**B**) Immunofluorescence microscopy of the tetracycline induced (IND) V5-tagged APRT1 and 2. The tagged proteins were visualized using a monoclonal V5-antibody and an anti-mouse secondary antibody conjugated with fluorescein isothiocyanate (FITC). The MitoTracker Red was used to visualize mitochondria, while the DAPI was used to stain the DNA content [nucleus (n) and kinetoplast (k)] of the cell. *WCL* whole cell lysate, *ORG* organellar fraction, *CYT* cytosolic fraction.

In PCF *T. brucei* cells, the C-terminally tagged APRT1 and APRT2 were localized to the cytosol and glycosomes, respectively^22^. In order to decipher the subcellular localization of these two enzymes in the BSF cells, the genes were N-terminally tagged with a V5 tag not to interfere with the C-terminally localized glycosomal signal of the APRT2. Their ectopic expression was triggered by adding tetracycline into the medium. Digitonin was used to dissolve the plasma membrane allowing to enrich for intact organelles in the pelleted fraction. Western blot analysis using antibodies recognizing cytosolic enolase, glycosomal hexokinase and mitochondrial hsp70 protein revealed the purity of the cytosolic and organellar fractions (Fig. 2A). The APRT1 enzyme was found in the cytosolic fraction and this localization was further confirmed by direct immunofluorescence of the V5-tagged APRT1 (Fig. 2B), although this protein was detected in the glycosomal fraction by proteomics suggesting possible dual localization^23^. The BSF APRT2 was localized to both compartments, glycosomes as well as cytosol (Fig. 2A,B), similarly to the reported distribution of the PCF APRT2 by TrypTag.org database^24^. Our data are consistent with a partial localization of purine metabolism enzymes in glycosomes and cytosol as it is also shown for 6-oxopurine salvage pathway^6,25^.

### The APRT1 forms homodimers in vivo and in vitro

**Figure 3:**
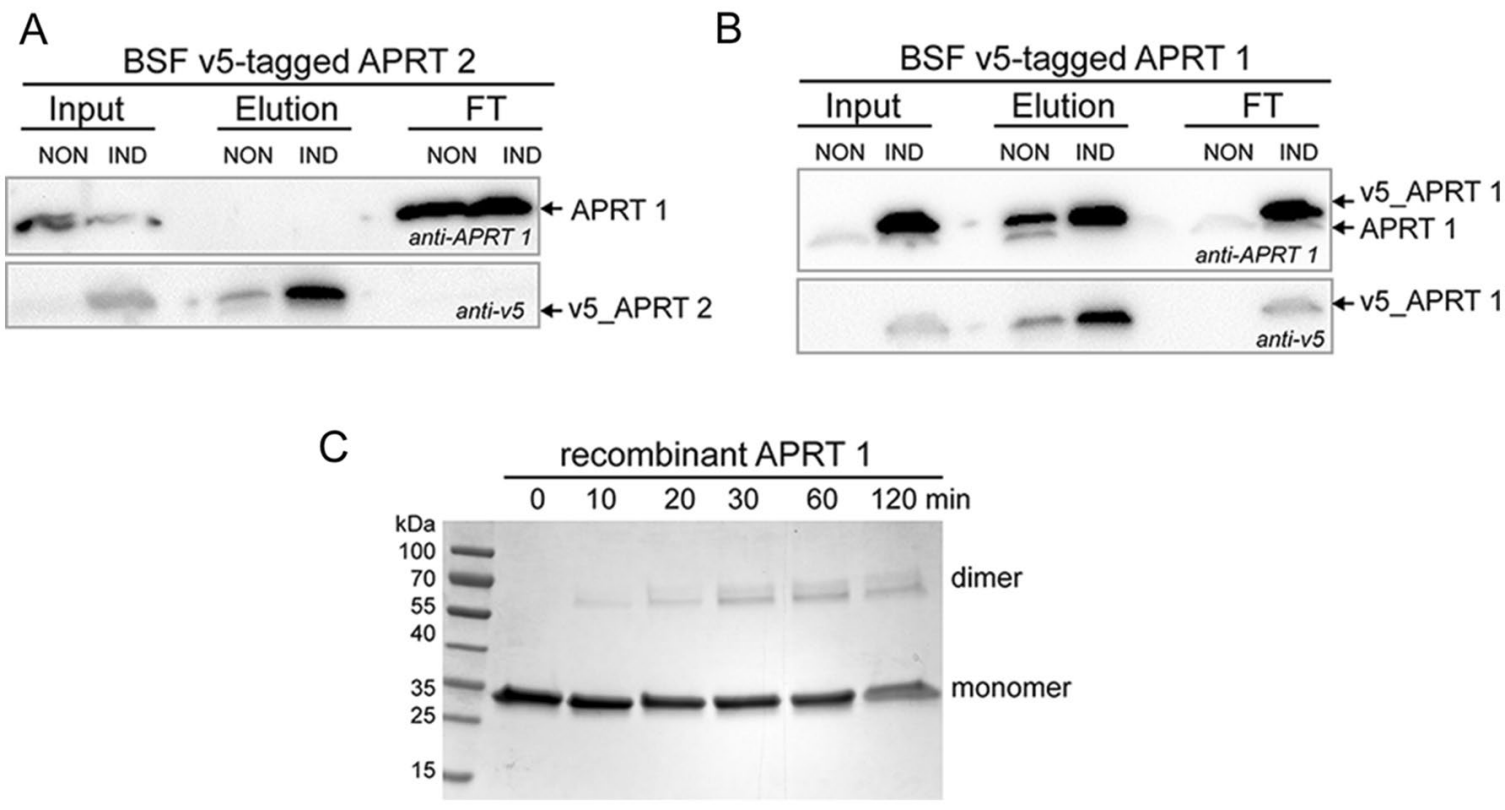
APRT1 and APRT2 do not form heterodimers, but APRT1 homodimerizes. Immunoprecipitation of the V5-tagged APRT2 (**A**) and APRT1 (**B**) from the noninduced and tetracycline induced cells followed by the immunoblotting of the elution and flow through (FT) fractions using anti-V5 and anti-APRT1 antibodies. (**C**) The recombinant APRT1 was treated with a crosslinker DMS for 0, 10, 20, 30, 60 and 120 min. The APRT1 homodimers were identified based on their sizes. The protein marker is indicated on the left.

Since the APRT enzyme functions as dimers, we wondered if the cytosolic APRT1 can form a heterodimer with the APRT2 that is partially localized to the BSF cytosol. To establish the hetero- or homodimeric properties of APRT1 and APRT2 in vivo, we employed the BSF *T. brucei* cells overexpressing V5-tagged APRT1 and APRT2. Figure 3A shows that the immunoprecipitation of the V5-tagged APRT2 recovered the tagged APRT2, but no APRT1 was detected in the eluate, suggesting that these two proteins do not interact in vivo. The homodimeric properties of the APRT1 were shown by immunoprecipitation of the V5-tagged APRT1, which recovered the tagged and endogenous APRT1 (Fig. 3B), as well as by a cross-linking experiment using dimethyl suberimidate (DMS). Figure 3C shows the cross-linked recombinant APRT1 protein forming homodimer.

### The RNAi silencing of the APRT1 and the double-silencing of APRT1/2 has no major effect on the growth of the BSF cells but leads to the growth defect in the purine restricted medium

**Figure 4:**
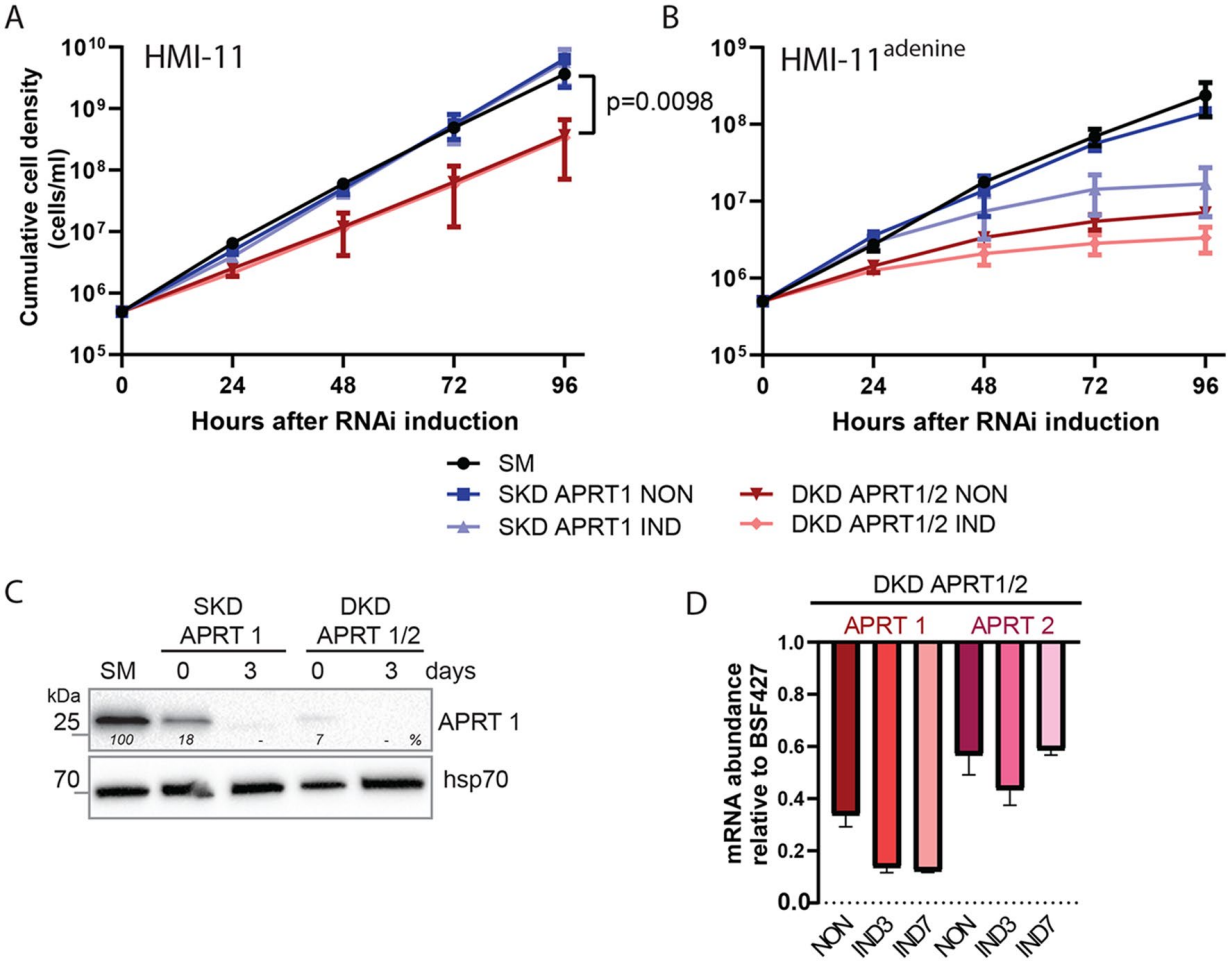
Effects of RNAi silencing of APRT1 and 2 on *T. brucei* BSF growth. (**A**) Growth curves for the parental cell line (single marker, SM), noninduced (NON) and RNAi induced (IND) cells in which the expression of only APRT1 (single knock-down, SKD) or both APRT1 and 2 (double knock-down, DKD) is silenced. The cells were cultured in the HMI-11 medium and the cumulative cell number was calculated from the cell densities adjusted by the dilution factor each day. (means ± s.d., n = 3, Student’s unpaired test). (**B**) The RNAi and SM cell lines were cultured in HMI-11^adenine^ medium and the cumulative cell number was calculated from the cell densities adjusted by the dilution factor each day. (means ± s.d., n = 3). (**C**) The steady-state levels of the APRT1 in the parental single-marker (SM) cell line, RNAi noninduced cells (0 days) and cells induced for 3 days were determined using specific anti-APRT1 antibody by the immunoblotting. The densitometric analysis was performed using the ImageLab 4.1 software with the values determined for the mitochondrial hsp70 as a loading control. (**D**) RT-qPCR analysis of the APRT1 and APRT2 transcript levels in the noninduced DKD APRT1/2 cell line (NON) and cells induced for 3 (IND3) and 7 (IND7) days of the tetracycline induction compared to the parental cells. The relative changes in the transcript abundance are plotted on a linear scale. β-tubulin and rRNA 18S transcript levels were used as internal controls.

The activity of APRT enzymes was shown to be dispensable for the PCF *T. brucei* cells grown in vitro^22^. To investigate if the same is applicable for the *T. brucei* BSF cells, we generated two RNAi cell lines: a single knock-down (SKD) of the APRT1 and a double knock-down (DKD) of APRT1 and 2. The two proteins do not share any similarity at the level of genomic DNA, ensuring the specificity of the RNAi approach. Upon the RNAi induction in HMI-11 medium, no effect on the growth was observed for the SKD APRT1 while a DKD APRT1/2 cells displayed minor, but statistically significant growth phenotype (Fig. 4A). A Western blot using the anti-APRT1 antibody verified a silenced expression of the APRT1 in both cell lines. We noted a significant reduction of the APRT1 levels in the noninduced cells suggesting a leaky expression of the dsRNA that induces RNAi silencing in the absence of tetracycline. Upon the addition of tetracycline, APRT1 expression was fully silenced at day 3 (Fig. 4C). The leaky expression was more profound in the DKD APRT1/2 cell line with only 7% of the APRT1 enzyme expressed. This observed phenomena was confirmed by a qPCR analysis showing a reduction of APRT1 in the noninduced cells when compared to the APRT1 expression levels in BSF427 cells. The qPCR analysis also verified the reduction of the APRT2 transcript in the DKD APRT1/2 cell line (Fig. 4D). The lack of a major growth phenotype upon silencing of APRT1 and 2 suggests that when the medium contains hypoxanthine, adenosine and other purine derivates, the BSF cells can fulfil their purine requirements by the interconnected 6-oxo and 6-aminopurine pathways (Fig. 1).

Since the HMI-11 medium contains 1 mM hypoxanthine as well as small amounts of adenosine coming from the fetal bovine serum^26^, we tested our generated RNAi cell lines in the home-prepared medium that contains only adenine as the purine source. The medium was supplemented with dialyzed FBS serum to remove any traces of the serum-derived nucleosides (HMI-11^adenine^). The RNAi and the parental (single marker, SM) cell lines were adapted to these new conditions for at least 2 weeks. Upon the RNAi induction, we detected a strong phenotype for the SKD APRT1 RNAi-induced cells suggesting that the APRT2 enzyme is not able to fully compensate for the loss of APRT1. Even stronger growth effect was detected for the noninduced DKD APRT1/2 cells with a low APRT1 expression and for the RNAi induced cells (Fig. 4B) implying an additive negative effect on trypanosoma growth when expression of the APRT2 is silenced. Our results show that there are no other enzymes that can convert adenine to AMP at least with the sufficient capacity and efficacy to allow growth on adenine as a single purine source. The HMI-11^adenine^ medium contained adenine only at the 50 µM concentration because this purine is toxic to the BSF cells at elevated (millimolar) concentrations. The adenine toxicity is explained by its inhibitory effect on a methylthioadenosine phosphorylase, an enzyme that mediates protection against toxic levels of deoxyadenosine^27,28^.

### Synthesis of three types of acyclic nucleotide analogues and their prodrugs

**Scheme 1:**
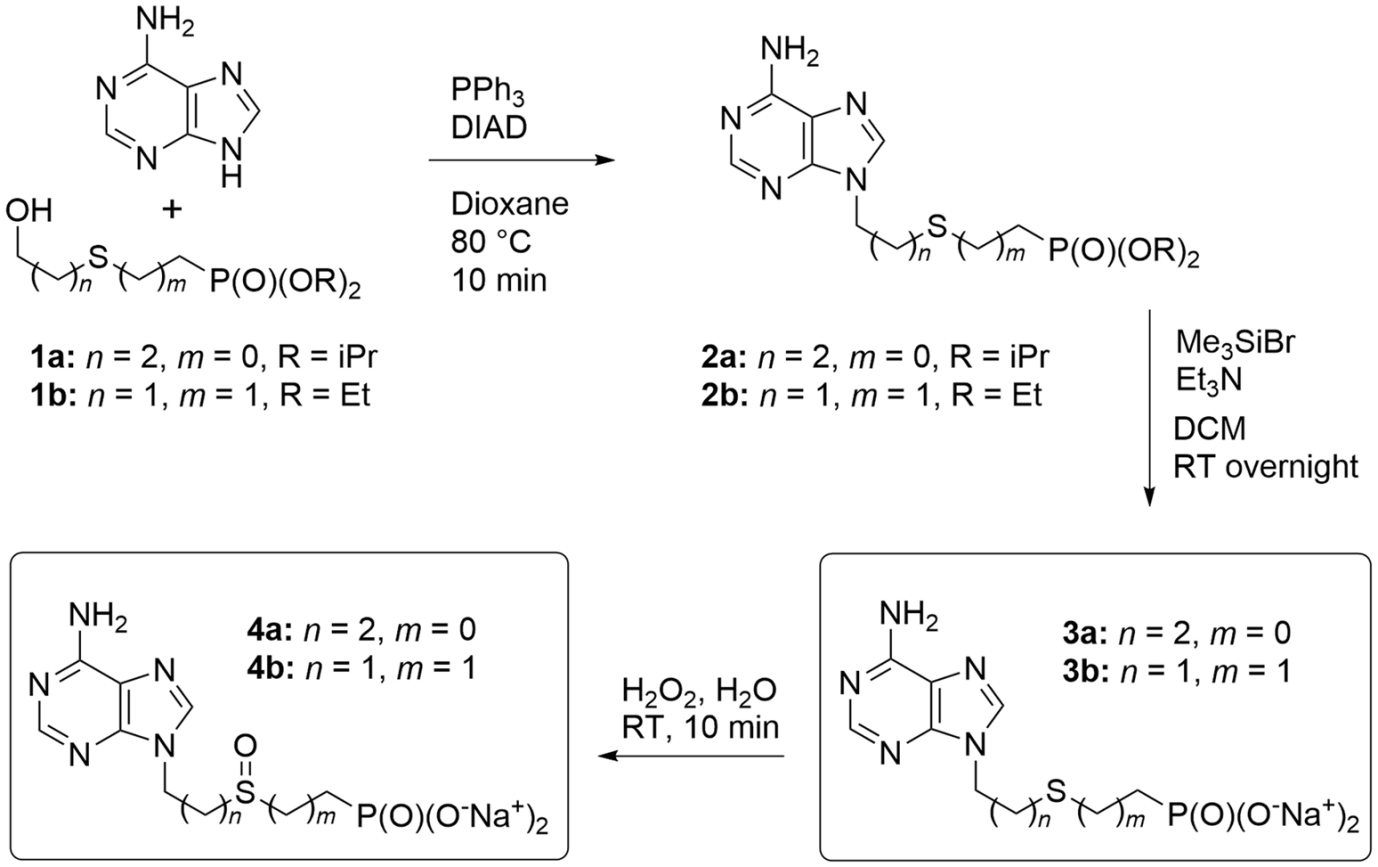
Synthesis of thia-ANPs. The scheme was drawn using ChemDraw 18.2 (PerkinElmer).

**Scheme 2:**
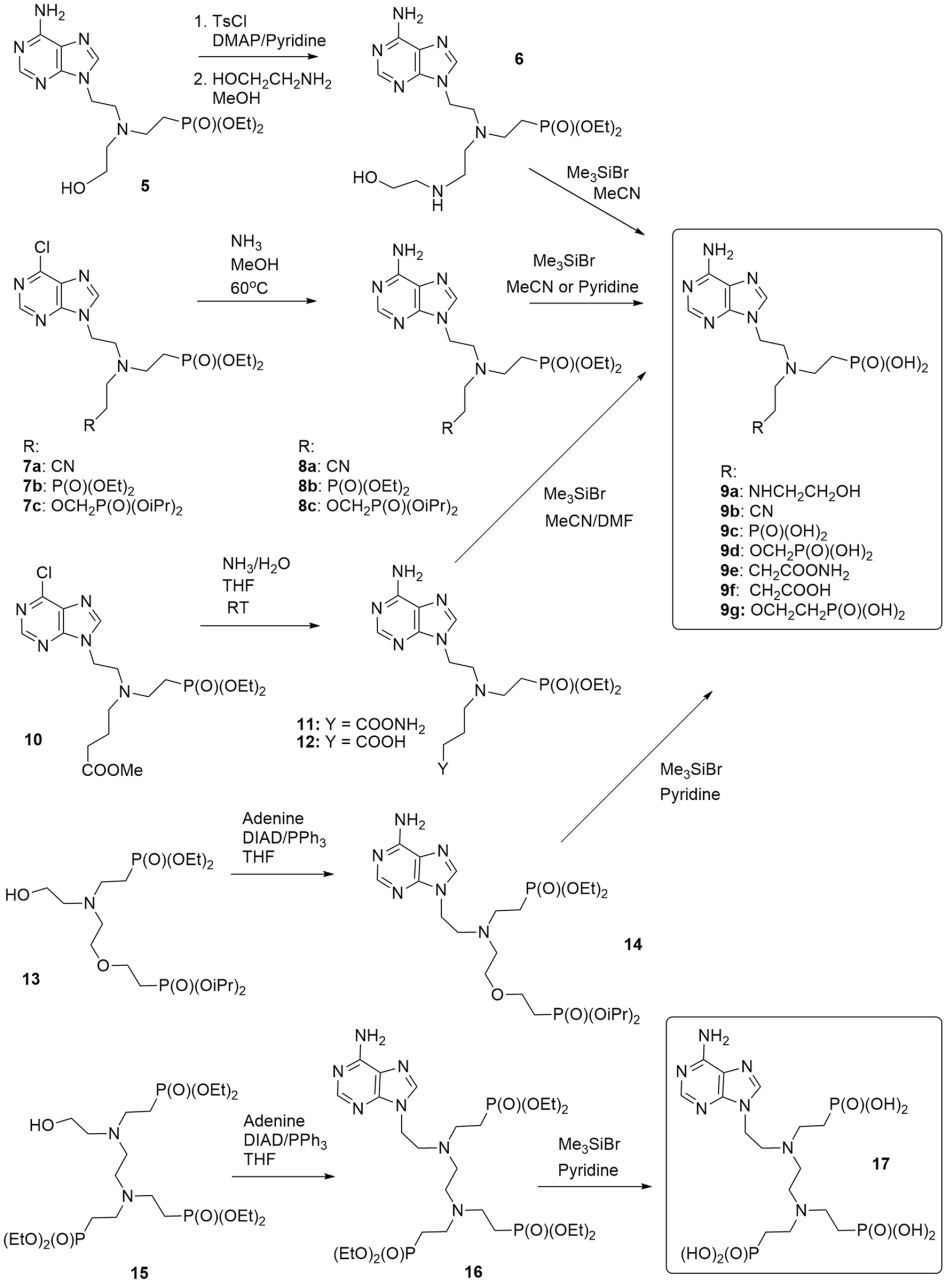
Synthesis of aza-ANPs. The scheme was drawn using ChemDraw 18.2 (PerkinElmer).

**Scheme 3:**
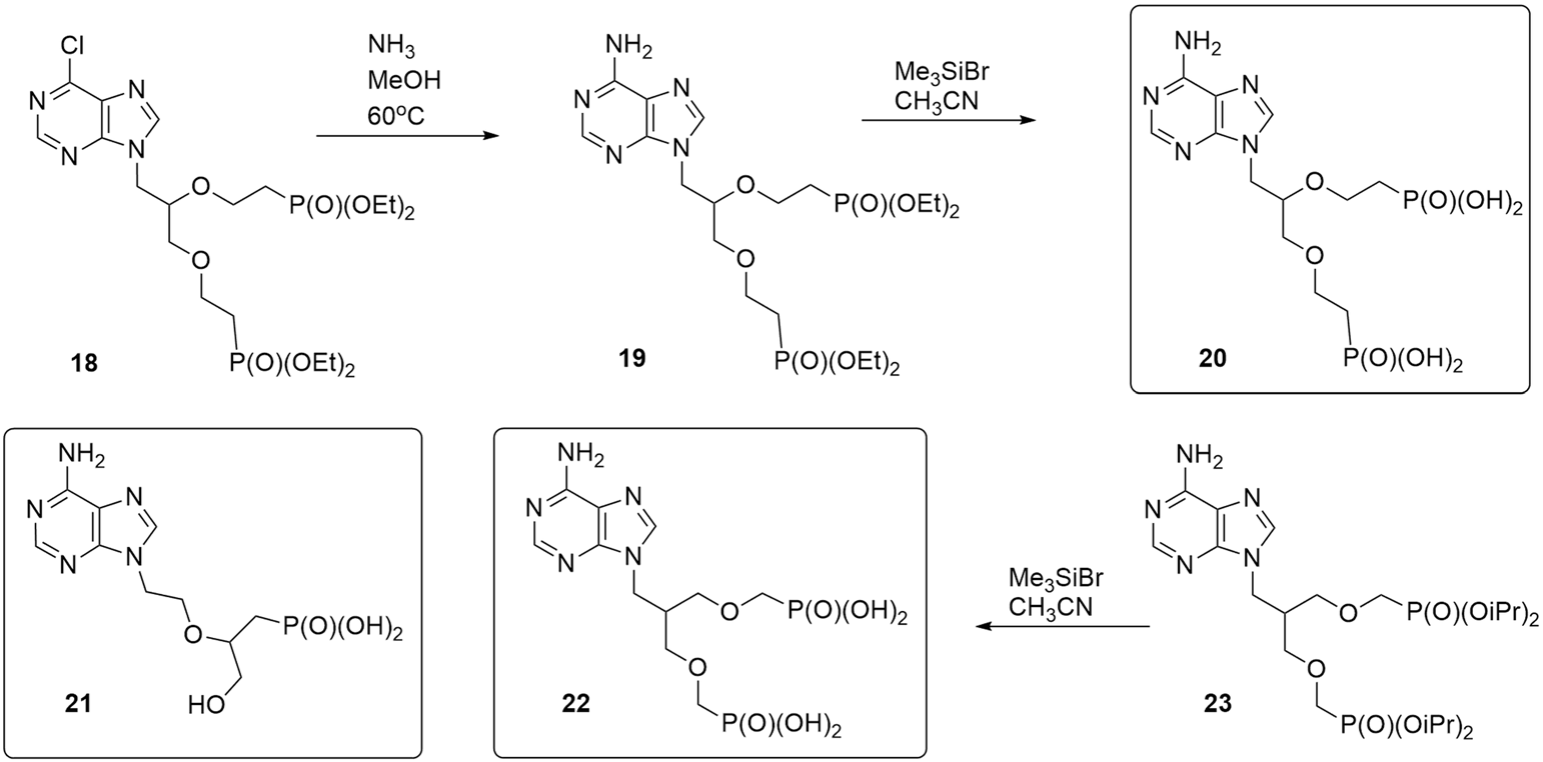
Structures of published^35,36^ and synthesis of new C-branched ANPs. The scheme was drawn using ChemDraw 18.2 (PerkinElmer).

Although the APRT enzymes are dispensable for the *T. brucei* BSF cells (this work and www.tritrypdb.org), they might become necessary when other branches of the PSP pathway are impaired. To add into the portfolio of inhibitors for various PSP enzymes, we designed three types of acyclic nucleotide analogs as potential APRT inhibitors: (a) ANPs with a sulfur-containing linker connecting adenine and the phosphonate group (thia-ANPs, Scheme 1); (b) ANPs with a nitrogen atom as a branching “hub” in the acyclic moiety (aza-ANPs, Scheme 2) and (c) ANPs using a carbon atom for the side chain attachment to the main linker (Scheme 3). Based on our previous experience with the inhibitors of HG(X)PRTs, the number of atoms between the base and phosphonate group in the linker is optimally five, while the position and nature of the heteroatom(s) in the acyclic moiety influence the flexibility and conformation of the chain(s)^29^. The side chains bearing functional groups enable further interactions in the binding site of the enzyme^14,30^.

The thia-linked nucleotide analogs (thia-ANPs, Scheme 1) were prepared analogously to a known procedure^31^. Mitsunobu reaction of adenine with alcohols **1a** and **1b** in hot dioxane afforded compounds **2a** and **2b**, respectively. The phosphonate esters were cleaved with the bromotrimethylsilane in dichloromethane to provide free phosphonic acids **3a** and **3b**, which were either transformed into the sodium salts for better solubility in the enzyme assays or directly oxidized with hydrogen peroxide to the corresponding sulfoxides **4a** and **4b** and subsequently converted into their respective sodium salts.

Inhibitors with a nitrogen atom as a branching “hub” in the acyclic moiety (aza-ANPs, Scheme 2) were prepared via three main pathways. The first method used a modification of the side chain of preformed phosphonate **5**^32^, followed by the cleavage of ester groups to form phosphonic acid **9a**. The second procedure was based on the ammonolysis of 6-chloropurine derivatives **7a–c**^30,33^ that were converted to the corresponding adenine derivatives **8a–c**. To obtain target ANPs **9a–c**, the ester groups were cleaved by Me_3_SiBr followed by hydrolysis. During the 6-Cl ammonolysis of the derivative **10**^33^, a mixture of two products, amide **11** and carboxylic acid **12**, was formed and was used without separation in the further step—phosphonate ester cleavage.

The resulting phosphonic acids **9e** and **9f** were then separated by preparative HPLC. The third approach used alcohols **13** and **15** that we have published previously^30,34^ and introduced them to N^9^-position of adenine by Mitsunobu reaction. Both target compounds, the bisphosphonic acid **9g** and trisphonic acid **17** were then obtained again after the cleavage of the ester groups of **14** and **16** using Me_3_SiBr in pyridine followed by hydrolysis.

To complement the portfolio of potential inhibitors, two previously published compounds, phosphonic acid **21**^35^ and bisphosphonic acid **22**^36^, were selected as representatives of ANPs with the carbon atom used for the side chain attachment to the main linker (Scheme 3). In addition, the bisphosphonic acid **20** was synthesized starting from a 6-chloropurine derivative **18**^37^ using an ammonolysis to form an adenine derivative **19** followed by the ester cleavage.

**Scheme 4:**
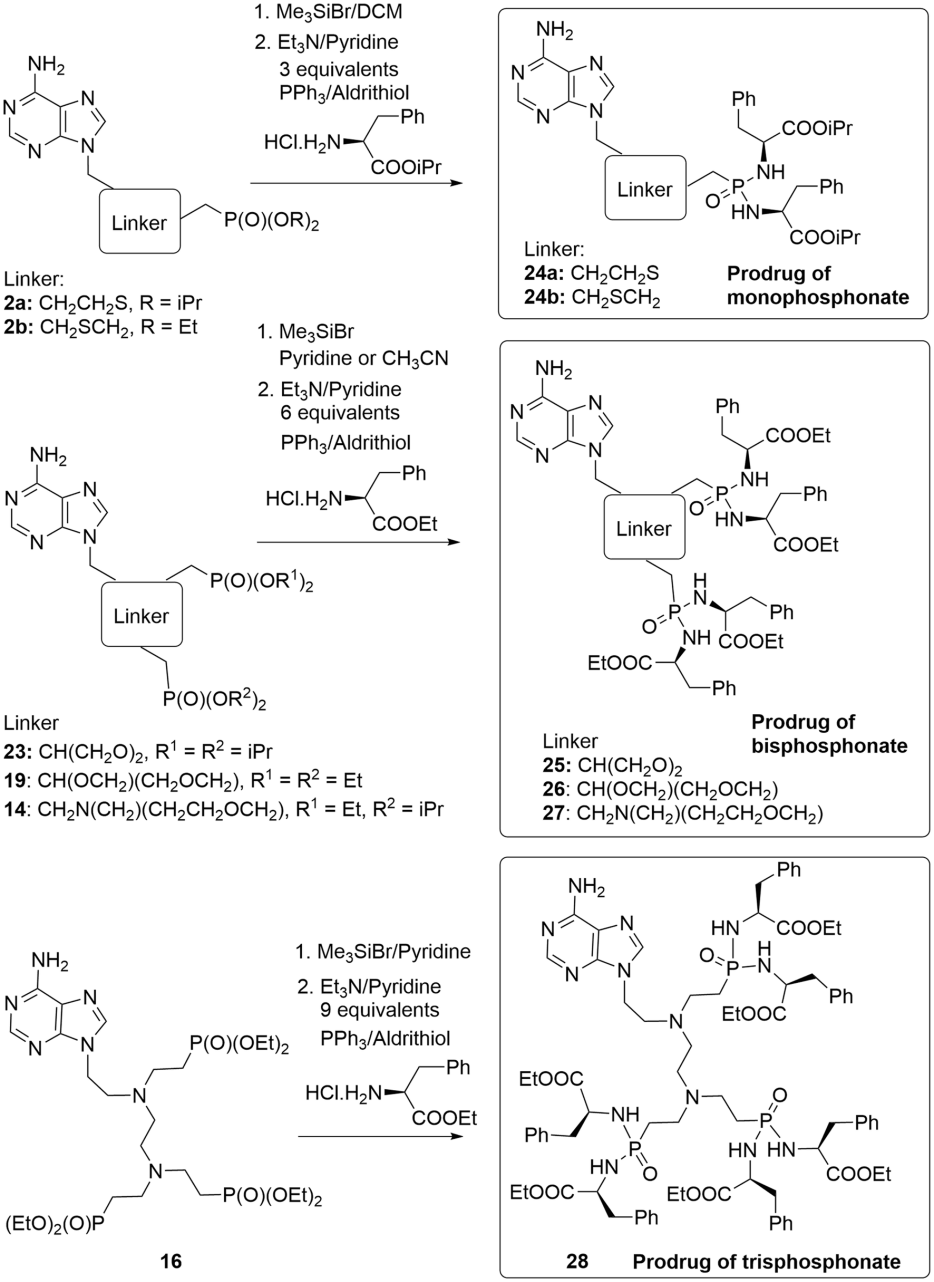
Synthesis of selected ANPs prodrugs. The scheme was drawn using ChemDraw 18.2 (PerkinElmer).

In agreement with the literature^11,12^, we have observed that suitable prodrugs of the ANP-based inhibitors can exhibit a significantly improved anti-parasitic activity in cell-based assays compared to the parent compounds^14,30,34,38^. While the penetration of free phosphonic acids to the cells is very limited, labile phosphoramidate prodrugs have previously been effective in *T. brucei* cell culture assays^6^. It appears that they are able to cross the cellular membranes efficiently and P–N bond is then cleaved inside the parasite, analogically as in the human cells^39^. They are reasonably stable and their low cytotoxicity towards mammalian cells makes them prime candidates for the development of chemotherapeutics against specific parasites. Here we prepared the bisphosphoramidate prodrugs **24a** and **24b**, tetraphosphoramidate prodrugs **25–27** and hexaphosphoramidate prodrug **28** from the corresponding esters (**2a, 2b**, **23**, **19**, **14** and **16**) of the selected ANP-based inhibitors of *T. brucei* APRTs (**3a, 3b**, **9g**, **17**, **20** and **22**). A well-established method^38^ was applied using transformation to silyl esters in the first step followed by reaction with an ester of (l)-phenylalanine in the presence of 2,2′-dipyridyl disulfide (Aldrithiol^®^) and triphenylphosphine (Scheme 4).

### The synthesized ANPs inhibit APRT1 in vitro

The ANPs studies have shown a high specificity of their biological effect with respect to the type of purine nucleobase. Previously, we observed that ANPs bearing guanine or hypoxanthine as a nucleobase inhibit the activity of the *T. brucei* 6-oxopurine PRTs^6^. To test if the newly synthesized ANPs with an adenine as a nucleobase can inhibit the activity of the APRT1 and APRT2, both enzymes were overexpressed in *E. coli*. The APRT1 was purified as an active recombinant enzyme from the soluble fraction. On the contrary, the recombinant APRT2 was found insoluble, and therefore was purified in the presence of detergent. Unfortunately, the dialyzed APRT2 enzyme did not exert any enzymatic suggesting that the remaining detergent in the APRT2 sample interfered with the enzyme’s activity. Thus, the synthesized ANP-based inhibitors were tested only as inhibitors of the recombinant APRT1. First, we monitored the APRT1 enzyme activity by a continuous spectrophotometric assay measuring the conversion of adenine to AMP at 256 nm. From the steady-state analyses we determined K_m_ and K_cat_ values for adenine (K_m_ = 8.7 ± 1.9 µM and K_cat_ = 0.82 s^−1^, respectively) and phosphoribosyl pyrophosphate (*P*Rib-*P*) (K_m_ = 162 ± 21.6 µM and K_cat_ = 1.8 s^−1^). Compared to the *Leishmania donovani* APRT1 K_m_ (2.3 ± 1.1 µM) and K_cat_ (17.9 s^−1^) for adenine and K_m_ (25.1 ± 5.9 µM) for *P*Rib-*PP*^40^, the *T. brucei* enzyme displays decreased catalytic efficiency and higher K_m_ values for adenine and *P*Rib-*PP*.

The activity of the recombinant APRT1 enzyme was then tested in the presence of synthesized ANPs. Out of the fifteen ANPs tested, seven compounds inhibited APRT1 with *K*_i_ values ranging from 3.07 μM ± 0.248 (compound **9g**) to 27.7 ± 4.46 μM, the remaining nucleotide analogs were inactive at the concentration of 30 μM (Table 1). A common structural feature of all tested ANPs is a five-atom linker connecting adenine and the phosphonate moiety, mimicking the 5-phosphate group of the natural nucleotide. All the ANPs contain one heteroatom (S, N or O) in this main linker. The heteroatom increases the flexibility of the chain and modulates its position in the active site (compare activity of **3a** and **3b**). While sulfur enables a change in the geometry via oxidation^31^, but results in the loss of the inhibitory activity (compare **3a** and **4a**), nitrogen makes a facile attachment of the side chain possible. Moreover, these aza-ANPs are prochiral inhibitors (**9a–9g** and **17)**, since nitrogen atom is protonated at physiological conditions. The side chain can bear a second phosphonate moiety mimicking pyrophosphate in the active site^30,37^ (see **9c, 9d, 9g, 20, 22**) or a hydroxyl group (**9a**) and thus further contribute to the binding. The appropriate length of the side chain also seems to be important (compare, for example, **9d, 9g** with **9c**), while the addition of the third phosphonate group did not improve the inhibition (derivative **17**).

### Docking studies of ANP-based inhibitors

**Figure 5:**
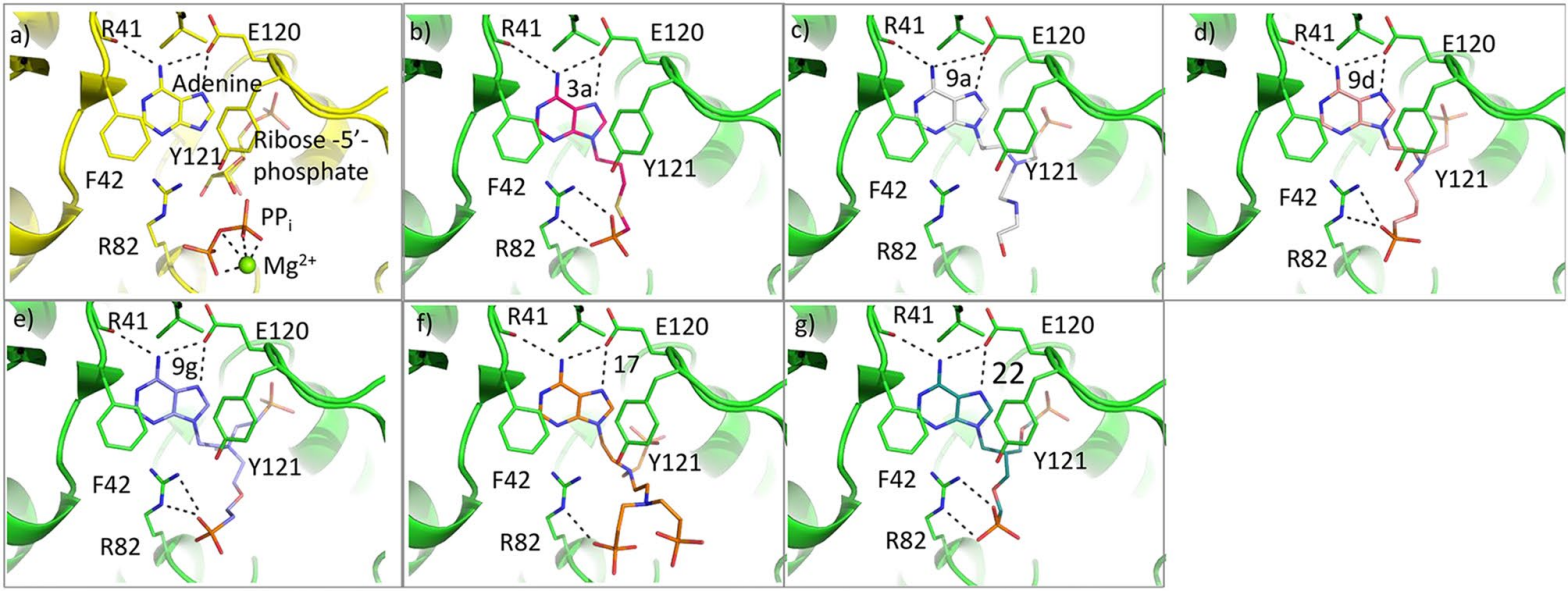
Active site and docking of 6 ANPs with *T. brucei* APRT1. (**a**) The crystal structure of the *T.brucei* APRT1.adenine.ribose-5-phosphate.PP_i_.Mg^2+^ complex (PDB code 5VN4). (**b**–**g**) The six compounds with K_i_ values < 22 μM (**3a**, **9a**, **9d**, **9g**, **17** and **22**, Table 1) docked into this structure. In all docked structures, the adenine base fills the purine binding pocket and makes three hydrogen bonds with the enzyme. The attachments either fill or partially fill the 5-phosphate binding pocket (near E120) or the pyrophosphate binding pocket (near R82). Images were drawn using PyMOL 2.4^49^.

To assess the probable binding modes of the most potent inhibitors, docking calculations were performed. Since *T. brucei* APRT1 has been slightly explored so far, the only experimental structure that is available for this enzyme is APRT1 in complex with adenine and ribose-5-phosphate, pyrophosphate and Mg^2+^ (PDB ID: 5VN4)^41^. We therefore docked the six most potent inhibitors (**3a**, **9a**, **9d**, **9g**, **17** and **22**, Table 1) into this model. The results showed that all of these compounds can fit neatly into the active site with the adenine base adopting an identical position in all docked structures and this is in agreement with its location in the crystal structure. The side-chain of E120 and the carbonyl oxygen of R41 form key hydrogen bonds to the 6-amino group of the purine base, hence accounting for its specificity as an APRT (Fig. 5), in preference to a 6-oxopurine PRT. Another important feature is the presence of F42, which provides pi-stacking interactions with the base. Thus, the base is held in place by a high level of surface complementarity as well by a strong hydrogen bonding network. Since ribose-5-phosphate and pyrophosphate are present in the crystal structure, the pockets that house these sites are in an expanded conformation and are representative of the enzyme under catalysis. It is plausible and likely that in the absence of these ligands, this enzyme adopts different conformations as occurs in other APRTs^42,43^ and closely related 6-oxopurine PRTs^44^. In pre-catalytic structures/conformations the enzyme may not be able to recognize the phosphate/phosphonate moieties or, indeed the adenine base.

**Figure 6:**
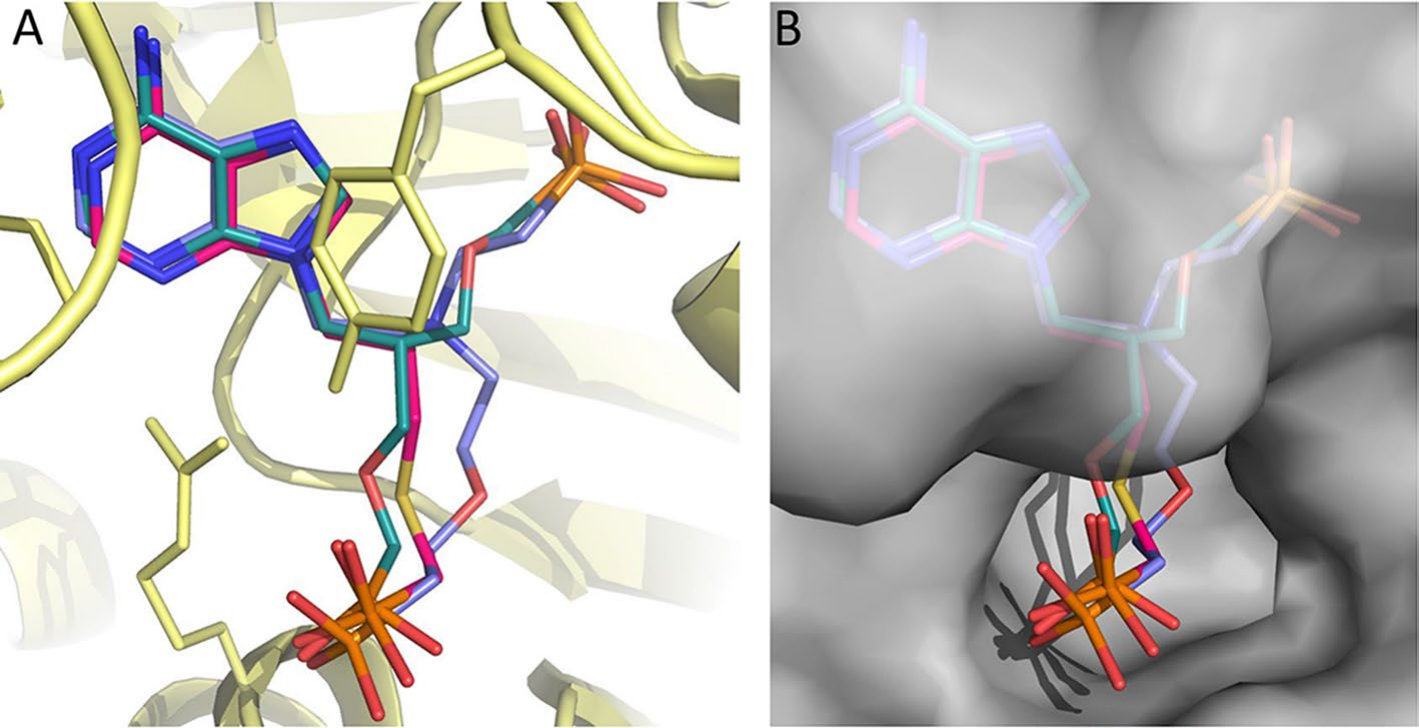
Superimposition of three docked ANPs in the *T. brucei* APRT1 structure (PDB code 5VN4). (**A**)** 3a** (red), **9g** (violet) and **22** (green) shown as stick models and the polypeptide as ribbon structure with the amino acid side chains as sticks. (**B**) The transparent Connolly surface of the enzyme with **3a**, **9g** and **22** show as stick models. Images were drawn using PyMOL 2.4^49^.

The highest docking score (78.5) for **3a** places its phosphonate group in the pocket occupied by pyrophosphate (Fig. 5). However, we cannot completely rule out the possibility that it could prefer to bind in the 5′-phosphate binding pocket especially given that the APRTs have flexible structures. The remaining six compounds all have two (or three, **17**) functional groups attached and thus can potentially fill both the 5-phosphate and pyrophosphate binding sites. The docking scores for **9a**, **9d**, **9g**, **17**, and **22** were 94.17, 112.25, 121.05, 79.9 and 103.85, respectively. The four compounds with the highest docking scores also had the lowest *K*_i_ values and **17,** which has a significantly higher *K*_i_ value 21.3 μM (Table 1), also had the lowest docking score of 79.9. For **22**, the 5-phosphate site is perfectly filled with one of the phosphonates, while the second phosphonate cannot fully reach to the pyrophosphate binding site. A similar docking result occurs for **9d** and **9g**, except the second phosphonate makes a closer approach to the pyrophosphate binding pocket, but again is not optimally placed compared to one of the phosphates when pyrophosphate binds. Compound **9a** is able to extend into both pockets but the hydroxyl group may not be bulky enough and lacks negative charges for optimal binding. For **17**, neither the 5-phosphate site nor the pyrophosphate are fully utilized for binding, consistent with the lower docking scores. Given the good correlation between *K*_i_ values, expected binding modes (based on how the substrates and products bind) and binding modes produced by the docking, the continued use of docking with the GOLD program for further inhibitor design appears as an appropriate strategy. Figure 6 shows a surface representation of the crystal structure of the enzyme with the docking results of **3a**, **9g** and **22** superimposed. The image confirms the similar docking poses achieved by the three distinct chemical classes (thia-ANP, aza-ANP and C-branched ANP). It also highlights the fact that in this complex, the location where the adenine base and 5-phosphate bind are occluded from the solvent. It is therefore necessary that conformational changes would have been required to allow adenine and ribose-5-phosphate to bind to the enzyme. In the resting state, the enzyme likely adopts a more open conformation.

### ANPs based prodrugs exert a cytotoxic activity on *T. brucei* bloodstream through mechanism unrelated to APRT

**Figure 7:**
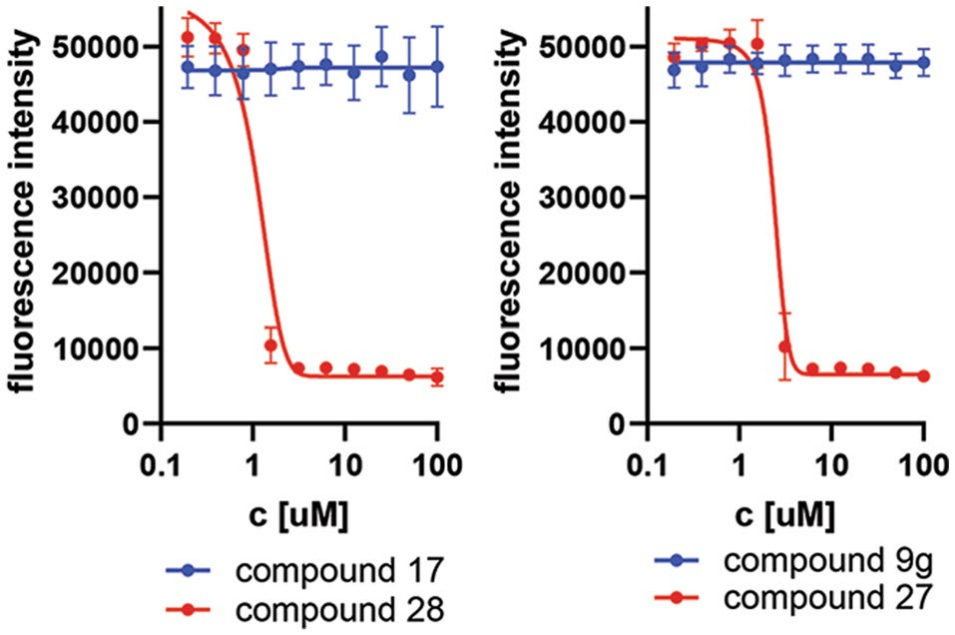
Alamar blue assay of BSF 427 cells treated with free ANPs (compound **17** and **9g**) and with their respective prodrugs (compound **28** and **27**).

The 6-oxopurine-based ANPs displayed cytotoxic effects on *T. brucei* BSF cells^6^. To assess the effect of aminopurine-based ANPs in parasites, some ANPs were tested in the form of their standardly used phosphoramidate prodrugs (Table 1, compounds **24–28**, Scheme 4) that facilitate transport across the plasma membrane and subsequently are cleaved to free phosphonates inside of the cell^39,45^. All the tested prodrugs showed an effect in the single-digit μM range against *T. brucei *in vitro (Table 1) while the respective free-phosphonates did not exert any cytotoxic effects most likely because of the polar character of the phosphonate group that can interfere with their uptake to the cell (Fig. 7). The most potent was compound **28** (prodrug of the parent inhibitor **17**) with an EC_50_ value of < 1 µM, although this compound at 10 µM is also toxic in Normal human dermal fibroblasts (NHDF) (Table 1). All other tested prodrugs **24–27** had no effect on the viability of human NHDF and HeLa S3 cell lines at 10 µM (Table 1).

**Figure 8:**
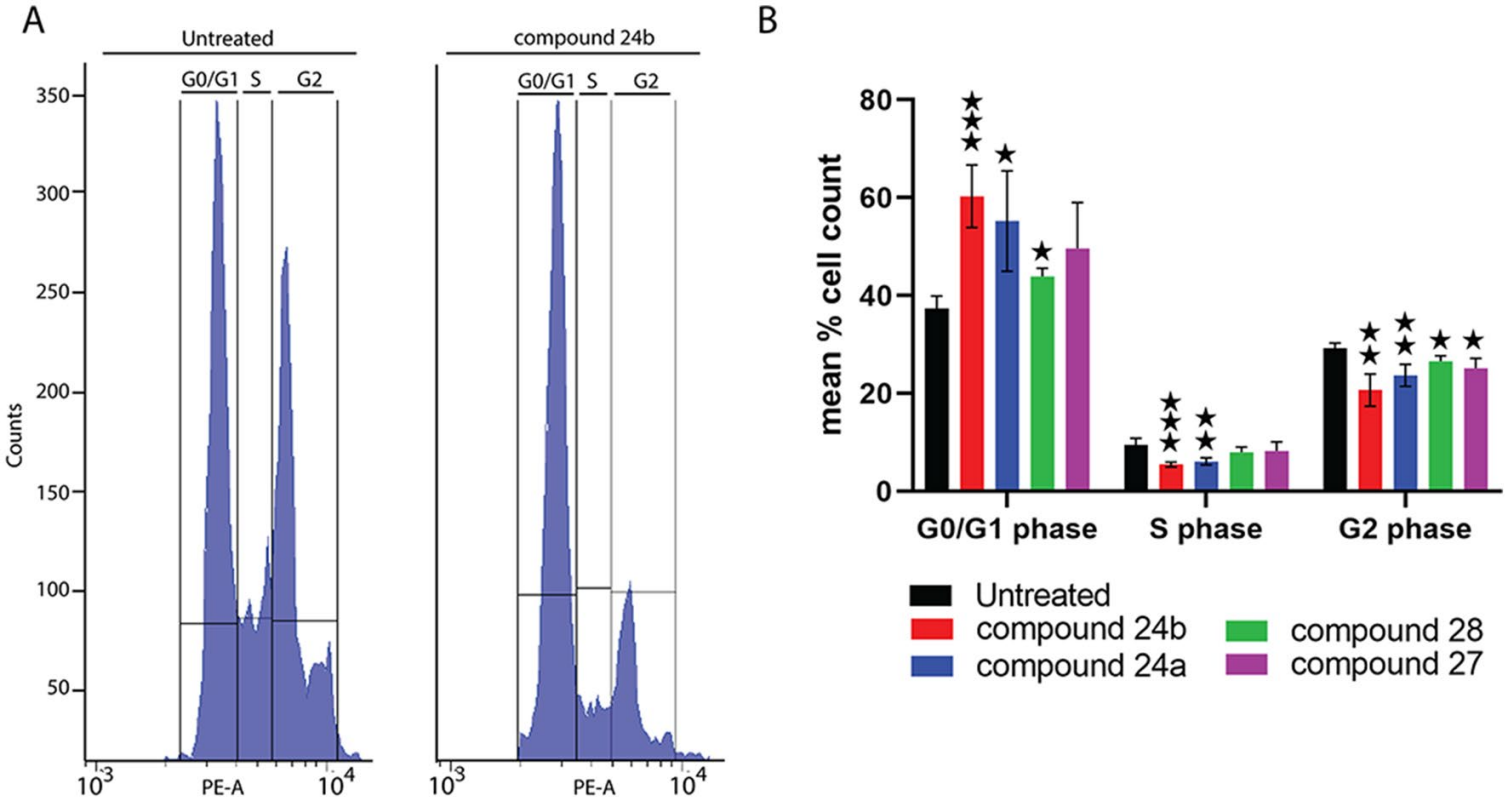
Flow cytometry analysis of DNA content of *T. brucei* cells treated with selected ANPs **24a**, **24b**, **28** and **27** at the concentration ranging from 1 to 16 µM depending on the tested inhibitor. Histograms show the distribution of DNA content of trypanosomes after exposure to selected ANPs. (**A**) Fluorescence intensity in the PE-A channel is shown on the *x* axis and number of cells on the *y* axis (example for **24b**); (**B**) Bar chart displaying the percentage of cell at G0/G1, S and G2 phase. (means ± S.D., n = 3–7, Student’s unpaired test, ***p < 0.001, **p < 0.01, *p < 0.05).

The inhibition of the growth of *T. brucei* BSF in HMI-11 medium in single micromolar EC_50_ values (Table 1) was somewhat surprising considering the dispensability of APRT enzymes for these cells. This observation suggests that while ANPs can inhibit the activity of APRT1 in vitro, this enzyme might not be the primary target of the synthesized inhibitors in vivo. To get deeper insights into the in vivo action of ANPs on APRT enzyme we tested their cytotoxicity on wild type cells grown in HMI-11^adenine^ medium. In this medium, the cells rely on the activity of the APRT enzyme to produce AMP and other nucleosides. We did not detect any significant changes in EC_50_ values between the cells growing in HMI-11 and HMI-11^adenine^. To corroborate this observation, we also tested cells with induced ectopic expression of V5-tagged APRT1 in both types of media and in the presence of the selected ANPs. Again, no difference was found in the measured EC_50_ values (Table 1), further suggesting that APRT enzyme is not the primary target of these compounds in cells. The direct microscopic observation of *T. brucei* cells that were exposed to the inhibitors **24a, 24b, 27** and **28** revealed problems in the cell cycle. The changes in the distribution of different cell cycle stages within a population were further assessed by flow cytometry assaying the DNA content by staining the cells with propidium iodide (Fig. 8A). The most obvious effect was observed for treatment with compounds **24a** and **24b,** which caused an increase in the number of cells in G1-phase, while fewer cells were detected to be in S- and G2-phase (Fig. 8B). Our results suggests that in BSF *T. brucei* the tested ANP prodrugs exert their toxicity by other means than inhibition of the APRT enzymes.

**Table 1:** Ki values of the synthesized ANPs for APRT1 and anti-trypanosomal activity of their prodrugs.

General structure (A = adenin-9-yl)	ANP R=O^−^	APRT1 *K*_i_ [μM]	Prodrug, R =	*T. brucei* BSF EC_50_ (μM)	Cytotoxicity of prodrug: NHDF/HeLaS3 viability (%)^a^
	**3a**	10.1 ± 1.1	**24a**	BSF427, HMI-11 6.16 ± 0.59	89/99
				BSF427, HMI-11^adenine^ 6.36 ± 0.12	
	**3b**	> 30	**24b**	BSF427, HMI-11 6.36 ± 0.95	89/98
				BSF427, HMI-11^adenine^ 6.16 ± 0.09	
	**4a**	> 30	**–**	**–**	**–**
	**4b**	> 30	**–**	**–**	**–**
	**9a**	4.92 ± 0.43	**–**	**–**	**–**
	**9b**	> 30	**–**	**–**	**–**
	**9c**	> 30	**–**	**–**	**–**
	**9d**	3.88 ± 0.29	**–**	**–**	**–**
	**9e**	> 30	**–**	**–**	**–**
	**9f**	> 30	**–**	**–**	**–**
	**9g**	3.07 ± 0.25	**27**	BSF427, HMI-11 2.26 ± 0.69	85/99
				BSF427, HMI-11^adenine^ 3.21 ± 1.56	
				APRT1^OE^, HMI-11 2.26 ± 0.08	
				APRT1^OE^, HMI-11^adenine^ 0.81 ± 0.2	
	**17**	21.3 ± 2.1	**28**	BSF427, HMI-11 0.99 ± 0.24	4/94
				BSF427,HMI-11^adenine^ 0.86 ± 0.31	
				APRT1^OE^, HMI-11 0.86 ± 0.08	
				APRT1^OE^, HMI-11^adenine^ 0.83 ± 0.09	
	**20**	27.7 ± 4.5	**26**	BSF427, HMI-11 1.55 ± 0.07	88/100
	**21**	> 30	**–**	**–**	**–**
	**22**	5.15 ± 0.69	**25**	BSF427, HMI-11 3.7 ± 1.9	90/99

## Conclusions

All medically important unicellular eukaryotic parasites are fully dependent on the purine salvage pathway to synthesize building blocks fo﻿r their DNA and RNA. This dependency represents a potentially promising ground for the discovery of anti-parasitic compounds with a purine-based scaffold. Indeed, we showed that potent 6-oxopurine acyclic nucleoside phosphonates (ANPs) inhibit 6-oxopurine phosphoribosyl transferases in vitro as well as possess strong cytotoxic effects on *T. brucei* bloodstream form cells^6,17,18^. Using our experience with this system, we designed new selective adenine-based ANPs and evaluated their activity in vitro and in *T. brucei* culture. Out of the 15 synthesized ANPs, seven derivatives inhibited adenine phosphoribosyl transferase (APRT) with *K*_i_ values ranging from 3 to 28 μM. Our docking studies using the *T. brucei* APRT1 crystal structure revealed that the synthesized compounds can fit neatly into the active site with the adenine base adopting an identical position in all docked structures and this is in agreement with its location in the crystal structure. Although APRT1 and APRT2 enzymes are dispensable for the growth of BSF *T. brucei* cells under normal conditions, the cell-permeable adenine-based ANP prodrugs displayed anti-trypanosomal activity in the single µM range. These prodrugs can be further subjected to the structure–activity relationship analyses as well as to studies to identify their cellular target(s).

## Methods

### General remarks and methods for synthesis of ANPs and their prodrugs

Unless otherwise stated, the general remarks and methodology A, B, and C was adapted from Ref.^31^. Briefly, solvents were evaporated at 40 °C/2 kPa and prepared compounds were dried at 25–30 °C at 2 kPa. Starting reagents and compounds were purchased from commercial suppliers (Acros Organics, Carbosynth, TCI, Fluorochem, Sigma-Aldrich) and used without further purification or were prepared according to the published procedures.

Analytical TLCs were performed on silica gel pre-coated aluminium plates with fluorescent indicator (Merck 60 F254). Flash column chromatographies were carried out by Teledyne ISCO CombiFlash Rf200 with dual absorbance detector. Various types of columns were used: (a) Teledyne ISCO columns RediSepRf HP Silica GOLD in sizes 12 g, 40 g, 80 g and 120 g; (b) Teledyne ISCO columns RediSepRf HP C18 Aq GOLD in sizes 50 g and 100 g; (c) column Chromabond Flash DL 40, DL 80, DL 120 and DL 200, filled with FLUKA silica gel 60; (d) Interchim puriFlash C18 Aq in sizes F0040 and F0080. Eluents used were cyclohexane-ethyl acetate 6:4 mixture (A), ethyl acetate modified with 10% of methanol (B), chloroform (C), methanol (D) and water (E). Preparative HPLC purifications were performed on Waters Delta 600 chromatography system with columns packed with C18 reversed phase resin (Phenomenex Gemini 10 μm 21 × 250 mm, Phenomenex Gemini 5 μm 21 × 250 mm, Phenomenex Luna 10 μm 21 × 250 mm) using gradient H_2_O/MeOH as eluent. Dowex^®^ 50 W resin was turned to Na^+^ cycle by treatment of Dowex 50 W resin in H^+^ cycle with 1 M NaOH aq. solution, followed by water wash to neutral pH.

Mass spectra, UV absorbance and purity of compounds were measured on Waters UPLC-MS system consisted of Waters UPLC H-Class Core System (column Waters Acquity UPLC BEH C18 1.7 mm, 2.1 × 100 mm), Waters Acquity UPLC PDA detector and Mass spectrometer Waters SQD2. The universal LC method was used (eluent H_2_O/CH_3_CN, gradient 0–100%, run length 7 min) and MS method (ESI+ and/or ESI−, cone voltage = 30 V, mass detector range 100–1000 Da). Purity of the final compounds was > 95%. High-resolution mass spectra were measured on a LTQ Orbitrap XL spectrometer (Thermo Fisher Scientific). NMR spectra were recorded on Bruker Avance 400 or 500 spectrometers referenced to the residual solvent signal.

### Method A: General procedure for Mitsunobu reaction at elevated temperature

In a 60 ml vial triphenylphosphine (2.00 g, 7.5 mmol), adenine (0.77 g, 5.0 mmol) and the corresponding alcohol (6.0 mmol) was suspended in 40 ml of dry dioxane under an argon atmosphere. The suspension was heated up to 80 °C and then DIAD (1.5 ml, 7.5 mmol) was added dropwise via syringe. The mixture got homogeneous and started to colour green in a few minutes. After 30 min, the reaction was quenched with 5 ml of water and was stirred for further 30 min and then the mixture was evaporated. The residue was purified by reverse phase flash chromatography (water/methanol) to yield the title compound as a colourless oil.

### Method B: General procedure for ester cleavage in thia-ANPs

In a 25 ml round-bottom flask the phosphonate compound (1 mmol) was dissolved in 5 ml of dry dichloromethane under an argon atmosphere. Trimethylsilyl bromide (0.5 ml, 3.8 mmol) was added dropwise and the reaction mixture was stirred overnight. After completion, methanol (5 ml) was added and the reaction mixture was evaporated. The residue was dissolved in 10 ml of H_2_O/MeOH (1:1) mixture, stirred for 30 min and evaporated. 2 M TEAB (2.5 ml) was added and the reaction mixture was purified by reverse phase flash chromatography (water/methanol). The fractions containing product were combined, evaporated, dissolved in a minimal amount of water, passed through a short column of DOWEX in Na cycle and lyophilised to yield the product as a white powder.

### Method C: General procedure for oxidation of sulfides to sulfoxides

In a 25 ml round-bottom flask the phosphonate compound (1 mmol) was dissolved in 5 ml of dry dichloromethane under an argon atmosphere. Trimethylsilyl bromide (0.5 ml, 3.8 mmol) was added dropwise and the reaction mixture was stirred overnight. After completion, methanol (5 ml) was added and the reaction mixture was evaporated. The residue was dissolved in 10 ml of H_2_O/MeOH (1:1) mixture, stirred for 30 min and evaporated. 2 M TEAB (2.5 ml) and hydrogen peroxide (0.12 ml) was added and the reaction mixture was stirred for 30 min and then was purified by reverse phase flash chromatography (water/methanol). The fractions containing product were combined, evaporated, dissolved in a minimal amount of water, passed through a short column of DOWEX in Na^+^ cycle and lyophilised to yield the product as a white powder.

### Method D: General procedure for Mitsunobu reaction at RT

To a solution of triphenylphosphine (0.39 g, 1.5 mmol) in dry THF (10 ml) diisopropylazadicarboxylate (DIAD, 0.3 ml, 1.5 mmol) was added slowly under argon atmosphere. The mixture was stirred for 5 min and this preformed complex was added to the mixture of adenine (0.16 g, 1.2 mmol) and corresponding hydroxyderivative (1.0 mmol) in dry THF (10 ml) under argon atmosphere. The resulting mixture was stirred at room temperature for 3 days. Solvent was evaporated and the crude mixture was purified by flash chromatography on silica gel (CHCl_3_–MeOH) followed by preparative HPLC (H_2_O–MeOH).

### Method E: General procedure for ammonolysis of 6-chloropurine

A solution of 6-chloropurine derivative (2 mmol) in methanolic ammonia (60 ml) was stirred in an autoclave at 60 °C for 48 h. Solvent was evaporated and the residue was purified by flash chromatography on silica gel (CHCl_3_–MeOH).

### Method F: General procedure for phosphonate ester cleavage

A mixture of ANP ester (1 mmol), acetonitrile (20 ml) or pyridine (20 ml) and BrSiMe_3_ (0.5 ml for every phosphonate group) was stirred overnight at room temperature. After in vacuo evaporation to dryness, the residue was treated with MeOH/H_2_O for 30 min and again evaporated to dryness. The residue was purified by preparative HPLC (H_2_O–MeOH).

### Method G: General procedure for the synthesis of prodrugs

The phosphonate ester (0.5 mmol) was dissolved in dry dichloromethane, acetonitrile or pyridine (5 ml) under argon atmosphere. BrSiMe_3_ (0.5 ml, 7.8 eq.) was added and the reaction mixture was stirred at r.t. overnight. The solvent was evaporated under argon atmosphere. Isopropyl l-phenylalanine hydrochloride (for every phosphonate group: 0.37 g, 1.5 mmol), dry pyridine (5 ml) and dry triethylamine (for every phosphonate group: 1 ml) was added under argon atmosphere and the mixture was heated to 70 °C. A solution of Aldrithiol (for every phosphonate group: 0.33 g, 1.5 mmol, 3 eq.) and triphenylphosphine (for every phosphonate group: 0.39 g, 1.5 mmol, 3 eq.) in dry pyridine (5 ml) was added and the reaction mixture was stirred at 70 °C for 2–4 days. The solvent was evaporated and the residue was codistilled (toluene, cyclohexane or MeOH) and then purified by column chromatography (40 g SiO_2_, gradient CHCl_3_–MeOH) and preparative HPLC (gradient H_2_O–MeOH).

### Diisopropyl 5-(adenin-9-yl)-2-thiapentanephosphonate (2a)

Prepared by Method A from diisopropyl 5-hydroxy-2-thiapentanephosphonate^31^ (**1a**). Yield 0.68 g (35%).

^1^H NMR (400 MHz, DMSO-*d*_6_) δ 8.14 (s, 1H, H-2), 8.13 (s, 1H, H-8), 7.18 (s, 2H, –N**H**_**2**_), 4.58 (dp, *J* = 7.8, 6.2, 2H, *i*Pr), 4.22 (t, *J* = 6.9, 2H, H-1´), 2.83 (d, *J* = 13.3, 2H, H-4´), 2.64 (t, *J* = 7.2, 2H, H-3´), 2.11 (p, *J* = 7.1, 2H, H-2´), 1.23 (d, *J* = 6.2, 12H, *i*Pr). ^31^P NMR (162 MHz, DMSO-*d*_6_) δ 25.31. ^13^C NMR (101 MHz, DMSO-*d*_6_) δ 156.41 (C-6), 152.82 (C-2), 150.00 (C-4), 141.26 (C-8), 119.22 (C-5), 70.63 (2C, *i*Pr), 42.35 (C-1´), 29.99 (d, *J* = 4.0, C-3´), 29.21 (C-2´), 25.20 (d, *J* = 147.5, C-4´), 24.21 (4C, *i*Pr). MS (ESI+) *m/z* = 388 [M + H]^+^.

### Diethyl 5-(adenin-9-yl)-3-thiapentanephosphonate (2b)

Prepared by Method A from diethyl 5-hydroxy-3-thiapentanephosphonate^31^ (**1b**). Yield 1.00 g (56%).

^1^H NMR (400 MHz, DMSO-*d*_6_) δ 8.17 (s, 1H, H-2), 8.14 (s, 1H, H-8), 7.20 (s, 2H, –N**H**_**2**_), 4.33 (t, *J* = 6.7, 2H, H-1´), 4.05–3.92 (m, 4H, Et), 3.04 (t, *J* = 6.7, 2H, H-2´), 2.69–2.57 (m, 2H, H-3´), 2.06–1.94 (m, 2H, H-4´), 1.23 (t, *J* = 7.0, 6H, Et). ^31^P NMR (162 MHz, DMSO-*d*_6_) δ 30.85. ^13^C NMR (101 MHz, DMSO-*d*_6_) δ 156.39 (C-6), 152.84 (C-2), 149.97 (C-4), 141.47 (C-8), 119.10 (C-5), 61.68 (2C, Et), 42.85 (C-1´), 30.98 (C-2´), 26.09 (d, *J* = 133.8, C-4´), 24.37 (d, *J* = 3.5, C-3´), 16.72 (2C, Et). MS (ESI+) *m/z* = 360 [M + H]^+^.

### 5-(Adenin-9-yl)-2-thiapentanephosphonic acid, disodium salt (3a)

Prepared by Method B from **2a**. Yield 155 mg (89%).

^1^H NMR (400 MHz, D_2_O) δ 8.00 (s, 1H, H-2), 7.94 (s, 1H, H-8), 4.15 (dt, *J* = 13.5, 6.7, 2H, H-1´), 2.55 (d, *J* = 14.0, 2H, H-4´), 2.54–2.47 (m, 2H, H-2´), 2.02 (dt, *J* = 13.6, 6.8, 2H, H-3´). ^31^P NMR (162 MHz, D_2_O) δ 19.99. ^13^C NMR (101 MHz, D_2_O) δ 155.01 (C-6), 151.89 (C-2), 148.39 (C-4), 142.25 (C-8), 118.09 (C-5), 42.61 (C-1´), 29.81 (d, *J* = 6.6, C-3´), 28.23 (C-2´), 27.62 (d, *J* = 135.7, C-4´). MS (ESI+) *m/z* = 304 [M-2 Na + 3 H]^+^. HR-MS (ESI^−^) *m/z*: calcd for C_9_H_13_O_3_N_5_PS = 302.0482 [M − 2Na + H]^−^, found 302.0478.

### 5-(Adenin-9-yl)-3-thiapentanephosphonic acid, disodium salt (3b)

Prepared by Method B from **2b**. Yield 124 mg (72%).

^1^H NMR (400 MHz, D_2_O) δ 8.02 (s, 1H, H-2), 7.99 (s, 1H, H-8), 4.28 (t, *J* = 6.4, 2H, H-1´), 2.92 (t, *J* = 6.4, 2H, H-2´), 2.63–2.50 (m, 2H, H-3´), 1.74–1.60 (m, 2H, H-4´). ^31^P NMR (162 MHz, D_2_O) δ 23.48. ^13^C NMR (101 MHz, D_2_O) δ 155.15 (C-6), 152.09 (C-2), 148.42 (C-4), 142.47 (C-8), 118.07 (C-5), 43.07 (C-1´), 30.70 (C-2´), 28.91 (d, *J* = 127.5, C-4´), 25.69 (d, *J* = 2.1, C-3´). MS (ESI+) *m/z* = 304 [M-2 Na + 3 H]^+^. HR-MS (ESI^−^) *m/z*: calcd for C_9_H_13_O_3_N_5_PS = 302.0482 [M − 2Na + H]^−^, found 302.0479.

### 5-(Adenin-9-yl)-2-oxo-2-thiapentanephosphonic acid, disodium salt (4a)

Prepared by Method C from **3a** Yield 152 mg (84%).

^1^H NMR (400 MHz, D_2_O) δ 7.82 (s, 1H, H-2), 7.73 (s, 1H, H-8), 4.07 (t, *J* = 7.4, 2H, H-1´), 3.22–3.08 (m, 2H, H-3´), 3.05 (dd, *J* = 13.9, 7.5, 1H, H-4´), 2.77 (dd, *J* = 13.9, 7.5, 1H, C-4´), 2.14 (p, *J* = 7.6, 2H, H-2´). ^31^P NMR (162 MHz, D_2_O) δ 8.01. ^13^C NMR (101 MHz, D_2_O) δ 154.55 (C-6), 151.66 (C-2), 147.78 (C-4), 141.63 (C-8), 117.55 (C-5), 58.86 (C-1´), 51.93 (d, *J* = 114.1, C-4´), 42.57 (C-3´), 22.90 (C-2´). MS (ESI−) *m/z* = 318 [M-2 Na + H]^−^. HR-MS (ESI^−^) *m/z*: calcd for C_9_H_13_O_4_N_5_PS = 318.0431 [M − 2Na + H]^−^, found 318.0428.

### Sodium 5-(adenin-9-yl)-3-oxo-3-thiapentanephosphonate (4b)

Prepared by Method C from **3b**. Yield 120 mg (66%).

^1^H NMR (400 MHz, D_2_O) δ 8.02 (s, 1H, H-2), 8.01 (s, 2H, H-8), 4.60 (t, *J* = 5.9, 4H, H-1´), 3.44–3.19 (m, 2H, H-2´), 3.09–2.87 (m, 2H, H-3´), 1.93–1.71 (m, 2H, H-4´).

^31^P NMR (162 MHz, D_2_O) δ 22.10. ^13^C NMR (101 MHz, D_2_O) δ 155.11 (C-6), 152.24 (C-2), 148.45 (C-4), 141.93 (C-8), 118.10 (C-5), 49.81 (C-1´), 46.21 (d, *J* = 2.4 Hz (C-3´)), 38.12 (C-2´), 20.88 (d, *J* = 131.2, C-4´). MS (ESI−) *m/z* = 318 [M-2 Na + H]^−^. HR-MS (ESI^−^) *m/z*: calcd for C_9_H_13_O_4_N_5_PS = 318.0431 [M − 2Na + H]^−^, found 318.0428.

### Diethyl 9-[(*N*-(2-((2-hydroxyethyl)amino)ethyl)-*N*-(2-phosphonoethyl))-2-aminoethyl]adenine (6)

Diethyl 9-[(*N*-(2-hydroxyethyl)-*N*-(2-phosphonoethyl))-2-aminoethyl]adenine^32^ (**5**, 1.3 g, 3.37 mmol), tosyl chloride (1.0 g, 5.27 mmol) and DMAP (30 mg) in dry pyridine (20 ml) were stirred for 2 days at room temperature. Solvent was evaporated and the residue was purified by column chromatography (40 g SiO_2_, gradient CHCl_3_–MeOH). The crude product (0.32 g, 0.59 mmol) was dissolved in MeOH (5 ml) and 2-aminoethanol (1 ml) was added. The mixture was stirred for 3 days at 60 °C, evaporated and codistilled with methanol to dryness. The residue was purified by preparative HPLC (H_2_O-MeOH), yield 0.19 g (75%).

^1^H NMR (400 MHz, DMSO-*d*_6_) δ 8.16 (s, 1H, H-2), 8.12 (s, 1H, H-8), 7.16 (s, 2H, NH_2_), 4.40 (t, *J* = 4.7, 1H, OH); 4.11 (m, 1H, NH), 4.16 (t, *J* = 6.1, 2H, H-1′), 3.93 (m, 4H, Et), 3.38 (m, 2H, H-8′), 2.80 (t, *J* = 6.1, 2H, H-2′), 2.65 (m, 2H, H-3′), 2.43 (m, 6H, H-5′, H-6′ and H-7′), 1.75 (m, 2H, H-4′), 1.20 (t, 6H, Et). ^13^C NMR (101 MHz, DMSO-*d*_6_) δ 155.79 (C-6), 152.12 (C-2), 149.39 (C-4), 141.25 (C-8), 118.54 (C-5), 60.76 (2C, Et), 60.32 (C-8′), 52.98, 52.21 and 51.64 (C-2′, C-3′, C-5′), 46.99 and 46.91 (C-6′ and C-7′), 41.39 (C-1′), 22.07 (d, *J* = 134.1, C-4′), 16.14 (2C, Et). MS (ESI^+^) *m/z* = 430 [M + H]^+^.

### Diethyl 9-[(*N*-(2-cyanoethyl)-*N*-(2-phosphonoethyl))-2-aminoethyl]adenine (8a)

Prepared by Method E, starting from diethyl 9-[(*N*-(2-cyanoethyl)-*N*-(2-phosphonoethyl))-2-aminoethyl]-6-chloropurine^33^ (**7a**, 2.1 g, 4.87 mmol), yield 0.83 g (43%).

^1^H NMR (400 MHz, DMSO-*d*_6_) δ 8.15 (s, 1H, H-2), 8.13 (s, 1H, H-8), 7.18 (s, 2H, NH_2_), 4.17 (t, *J* = 6.2, 2H, H-1′), 3.92 (m, 4H, Et), 2.86 (t, *J* = 6.2, 2H, H-2′), 2.77 (t, *J* = 6.8, 2H, H-5′), 2.66 (m, 2H, H-3′), 2.53 (t, *J* = 6.7, 2H, H-6′), 1.73 (m, 2H, H-4′), 1.19 (t, *J* = 7.1, 6H, Et). ^13^C NMR (101 MHz, DMSO-*d*_6_) δ 155.79 (C-6), 152.14 (C-2), 149.37 (C-4), 141.06 (C-8), 119.75 (CN), 118.53 (C-5), 60.79 (2C, Et), 51.57, 48.29 and 46.44 (C-2′, C-3′ and C-5′), 41.18 (C-1′), 22.42 (d, *J* = 134.2, C-4′), 16.10 (2C, Et). MS (ESI^+^) *m/z* = 396 [M + H]^+^.

### Tetraethyl 9-[(*N*,*N*-(bis-2-phosphonoethyl))-2-aminoethyl]adenine (8b)

Prepared by Method E, starting from tetraethyl 9-[(*N*,*N*-(bis-2-phosphonoethyl))-2-aminoethyl]-6-chloropurine^33^ (**7b**, 1.04 g, 1.98 mmol), yield 0.54 g (54%).

^1^H NMR (400 MHz, DMSO-*d*_6_) δ 8.18 (s, 1H, H-2), 8.12 (s, 1H, H-8), 7.16 (s, 2H, NH_2_), 4.16 (t, *J* = 6.0, 2H, H-1′), 3.93 (m, 8H, Et), 2.80 (t, *J* = 6.0, 2H, H-2′), 2.66 (m, 4H, H-3′ and H-5′), 1.73 (m, 4H, H-4′ and H-6′), 1.20 (t, 12H, Et). ^13^C NMR (101 MHz, DMSO-*d*_6_) δ 155.77 (C-6), 152.07 (C-2), 149.38 (C-4), 141.22 (C-8), 118.51 (C-5), 60.74 (4C, Et), 51.33 (C-2′), 46.22 (2C, C-3′ and C-5′), 41.20 (C-1′), 22.36 (d, *J* = 134.7, 2C, C-4′ and C-6′), 16.08 (4C, Et). MS (ESI^+^) *m/z* = 507 [M + H]^+^.

### 9-[(*N*-(Diethyl)phosphonoethyl-*N*-(diisopropyl)-phosphonomethoxyethyl)-2-aminoethyl]adenine (8c)

Prepared by Method E, starting from 9-[(*N*-(diethyl)phosphonoethyl-*N*-(diisopropyl)-phosphonomethoxyethyl)-2-aminoethyl]-6-chloropurine^30^ (**7c**, 0.5 g, 0.86 mmol), yield 0.41 g (85%).

^1^H NMR (400 MHz, DMSO-*d*_6_) δ 8.14 (s, 1H, H-2), 8.12 (s, 1H, H-8), 7.16 (s, 2H, NH_2_), 4.58 (m, 2H, iPr), 4.15 (t, *J* = 6.1, 2H, H-1′), 3.92 (m, 2H, Et), 3.67 (d, *J* = 8.1, 2H, H-7′), 3.47 (t, *J* = 5.7, 2H, H-6′), 2.85 (t, *J* = 6.1, 2H, H-2′), 2.66 (m, 4H, H-3′ and H-5′), 1.72 (m, 2H, H-4′), 1.22 (m, 18H, Et and iPr). ^13^C NMR (101 MHz, DMSO-*d*_6_) δ 155.79 (C-6), 152.11 (C-2), 149.39 (C-4), 141.22 (C-8), 118.52 (C-5), 70.91 (d, *J* = 11.4, C-6′), 69.95 (2C, iPr), 64.78 (d, *J* = 164.4, C-7′), 60.73 (2C, Et), 52.45, 52.05 and 47.47 (C-2′, C-3′ and C-5′), 41.33 (C-1′), 23.69 (4C, iPr), 22.48 (d, *J* = 134.0, C-4′), 16.13 (2C, Et). MS (ESI^+^) *m/z* = 565 [M + H]^+^.

### Diethyl 9-[(*N*-(4-amino-4-oxobutyl)-*N*-(2-phosphonoethyl))-2-aminoethyl]adenine (11) and Diethyl 9-[(*N*-(carboxypropyl)-*N*-(2-phosphonoethyl))-2-aminoethyl]adenine (12)

Diethyl 9-[(*N*-(4-methoxy-4-oxobutyl)-*N*-(2-phosphonoethyl))-2-aminoethyl]-6-chloropurine^33^ (**10**, 1.25 g, 2.7 mmol) was dissolved in THF (30 ml) and aqueous NH_3_ (40 ml) and the mixture was vigorously stirred for 5 days. Solvents were evaporated in vacuo. The residue was dissolved in MeOH–H_2_O (10 ml), acidified by HCl and applied on the column of Dowex 50 (H^+^). Column was washed by MeOH–H_2_O (1:1) and product was eluted by MeOH–H_2_O–NH^3^ (1:1:0.2). The crude mixture of the both products was used directly for the synthesis of **9e** and **9f**.

### 9-[(*N*-(Diethyl)phosphonoethyl-*N*-(diisopropyl)-phosphonoethoxyethyl)-2-aminoethyl]adenine (14)

Prepared by Method D, starting from adenine and [(*N*-(diethyl)phosphonoethyl-*N*-(diisopropyl)phosphonoethoxyethyl]-2-aminoethanol^30^ (**13**, 1.2 g, 2.77 mmol), yield 0.75 g (49%).

^1^H NMR (400 MHz, DMSO-*d*_6_) δ 8.14 (s, 1H, H-2), 8.12 (s, 1H, H-8), 7.13 (s, 2H, NH_2_), 4.53 (m, 2H, iPr), 4.15 (t, *J* = 6.1, 2H, H-1′), 3.92 (m, 4H, Et), 3.47 and 3.31 (2xm, 2 × 2H, H-6′ and H-7′), 2.84 (t, *J* = 6.1, 2H, H-2′), 2.67 (m, 2H, H-3′), 2.62 (t, *J* = 5.8, 2H, H-5′), 1.95 (m, 2H, H-8′), 1.73(m, 2H, H-4′), 1.22 (m, 12H, iPr), 1.19 (t, 6H, Et). ^31^P NMR (162 MHz, DMSO-*d*_6_) δ 32.79 and 28.70. ^13^C NMR (101 MHz, DMSO-*d*_6_) δ 155.90 (C-6), 152.21 (C-2), 149.52 (C-4), 141.40 (C-8), 118.61 (C-5), 69.36 (2C, iPr), 69.59 and 64.49 (C-6′ and C-7′), 60.84 (2C, Et), 52.55, 52.18 and 47.52 (C-2′, C-3′ and C-5′), 41.37 (C-1′), 27.23 (d, *J* = 138.4, C-8′), 23.73 (4C, iPr), 22.39 (d, *J* = 134.0, C-4′), 16.27 (2C, Et). MS (ESI^+^) *m/z* = 579 [M + H]^+^.

### Diethyl (2-((2-((2-(adenin-9-yl)ethyl)(2-(diethoxyphosphoryl)ethyl)amino)ethyl)(2-(diethoxyphosphoryl)-ethyl)amino)ethyl)phosphonate (16)

Prepared by Method D, starting from adenine and tetraethyl (((2-((2-(diethoxyphosphoryl)ethyl)(2-hydroxyethyl)-amino)ethyl)azanediyl)bis(ethane-2,1-diyl))bis(phosphonate)^34^ (**15**, 4 g, 6.7 mmol), yield 1.18 g (25%).

^1^H NMR (400 MHz, DMSO-*d*_6_) δ 8.15 (s, 1H, H-2), 8.12 (s, 1H, H-8), 7.13 (s, 2H, NH_2_), 4.16 (t, *J* = 6.2, 2H, H-1′), 3.97 (m, 12H, Et), 2.82 (t, *J* = 6.2, 2H, H-2′), 2.67, 2.59, 2.45 and 2.31 (4xm, total 10H, H-3′, H-5′, H-6′, H-7′ and H-9′), 1.82 (m, 4H, H-4′ and H-8′). ^31^P NMR (162 MHz,) δ 33.01 (2P) and 32.82. ^13^C NMR (101 MHz, DMSO-*d*_6_) δ 156.38 (C-6), 152.70 (C-2), 150.02 (C-4), 141.80 (C-8), 119.10 (C-5), 61.35 (6C, Et), 52.79, 52.64, 51.32 50.57, 47.81 and 46.65 (C-2′, C-3′, C-5′, C-6′, C-7′ and C-9′), 41.80 (C-1′), 22.80 (d, *J* = 135.4, C-4′). 22.65 (d, *J* = 135.3, 2C, C-8′ and C-10′). MS (ESI^+^) *m/z* = 714 [M + H]^+^.

### 9-[(*N*-(2-((2-Hydroxyethyl)amino)ethyl)-*N*-(2-phosphonoethyl))-2-aminoethyl]adenine triethylammonium salt (9a)

Prepared by Method B, starting from **6** (0.04 g, 0.1 mmol), isolated as triethylammonium salt using HPLC (TEAB-CH_3_CN) for purification, yield 0.05 g (87%).

^1^H NMR (400 MHz, D_2_O) δ 8.19 (s, 1H, H-2), 8.18 (s, 1H, H-8), 4.33 (t, *J* = 6.3, 2H, H-1′), 3.60 (m, 2H, H-8′), 2.95 (t, *J* = 6.3, 2H, H-2′), 2.82 (m, 2H, H-3′), 2.64 (m, 6H, H-5′, H-6′ and H-7′), 1.55 (m, 2H, H-4′), 1.25 (m, Et_3_N). ^13^C NMR (101 MHz, D_2_O) δ 155.11 (C-6), 151.99 (C-2), 148.45 (C-4), 142.60 (C-8), 118.00 (C-5), 58.11 (C-8′), 51.37, 50.23, 49.05, 48.46 and 44.80 (C-2′, C-3′, C-5′, C-6′ and C-7′), 46.25 (Et_3_N), 41.38 (C-1′), 24.95 (d, *J* = 125.71, C-4′), 7.87 (Et_3_N). MS (ESI^−^) *m/z* = 372 [M − H]^−^. HR-MS (ESI^+^) *m/z*: calcd for C_13_H_25_O_4_N_7_P = 374.17002 [M + H]^+^, found 374.17011.

### 9-[(*N*-(2-Cyanoethyl)-*N*-(2-phosphonoethyl))-2-aminoethyl]adenine (9b)

Prepared by Method F, starting from **8a** (0.4 g, 1 mmol), yield 0.28 g (82%).

^1^H NMR (400 MHz, DMSO-*d*_6_) δ 8.16 (s, 1H, H-2), 8.13 (s, 1H, H-8), 7.18 (s, 2H, NH_2_); 4.18 (t, *J* = 6.2, 2H, H-1′), 2.80 (t, *J* = 6.2, 2H, H-2′), 2.72 (m, 4H, H-3′ and H-5′), 2.48 (m, 2H, H-6′), 1.48 (m, 2H, H-4′). ^13^C NMR (101 MHz, DMSO-*d*_6_) δ 155.72 (C-6), 152.14 (C-2), 149.35 (C-4), 141.13 (C-8), 119.73 (CN), 118.48 (C-5), 51.79, 48.33 and 47.71 (C-2′, C-3′ and C-5′), 41.07 (C-1′), 25.58 (d, *J* = 130.3, C-4′), 15.51 (C-6′). MS (ESI^−^) *m/z* = 338 [M − H]^−^. HR-MS (ESI^+^) *m/z*: calcd for C_12_H_19_O_3_N_7_P = 340.12815 [M + H]^+^, found 340.12811.

### 9-[(*N*,*N*-(Bis-2-phosphonoethyl))-2-aminoethyl]adenine (9c)

Prepared by Method F, starting from **8b** (0.52 g, 1.03 mmol), yield 0.29 g (72%).

^1^H NMR (400 MHz, DMSO-*d*_6_) δ 8.36 (s, 1H, H-2), 8.29 (s, 1H, H-8), 7.98 (s, 2H, NH_2_); 4.58 (t, *J* = 6.3, 2H, H-1′), 3.58 (t, *J* = 6.3, 2H, H-2′), 3.30 (m, 4H, H-3′ and 5′), 2.04 (m, 4H, H-4′ and H-6′). ^13^C NMR (101 MHz, DMSO-*d*_6_) δ 153.80 (C-6), 149.93 (C-2), 149.07 (C-4), 141.88 (C-8), 118.31 (C-5), 49.89 (C-2′), 47.58 (2C, C-3′ and C-5′), 38.18 (C-1′), 22.67 (d, *J* = 130.6, 2C, C-4′ and C-6′). MS (ESI^−^) *m/z* = 393 [M − H]^−^. HR-MS (ESI^+^) *m/z*: calcd for C_11_H_21_O_6_N_6_P = 395.09923 [M + H]^+^, found 395.09914.

### 9-[(*N*-Phosphonoethyl-*N*-phosphonomethoxyethyl)-2-aminoethyl]adenine (9d)

Prepared by Method F, starting from **8c** (0.4 g, 0.7 mmol), yield 0.22 g (74%).

^1^H NMR (400 MHz, D_2_O) δ 8.47 (s, 1H, H-2), 8.41 (s, 1H, H-8), 4.83 (t, *J* = 6.6, 2H, H-1′), 3.93 (t, *J* = 4.7, 2H, H-6′), 3.87 (t, *J* = 6.6, 2H, H-2′), 3.67 (d, *J* = 8.8, 2H, H-7′), 3.57 (t, *J* = 5.0, 2H, H-5′), 3.53 (m, 2H, H-3′), 2.05 (m, 2H, H-4′). ^13^C NMR (101 MHz, D_2_O) δ 149.47 (C-6), 148.48 (C-2), 144.33 (C-4), 144.28 (C-8), 118.00 (C-5), 66.47 (d, *J* = 157.0, C-7′), 65.25 (d, *J* = 12.2, C-6′), 52.76, 50.70 and 49.82 (C-2′, C-3′ and C-5′), 38.77 (C-1′), 22.76 (d, *J* = 130.6, C-4′). MS (ESI^−^) *m/z* = 423 [M − H]^−^. HR-MS (ESI^+^) *m/z*: calcd for C_12_H_23_O_7_N_6_P_2_ = 425.10980 [M + H]^+^, found 425.10957.

### 9-[(*N*-(4-Amino-4-oxobutyl)-*N*-(2-phosphonoethyl))-2-aminoethyl]adenine (9e) and 9-[(*N*-(Carboxypropyl)-*N*-(2-phosphonoethyl))-2-aminoethyl]adenine (9f)

Prepared by Method F, starting from crude mixture of **11** and **12**. Yield: amide **9e** 0.4 g (40%), carboxylic acid **9f.** 0.16 (16%).

amide **9e**: ^1^H NMR (400 MHz, DMSO-*d*_6_) δ 8.17 (s, 1H, H-2), 8.14 (s, 1H, H-8), 7.30 and 6.77 (2xs, 2 × 1H, NH_2_), 7.24 (s, 2H, NH_2_); 4.29 (t, *J* = 6.2, 2H, H-1′), 3.02 (t, *J* = 6.2, 2H, H-2′), 2.88 (m, 2H, H-3′), 2.61 (t, *J* = 7.0, 2H, H-5′), 2.02 (t, *J* = 7.2, 2H, H-7′), 1.66 (m, 2H, H-4′), 1.56 (m, 2H, H-6′). ^13^C NMR (101 MHz, DMSO-*d*_6_) δ 173.82 (CO), 155.74 (C-6), 152.14 (C-2), 149.35 (C-4), 141.07 (C-8), 118.49 (C-5), 51.87, 51.61 and 48.00 (C-2′, C-3′ and C-5′), 40.23 (C-1′), 32.18 (C-7′), 24.21 (d, *J* = 136.5, C-4′), 21.56 (C-6′). MS (ESI^−^) *m/z* = 370 [M − H]^−^. HR-MS (ESI^−^) *m/z*: calcd for C_13_H_21_O_4_N_7_P = 370.13981 [M − H]^−^, found 370.13938.

carboxylic acid **9f**: ^1^H NMR (400 MHz, DMSO-*d*_6_) δ 8.15 (s, 1H, H-2), 8.14 (s, 1H, H-8), 7.24 (s, 2H, NH_2_); 4.29 (t, *J* = 6.1, 2H, H-1′), 3.02 (t, *J* = 6.1, 2H, H-2′), 2.67 (m, 2H, H-3′), 2.62 (t, *J* = 7.0, 2H, H-5′), 2.14 (t, *J* = 7.1, 2H, H-7′), 1.67 (m, 2H, H-4′), 1.57 (m, 2H, H-6′). ^13^C NMR (101 MHz, DMSO-*d*_6_) δ 174.06 (CO), 155.72 (C-6), 152.10 (C-2), 149.35 (C-4), 141.05 (C-8), 118.50 (C-5), 51.55, 51.51 and 47.88 (C-2′, C-3′ and C-5′), 40.25 (C-1′), 30.88 (C-7′), 24.22 (d, *J* = 130.6, C-4′), 21.09 (C-6′). MS (ESI^−^) *m/z* = 371 [M − H]^−^. HR-MS (ESI^−^) *m/z*: calcd for C_13_H_20_O_5_N_6_P = 371.12383 [M − H]^−^, found 371.12334.

### 9-[(*N*-Phosphonoethyl-*N*-phosphonoethoxyethyl)-2-aminoethyl]adenine (9 g), tetrasodium salt

Prepared by Method F, starting from **14** (0.32 g, 0.55 mmol). The HPLC fraction containing product was passed through a short column of Dowex 50 in Na^+^ cycle to obtain the product as soluble tetrasodium salt, yield 0.14 g (48%).

^1^H NMR (400 MHz, D_2_O) δ 8.32 (s, 1H, H-2), 8.27 (s, 1H, H-8), 4.77 (m, 2H, H-1′), 3.84 (m, 4H, H-6′ and H-7′), 3.68 (m, 2H, H-3′), 3.55 (m, 4H, H-2′ and H-5′), 2.09 (m, 2H, H-4′), 1.86 (m, 2H, H-8′). ^31^P NMR (162 MHz, DMSO-*d*_6_) δ 23.74 and 19.95. ^13^C NMR (101 MHz, D_2_O) δ 154.04 (C-6), 150.46 (C-2), 148.89 (C-4), 142.94 (C-8), 118.40 (C-5), 66.45 (C-6′), 63.37 (C-7′), 52.88, 51.47 and 50.18 (C-2’, C-3′ and C-5′), 39.12 (C-1′), 28.34 (d, *J* = 131.7, C-8′’). 22.45 (d, *J* = 129.1, C-4′). MS (ESI^+^) *m/z* = 439 [M + H]^+^. HR-MS (ESI^+^) *m/z*: calcd for C_13_H_25_O_7_N_6_P_2_ = 439.12545 [M + H]^+^, found 439.12521.

### (2-((2-((2-(Adenine-9-yl)ethyl)(2-phosphonoethyl)amino)ethyl)(2-phosphonoethyl)amino)ethyl)phosphonic acid (17), hexasodium salt

Prepared by Method F, starting from **16** (0.36 g, 0.5 mmol). The HPLC fraction containing product was passed through a short column of Dowex 50 in Na^+^ cycle to obtain the product as soluble hexasodium salt, yield 0.195 g (58%).

^1^H NMR (400 MHz, D_2_O) δ 8.26 (s, 1H, H-2), 8.24 (s, 1H, H-8), 4.39 (t, *J* = 6.4, 2H, H-1′), 3.32 (m, 4H, H-7′ and H-9′), 3.13 (t, *J* = 6.4, 2H, H-2′), 3.06 and 2.93 (2xt, *J* = 6.4, 2 × 2H, H-5′ and H-6′), 2.88 (m, 2H, H-3′), 1.97 (m, 4H, H-8′ and H-10′), 1.74 (m, 2H, H-4′). ^31^P NMR (162 MHz, D_2_O) δ 25.22 and 19.84 (2P). ^13^C NMR (101 MHz, D_2_O) δ 155.34 (C-6), 152.20 (C-2), 148.85 (C-4), 142.88 (C-8), 118.33 (C-5), 51.81, 49.82, 47.55 and 47.20 (C-2′, C-3′, C-5′ and C-6′), 48.55 (2C, C-7′ and C-9′), 41.55 (C-1′), 24.15 (d, *J* = 129.4, C-4′). 22.61 (d, *J* = 128.4, 2C, C-8′ and C-10′). MS (ESI^+^) *m/z* = 546 [M + H]^+^. HR-MS (ESI^−^) *m/z*: calcd for C_15_H_29_O_9_N_7_P_3_ = 544.12451 [M − H]^−^, found 544.12279.

### Diethyl {2-[3-(adenin-9-yl)-2-(2-diethoxyphosphoryl)ethoxy)propoxy]ethyl}phosphonate (19)

Prepared by Method E sarting from diethyl{2-[3-(6-chloropurine-9-yl)-2-(2-(diethoxyphosphoryl)ethoxy)propoxy]ethyl}phosphonate^37^ (**18**, 2.6 g, 4.6 mmol). The product (yellow oil (1.51 g, 61%) was used directly for the preparation of compound **20**. MS (ESI^+^) *m/z* = 538 [M + H]^+^.

### 4-(Adenin-9-ylmethyl)-3,6-dioxaoctane-1,8-diphosphonic acid (20)

Prepared by Method F, starting from **19** (0.7 g, 1.3 mmol), yield 0.11 g (19%).

^1^H NMR (400 MHz, D_2_O) δ 8.14 (s, 1H, H-2), 8.05 (s, 1H, H-8), 4.39 (dd, *J* = 14.8, 4.3, 1H, H-1′), 4.25 (dd, *J* = 14.9, 6.3, 1H, H-1′), 3.88 (p, *J* = 4.8, 1H, H-2′), 3.73–3.49 (m, 5H, H-3′, H-5′, H-6′), 3.34 (dd, *J* = 10.8, 5.1, 1H, H-5′), 1.73–1.61 (m, 2H, H-7′), 1.60–1.48 (m, 2H, H-4′). ^31^P NMR (162 MHz, D_2_O) δ 19.30, 18.93. ^13^C NMR (101 MHz, D_2_O) δ 155.51 (C-6), 152.50 (C-2), 149.20 (C-4), 143.19 (C-8), 118.19 (C-5), 75.90 (C-2′), 68.98 (C-5′), 68.74 (d, *J* = 3.2, C-6′), 67.51 (d, *J* = 3.9, C-3′), 44.10 (C-1′), 30.36 (d, *J* = 123.4, C-7′), 30.00 (d, *J* = 124.4, C-4′). MS (ESI^+^) *m/z* = 426 [M + H]^+^. HR-MS (ESI^−^) *m/z*: calcd for C_12_H_20_O_8_N_5_P_2_ = 424.07926 [M − H]^−^, found 424.07863.

### 9-[2-(3-Hydroxy-1-phosphonopropan-2-yloxy)ethyl]adenine (21)

ANP **21** was prepared previously^35^.

### {[(2-[(Adenin-9-yl)methyl]propane-1,3-diyl)bis(oxy)]bis(methylene)}diphosphonic acid (22)

Starting from **23**, prepared previously^36^.

### 9-(5-(Bis((S-1-isopropoxycarbonyl-2-phenylethyl)amino)phosphoryl)-4-thiapentyl)adenine (24a)

Prepared by Method G from **2a**. Yield 224 mg (66%).

^1^H NMR (400.0 MHz, DMSO-*d*_6_): 8.15 (s, 1H, H-2), 8.14 (s, 1H, H-8), 7.31–7.11 (m, 12H, H-*o,m,p*-Ph, ArNH_2_), 4.81, 4.78 (2 × sep, 2 × 1H, *J*_vic_ = 6.3, iPr), 4.47 (t, 1H, *J*_H,P_ = *J*_NH,2_ = 11.3, NH-Phe), 4.26 (t, 1H, *J*_H,P_ = *J*_NH,2_ = 10.7, NH-Phe) 4.20 (t, 2H, *J*_vic_ = 7.0, H-1′), 4.06–3.84 (m, 2H, PNHCH), 2.94–2.78 (m, 4H, PhCH_2_), 2.55 (t, *J* = 7.0, 2H, H-3′S), 2.37 (qd, *J* = 14.6, 12.1, 2H, PCH_2_), 2.01 (p, *J* = 7.1, 2H, H-2′), 1.16, 1.12, 1.07, 1.02 (4 × d, 4 × 3H, *J*_vic_ = 6.2, *i*Pr). ^31^P{^1^H} NMR (167 MHz, DMSO-*d*_6_): 24.90. ^13^C NMR (101 MHz, DMSO-*d*_6_): 172.3 (d, *J*_C,P_ = 3.2, C-1-Phe), 172.1 (d, *J*_C,P_ = 3.2, C-1-Phe), 156.0 (C-6), 152.4 (C-2), 149.5 (C-4), 140.8 (CH-8), 137.1, 137.0 (C-*i*-Ph), 129.5, 129.5 (CH-*o*-Ph), 128.1, 128.1 (CH-*m*-Ph), 126.5, 126.4 (CH-*p*-Ph), 118.8 (C-5), 68.0, 67.8 (iPr), 54.2, 54.2 (CH-2-Phe), 41.9 (C-1′), 40.1, 40.0 (CH_2_-3-Phe), 29.6 (d, *J*_C,P_ = 4.4, C-3′), 28.9 (C-2′), 28.1 (d, *J*_C,P_ = 117.0, C-4′), 21.5, 21.5, 21.4, 21.3 (*i*Pr). MS (ESI^+^) *m/z* = 682 [M + H]^+^. HR-MS (ESI^+^) *m/z*: calcd for C_33_H_45_O_5_N_7_PS = 682.2935 [M + H]^+^, found 682.2932.

### 9-(5-(Bis((S-1-isopropoxycarbonyl-2-phenylethyl)amino)phosphoryl)-3-thiapentyl)adenine (24b)

Prepared by Method G from **2b**. Yield 118 mg (35%).

^1^H NMR (400.0 MHz, DMSO-*d*_6_): 8.15 (s, 1H, H-2), 8.13 (s, 1H, H-8), 7.32–7.10 (m, 12H, H-*o,m,p*-Ph, ArNH_2_), 4.83, 4.80 (2 × sep, 2 × 1H, *J*_vic_ = 6.3, *i*Pr), 4.58 (t, 1H, *J*_H,P_ = *J*_NH,2_ = 12.2, NH-Phe), 4.28 (t, 2H, *J*_vic_ = 6.9, H-1′), 4.15 (dd, 1H, *J*_H,P_ = 12.9, *J*_NH,2_ = 10.5, NH-Phe), 3.97 (p, 1H, *J* = 8.5, PNHCH), 3.85 (p, 1H, *J* = 8.1, PNHCH), 2.96–2.82 (m, 5H, PhCH_2_ + H-2′), 2.77–2.69 (m, 1H, H-2′), 2.47–2.31 (m, 2H, H-3′), 1.70–1.43 (m, 2H, H-4′), 1.18, 1.13, 1.08, 1.02 (4 × d, 4 × 3H, *J*_vic_ = 6.2, *i*Pr). ^31^P{^1^H} NMR (202.4 MHz, DMSO-*d*_6_): 27.67. ^13^C NMR (101 MHz, DMSO-*d*_6_): 172.6 (d, *J*_C,P_ = 3.2, C-1-Phe), 172.5 (d, *J*_C,P_ = 3.2, C-1-Phe), 156.0 (C-6), 152.4 (CH-2), 148.5 (C-4), 140.9 (CH-8), 137.2 (C-*i*-Ph), 129.5, 129.4 (CH-*o*-Ph), 128.1, 128.1 (CH-*m*-Ph), 126.5, 126.4 (CH-*p*-Ph), 118.7 (C-5), 67.9, 67.7 (*i*Pr), 54.3, 53.9 (CH-2-Phe), 42.3 (C-1′), 40.0, 40.0 (CH_2_-3-Phe), 30.2 (C-2′), 29.8 (d, *J*_C,P_ = 108.8, C-4′), 24.3 (C-3′), 21.5, 21.4, 21.4, 21.3 (*i*Pr). HR-MS (ESI^+^) *m/z*: calcd for C_33_H_44_O_5_N_7_NaPS = 704.2755 [M + Na]^+^, found 704.2758.

### Tetra-(l-phenylalanine ethyl ester) prodrug of {[(2-[(adenin-9-yl)methyl]propane-1,3-diyl)bis(oxy)]bis(methylene)}diphosphonic acid (25)

Prepared by Method G starting from of tetraisopropyl {[(2-[(adenin-9-yl)methyl]propane-1,3-diyl)bis(oxy)]bis(methylene)}bis(phosphonate)^36^ (**23**, 1.3 g, 2.25 mmol), yield 1.2 g (48%).

^1^H NMR (500.0 MHz, DMSO-*d*_6_): 8.16 (s, 1H, H-2) 8.09 (s, 1H, H-8), 7.25–7.12 (m, 22H, Ph, NH_2_), 4.69–4.59 (m, 2H, PNH), 4.32–4.25 (m, 2H, PNH), 4.19–4.10 (m, 2H, H-1′), 4.06–3.91 (m, 12H, NHCH, Et), 3.25–3.05 (m, 8H, H-3′, H-4′, H-5′, H-6′), 2.94–2.77 (m, 8H, PhCH_2_), 2.26 (m, 1H, H-2′), 1.13–1.03 (m, 12H, Et). ^13^C NMR (126 MHz, DMSO-*d*_6_): 173.07–172.84 (m, **C**OO), 156.22 (C-6), 152.78 (C-2), 149.83 (C-4), 141.64 (C-8), 137.33–137.26 (m, *i*-Ph), 129.61, 129.58 (*o*-Ph), 128.24 (*m*-Ph), 126.64–126.59 (m, *p*-Ph), 118.74 (C-5), 70.56–70.14 (m, C-3′, C-5′), 68.23–67.05 (m, C-4′, C-6′), 60.61–60.49 (m, Et), 54.24–53.98 (m, PNH**C**H), 41.01 (C-1′), 40.0 (Ph**C**H_2_), 39.5 (C-2′), 14.11–14.04 (m, Et). MS (ESI^+^) *m/z* = 1112 [M + H]^+^. HR-MS (ESI^+^) *m/z*: calcd for C_55_H_72_O_12_N_9_P_2_ = 1112.47702 [M + H]^+^, found 1112.47717.

### Tetra-(l-phenylalanine ethyl ester) prodrug of (2-[3-(adenin-9-yl)-2-(2-bis(hydroxyphosphoryl)ethoxy)propoxy]ethyl}phosphonic acid (26)

Prepared by Method G starting from **19** (0.7 g, 1.3 mmol), yield 0.83 g (57%).

^1^H NMR (500.0 MHz, DMSO-*d*_6_): 8.16 , 8.15 (s, 1H, H-2) 7.97, 7.97 (s, 1H, H-8), 7.27–7.09 (m, 22H, Ph, NH_2_), 4.52–4.42 (m, 2H, PNH), 4.21–4.16 (m, 1H, H-1′), 4.11–3.81 (m, 15H, H-1′, PNH, PNHCH, Et), 3.61 (m, H-2′), 3.47–3.23 (m, 4H, C-3′, C-6′), 3.17–3.15 (m, 2H, H-5′), 2.94–2.68 (m, 8H, PhCH_2_), 1.64–1.39 (m, 4H, H-4′, H-7′), 1.13–1.03 (m, 12H, Et). ^13^C NMR (126 MHz, DMSO-*d*_6_): 173.05–173.30 (m, **C**OO), 156.14 (C-6), 152.61 (C-2), 149.90, 149.88 (C-4), 141.58 (C-8), 137.47–137.47 (m, *i*-Ph), 129.60–129.55 (m, *o*-Ph), 128.29–128.26 (m, *m*-Ph), 126.67–126.62 (m, *p*-Ph), 118.61 (C-5), 76.11, 75.96 (C-2′), 69.46, 69.43 (C-3′), 65.62 (C-6′), 64.30, 64.28 (C-3′), 60.56–60.43 (m, Et), 54.40–53.91 (m, PNH**C**H), 43.76, 43.73 (C-1′), 40.07 (Ph**C**H_2_), 30.77–29.54 (m, C-4′, C-7′), 14.13, 14.06 (m, Et). HR-MS (ESI^+^) *m/z*: calcd for C_56_H_74_N_9_O_12_P_2_ = 1126.49267 [M + H]^+^, found 1126.49302.

### Tetra-(ethyl l-phenylalanine) prodrug of 9-[(*N*-phosphonoethyl-*N*-phosphonomethoxyethyl)-2-aminoethyl]adenine (27)

Prepared by Method G starting from **14** (0.43 g, 0.74 mmol), yield 0.41 g (49%).

^1^H NMR (400 MHz, DMSO-*d*_6_) δ 8.15 (s, 1H, H-2), 8.07 (s, 1H, H-8), 7.19 (m, 22H, NH_2_ and Ph), 4.48 (m, 2H, NH) 4.18 (m, 1H, NH), 4.10 (m, 1H, NH), 4.01 (m, 14H, H-1′, Et and CHNH), 3.24 (m, 2H, H-7′), 3.12 (t, *J* = 6.2, H-6′), 2.85 (m, 8H, CH_2_Ph), 2.66, (t, *J* = 6.2, 2H, H-2′), 2.46 (m, 2H, H-3′), 2.38 (t, *J* = 6.2, H-5′), 1.53 (m, 2H, H-4′), 1.34 (m, 2H, H-8′), 1.09 (m, 12H, Et). ^31^P NMR (162 MHz, DMSO-*d*_6_) δ 29.37 and 27.50. ^13^C NMR (101 MHz, DMSO-*d*_6_) δ 173.02 (4C, CO), 155.91 (C-6), 152.30 (C-2), 149.48 (C-4), 141.15 (C-8), 137.22 (4C, Ph), 129.39 (8C, Ph), 128.04 (8C, Ph), 126.47 (4C, Ph), 118.57 (C-5), 68.25 (C-6′), 64.82 (C-7′), 60.27 (4C, Et), 54.06 (4C, CHNH), 52.71, 51.91 and 47.78 (C-2′, C-3′ and C-5′), 40.87 (C-1′), 29.89 (d, *J* = 111.2, C-8′). 25.85 (d, *J* = 111.2, C-4′). HR-MS (ESI^+^) *m/z*: calcd for C_57_H_77_O_11_N_10_P_2_ = 1139.52430 [M + H]^+^, found 1139.52418.

### Hexa-(ethyl l-phenylalanine) prodrug of (2-((2-((2-(adenine-9-yl)ethyl)(2-phosphonoethyl)amino)ethyl)(2-phosphonoethyl)amino)ethyl)phosphonic acid (28)

Prepared by Method G starting from **16** (0.72 g, 1 mmol), yield 0.84 g (53%).

^1^H NMR (400 MHz, DMSO-*d*_6_) δ 8.16 (s, 1H, H-2), 8.08 (s, 1H, H-8), 7.17 (m, 32H, NH_2_ and Ph), 4.51 (m, 3H, NH) 4.26 (m, 3H, NH), 4.01 (m, 16H, H-1′, Et and CHNH), 2.82 (m, 12H, CH_2_Ph), 2.64, (m, 2H, H-2′), 2.48 (m, 4H, CH_2_N), 2.32 (m, 4H, CH_2_N), 2.08 (m, 2H, CH_2_N), 1.36 (m, 6H, H-4′, H-8′ and H-10′), 1.05 (m, 18H, Et). ^31^P NMR (162 MHz, DMSO-*d*_6_) δ 30.02 (2P) and 29.58. ^13^C NMR (101 MHz, DMSO-*d*_6_) δ 173.08 (6C, CO), 155.95 (C-6), 152.41 (C-2), 149.53 (C-4), 141.07 (C-8), 137.19 (6C, Ph), 129.33 (12C, Ph), 127.88 (12C, Ph), 126.45 (6C, Ph), 118.64 (C-5), 60.29 (6C, Et), 54.07 (6C, CHNH), 52.48, 50.64, 48.62, 46.54, 45.86 and 45.61 (C-2′, C-3′, C-5′, C-6′, C-7′ and C-9′), 40.91 (C-1′), 23.98–26.51 (m, C-4′, C-8′ and C-10′). HR-MS (ESI^+^) *m/z*: calcd for C_81_H_109_O_15_N_13_P_3_ = 1596.73735 [M + H]^+^, found 1596.73779.

### Docking calculations

Docking calculations were performed with the program GOLD^46^. Three-dimensional coordinated for the ligands were generated using eLBOW^47^. Coordinates used for the docking were polypeptide chain A of *T. brucei* APRT (PDB code 5VN4). All of the ligands and water molecules were deleted. All of the side chains were protonated according to the standard pK_a_ values. The centre of the active site was defined as the location where the N9 nitrogen atom of adenine is located in the original coordinates. The search radius was 12 Å. Docking scores were calculated using ChemPLP within the GOLD program. ChemPLP is the default algorithm in GOLD for calculating docking scores and is dimensionless. It is calculated based on a hydrogen bonding term and multiple linear potentials to model van der Waals interactions and steric clashes^48^. Images were drawn using PyMOL 2.4^49^.

### Trypanosoma culture and cell lines

The bloodstream form (BSF) *T*. *b*. *brucei* Lister 427 and single marker (SM) strains were cultivated in HMI-11 medium and 10% FBS at 37 °C in a humidified atmosphere at 5% CO_2_^26^. HMI-11 medium containing dialyzed 10% FBS and only one defined source of the purine base, adenine, was used for selected experiments (50 μM, HMI-11^adenine^). The SM cell line constitutively expressing ectopic T7 RNA polymerase and tetracycline repressor was used for the tetracycline-inducible expression of dsRNA and V5-tagged proteins. To generate SKD APRT1 and DKD APRT1/2 RNAi cell lines, the 524 bp and 537 bp fragments of the *aprt-I* (Tb927.7.1780) and and *aprt-II* (Tb927.7.1790) open reading frames, respectively, were PCR amplified from *T*. *brucei* BSF427 genomic DNA with the oligonucleotides (Supplementary File S1). The APRT1 fragment was cloned into the p2T7-TABlue, while the APRT2 fragment was cloned into, into the p2T7-177 plasmid^50^. The p2T7-TABlue plasmid containing the APRT1 fragment was electroporated to SM cells which were subjected to selection with hygromycin (5 µg/ml). Verified SKD APRT1 cell line was then transfected with Not-1 linearized p2T7-177 plasmid containing the APRT2 fragment and the resulting DKD APRT1/2 cell line was selected with phleomycin (2.5 µg/ml). For the inducible expression of V5-tagged APRT1 andAPRT2, the coding sequences of the respective genes were PCR amplified using oligonucleotides listed in Supporting Information S1. The obtained PCR fragments were cloned into the pT7_N-term-V5 vector^6^ using BglII or BamHI and XbaI restriction enzymes. Plasmids were then transfected to SM BSF cells. The resulting transfectants were established in medium containing puromycin (0.1 µg/ml). The induction of the dsRNA or tagged proteins was triggered by the addition of 1 µg/ml of tetracycline into the medium.

### Isolation of total RNA, reverse transcription, and qPCR

Quantitative reverse transcription PCR (RT-qPCR) was performed using a Light Cycler^®^ 480 system (Roche) according to the manufacturer’s recommendations. Briefly, total RNA from 1 × 10^7^ BSF cells was extracted using the miRNeasy Kit (Qiagen, Germany) and 10 μg were treated with TURBO DNAse (Ambion). The total RNA (2 µg) was reverse transcribed using the TaqMan Reverse Transcription kit (ThermoFisherSci). The resulting cDNA (100 ng) was mixed with SYBR Green (Applied Biosystems) and specific primers for either the APRT1 and APRT2 transcripts or the internal control transcripts (18S rDNA and β-tubulin) (Supporting File S1). The samples were subjected to analysis on a Light Cycler 480 (Roche).

### Immunofluorescence assay

Cells in the exponential growth phase were stained with 100 nM red-fluorescent stain MitoTracker Red CMXRos (Thermo Fisher Scientific) for 30 min at 37 °C. After that we followed the protocol published in Ref.^6^. Briefly, 1 × 10^8^ of BSF trypanosomes were washed with phosphate-buffered saline supplemented with 10 g/l glucose (PBS-G), and spread onto slides coated with poly-lysin (100 μg/ml). The cells were fixed with 3.7% formaldehyde in PBS, washed with PBS, and permeabilized with 0.1% Triton X-100 in PBS. The cells were then washed with 1xPBS-T (0.05% Tween). After blocking with 5.5% FBS, the respective slides were incubated for 1 h in PBS-T plus 3% BSA with the following primary monoclonal antibody anti-V5 (1:200, Thermo Fisher Scientific). After washes, the slides were incubated in the dark for 1 h with the following secondary antibodies: goat anti-rabbit IgG (H + L) Texas Red conjugate (1:400, Thermo Fisher Scientific) or goat anti-mouse IgG (H + L) FITC conjugate (1:400, Sigma). The slides were washed and mounted in ProLong Gold Antifade Mountant with DAPI (Thermo Fisher Scientific).

### Expression and purification of *T. brucei* APRT1 and APRT2 in *E. coli*

The *T. brucei aprt1* and *aprt2* genes were PCR amplified using oligonucleotides listed in Supplementary File S1, digested with BglII or BamHI and XhoI enzymes and ligated into the pSKB3 expression vector. The pSKB3 vector carries a 6 × His-tag and an AcTEV cleavage site upstream of BamHI restriction site. A BglII or BamHI restriction site was introduced at the 5′ end of the respective genes instead of the initiation methionine codon to maintain the 6 × His-tag and AcTEV recognition motif in frame with the genes. The verified plasmids were transformed into the BL21(DE3) *E. coli* cells. The 6xhis-tagged recombinant APRT1 protein was purified under native conditions using the ÄKTA prime plus instrument^6^. Elution fractions were analyzed by SDS-PAGE, and those containing APRT1 were pooled and dialyzed against 50 mM Tris–HCl, pH 8.0, 150 mM NaCl, 5 mM MgCl_2_, 10% glycerol. Bradford protein assay (BioRad) was used to determine protein concentrations. The APRT1 recombinant protein was sent for antibody production to Davids Biotechnology (Regensburg, Germany). Recombinant APRT1 was subsequently used in all in vitro assays.

### Alamar Blue assay

An Alamar Blue Assay was performed in a 96-well plate format to screen for sensitivity of *T*. *brucei* BSF cells to ANPs as previously published^6^. The EC_50_ values were calculated using non-linear regression in GraphPad Prism 9.

### Cytotoxicity of the prodrug in human cells

Compound cytotoxicity was evaluated in Hela S3 cancer cells and non-tumor human dermal fibroblasts (NHDF). All cell lines were obtained from ATCC (Manassas, VA, USA). The cytotoxicity was assayed using CellTiter-Glo^®^ 2.0 detection reagent (Promega, Madison, USA) according to the manufacturer’s protocol.

### Protein crosslinking

Purified APRT1 protein was dialyzed to 20 mM HEPES, 150 mM NaCl, 5 mM MgCl_2_, at pH 8.0 and diluted to 0.1 mg/ml and the crosslinking was performed as in Ref.^6^. The crosslinked proteins were fractionated on 15% SDS-PAGE gels and stained with Coomassie Brilliant Blue dye.

### Enzyme activity, determination of kinetic constants and inhibition studies

The methodology was adapted from Ref.^6^. Briefly, the enzyme kinetics of APRT1 were measured by a continuous spectrophotometric assay measuring the conversion of adenine to AMP, at 256 nm. The concentration of enzyme in the assay was 140 nM, the concentration of *P*Rib-*PP* was kept at 1 mM and the adenine concentration varied from 3.75 to 60 μM. The reaction was carried out using a 1.0 cm path length with a UV Visible spectrometer 1601 (Shimadzu) in the reaction buffer (0.1 M Tris, pH 8.4; 0.11 M MgCl_2_; at 25 °C). The initial velocity (V_0_ [µM/min]) was calculated using Beer–Lambert law for different concentration of the purine substrate molecules: A = Δε × L × c where A is the measured absorbance, Δε is constant 2100 M^−1^ cm^−1^ for adenine, c is a concentration of the product formed in one minute (V_0_) and L is the length of a cuvette. The K_m_, V_max_ and k_cat_ values were calculated using GraphPad Prism 9 according to Michaelis–Menten kinetics. The *K*_i_ values of the inhibitors for APRT1 were determined in the same reaction buffer containing 1 mM *P*Rib-*PP*, 60 μM adenine and 280 nM APRT1. The concentrations of inhibitors in the assay ranged from 1 nM to 100 μM, depending on the activity of the compound. The reaction was preceded by 1 min incubation of the respective enzyme with the given inhibitor. The *K*_i_ values were calculated using non-linear regression in GraphPad Prism 9.

### SDS-PAGE and Western blot

Protein samples were separated on SDS-PAGE, blotted onto a PVDF membrane (Thermo Fisher Scientific) and probed with the appropriate monoclonal (mAb anti-V5 epitope tag (1:2000, Thermo Fisher Scientific), mAb anti-mtHsp70 (1:2000^51^), or polyclonal (pAb anti-APRT1 (1:250, this work). This was followed by incubation with a secondary HRP-conjugated anti-rabbit or anti-mouse antibody (1:2000, BioRad). Proteins were visualized on a ChemiDoc instrument using the Clarity Western ECL Substrate. The images were analyzed using ImageLab software version 4.1 (Bio-Rad).

### Treatment with ANPs and cell cycle analysis by flow cytometry

BFS trypanosomes in log phase were resuspended in HMI-11 medium to a density of 2 × 10^5^ cells ml^−1^. Test compounds at concentrations ranging from 1 to 16 µM were added to the cell suspension followed by 12 h incubation at 37 °C. The cell cycle analysis was performed as described previously^52^. Briefly, approximately 2 × 10^6^ cells were washed with PBS and fixed in 1 ml of 70% methanol in 1 × PBS and stored at 4 °C overnight. Following a PBS wash, samples were incubated with 10 µg/ml propidium iodide and 9.6 µg/ml of RNaseA at 37 °C for 45 min. Samples were analyzed on a FACS Canto II (BD) collecting 10,000 gated events.

## Supplementary Information

Supplementary Information.
