# Supplementary material for: Acyclic nucleoside phosphonates with adenine nucleobase inhibit Trypanosoma brucei adenine phosphoribosyltransferase in vitro
Source: Sci Rep. 2021 Jun 25;11:13317. doi: 10.1038/s41598-021-91747-6 (PMC8233378; doi:10.1038/s41598-021-91747-6)
Supplement: Supplementary file 1 — Supplementary Information. [file 41598_2021_91747_MOESM1_ESM.pdf]

Supporting Information
for

Acyclic nucleoside phosphonates with adenine nucleobase inhibit *Trypanosoma brucei* adenine phosphoribosyltransferase *in vitro*

Eva Doleželová,[1] Tomáš Klejch,[2] Petr Špaček,[2] Martina Slapničková, [1] Luke Guddat,[3] Dana Hocková,[2]* and Alena Zíková,[1,4]*

File S1. List of oligonucleotides used in this study

File S2. Spectra of the final compounds

File S3. Raw Images. Original images supporting blot results reported in Figs. 2A, 3 and 4C.

Supplementary file S1. List of oligonucleotides used in this study.

APRT-I p2T7-TA Blue RNAi forward primer: 5'- ATGTCACTTGTGGAGGTTTTG-3'
APRT-I p2T7- TA Blue RNAi reverse primer: 5'- ATACCCGCCACTTCCACAAG-3'.
APRT-II p2T7-177 RNAi forward primer: 5'- TCACAGTATGATGCGATTCTGA-3'
APRT-II p2T7-177 RNAi reverse primer: 5'- GGCCACATCACAGACGGTAA-3'.
APRT-I pT7-V5 N-term forward primer: 5'-CACAGATCTTCACTTGTGGAGGTTTTG -3'
APRT-I pT7-V5 N-term reverse primer: 5'- CACTCTAGATTAAATCAATTTTTTTGCCTCG -3'
APRT-II pT7-V5 N-term forward primer: 5'-CACGGATCCTCACAGTATGATGCGATTCTGA -3'
APRT-II pT7-V5 N-term reverse primer: 5'-CACTCTAGATCACAGTCGTGACCTGGTAATG -3'
APRT-I pSKB3 overexpression forward primer: 5'- CACAGATCTTCACTTGTGGAGGTTTTG -3'
APRT-I pSKB3 overexpression reverse primer: 5'- CACCTCGAGTTAAATCAATTTTTTTGCCTCG -3'

Supplementary file S2. Spectra of the final compounds.

NMR Spectra and HRMS of tested compounds

3a

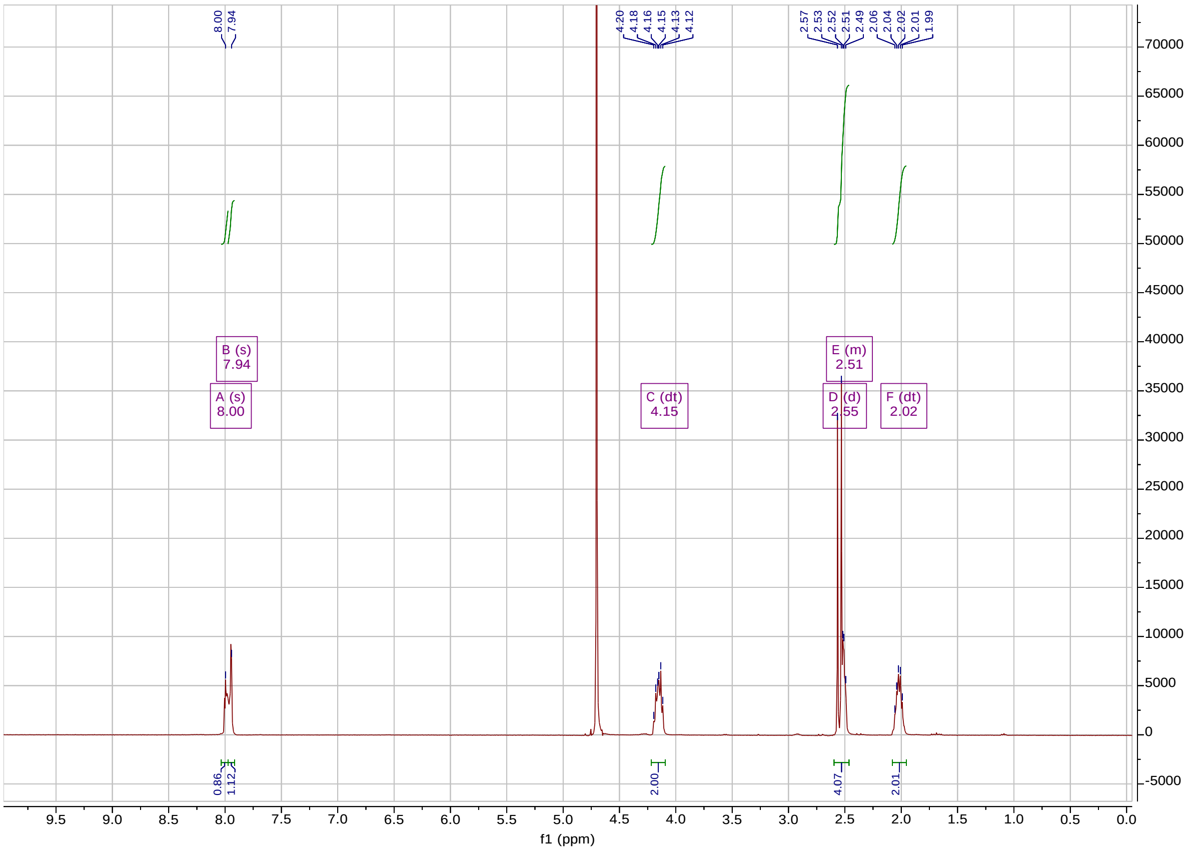

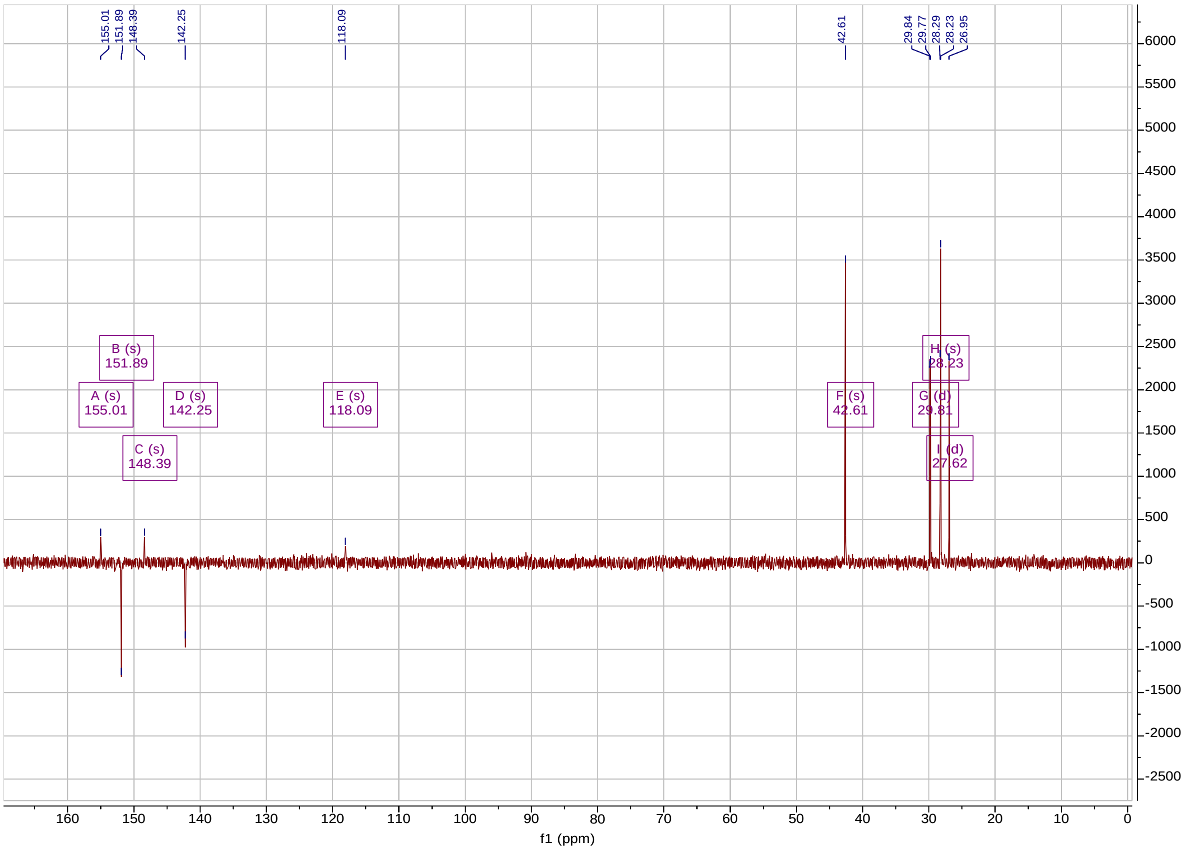

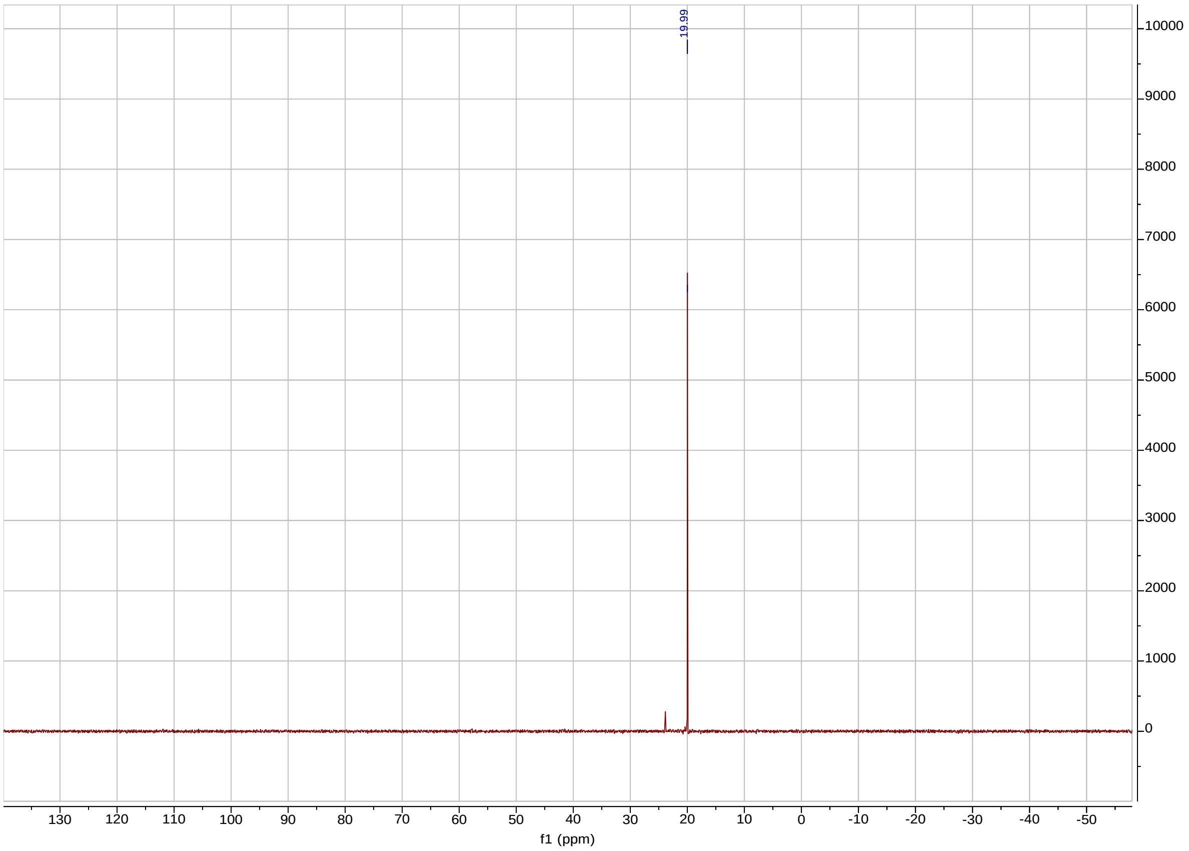

X:\SERVISHR\duben_2019\040419servisHR_33 04/04/19 17:31:31 Klejch, TK371

040419servisHR_33 #30-31 RT: 1.68-1.74 AV: 2 SB: 9 0.10-0.45 , 0.27-0.33 NL: 2.76E6
T: FTMS - p ESI Full ms [220.00-2000.00]

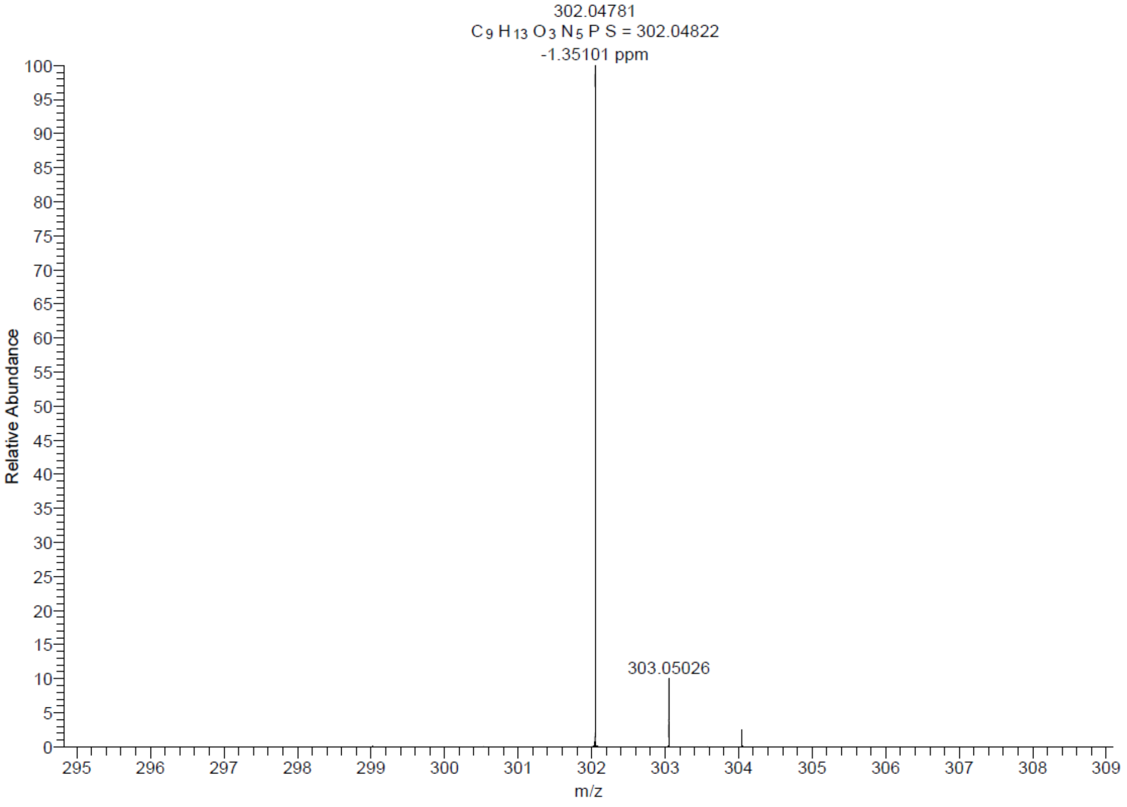

3b

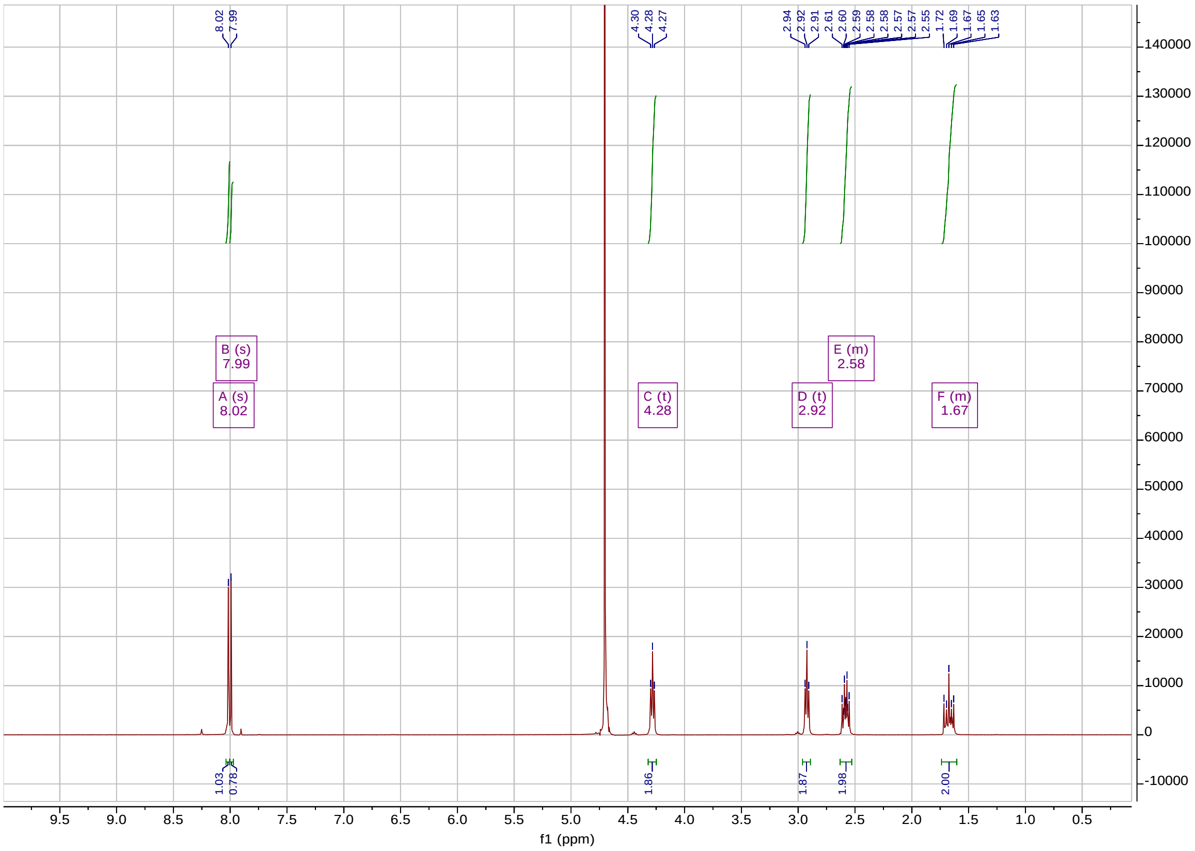
8.02
7.99
4.30
4.28
4.27
2.94
2.92
2.91
2.61
2.60
2.59
2.58
2.58
2.57
2.57
2.55
1.72
1.69
1.67
1.65
1.63
B (s)
7.99
A (s)
8.02
C (t)
4.28
E (m)
2.58
D (t)
2.92
F (m)
1.67
1.03
0.78
1.86
1.87
1.98
2.00
f1 (ppm)

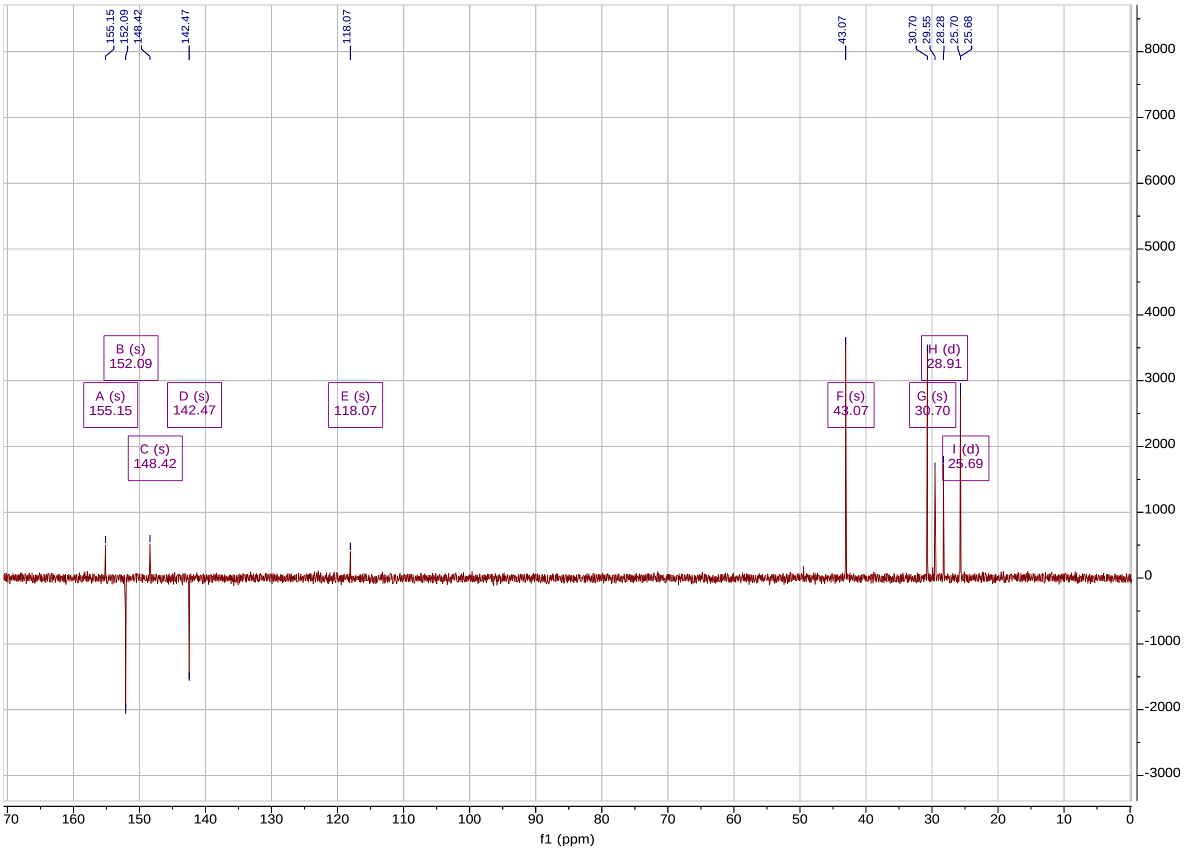
155.15
152.09
148.42
142.47
118.07
43.07
30.70
29.55
28.28
25.70
25.68
B (s)
152.09
A (s)
155.15
D (s)
142.47
C (s)
148.42
E (s)
118.07
F (s)
43.07
H (d)
28.91
G (s)
30.70
I (d)
25.69
f1 (ppm)

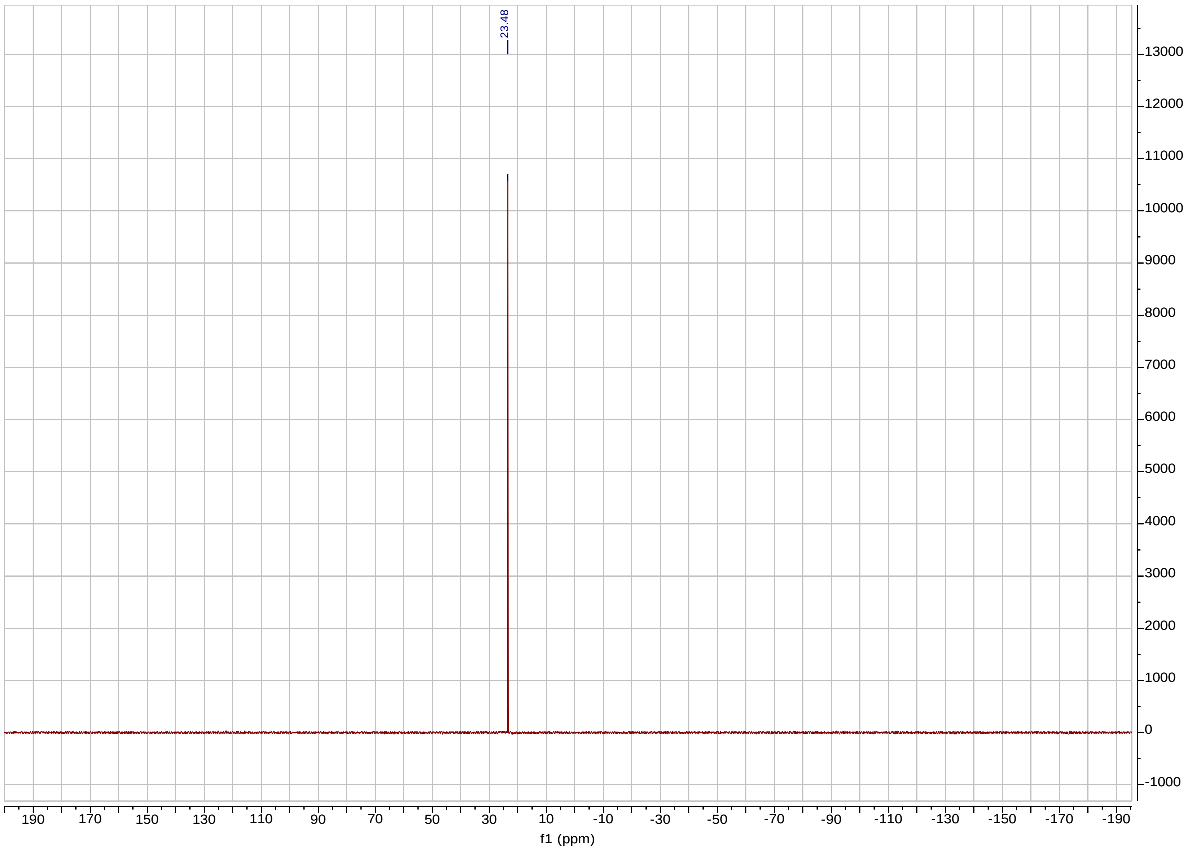

X:\SERVISHR\duben_2019\040419servisHR_32 04/04/19 17:27:27 Klejch, TK370

040419servisHR_32 #28-29 RT: 1.56-1.62 AV: 2 SB: 14 0.04-0.57 , 0.10-0.27 NL: 2.13E5
T: FTMS - p ESI Full ms [220.00-2000.00]

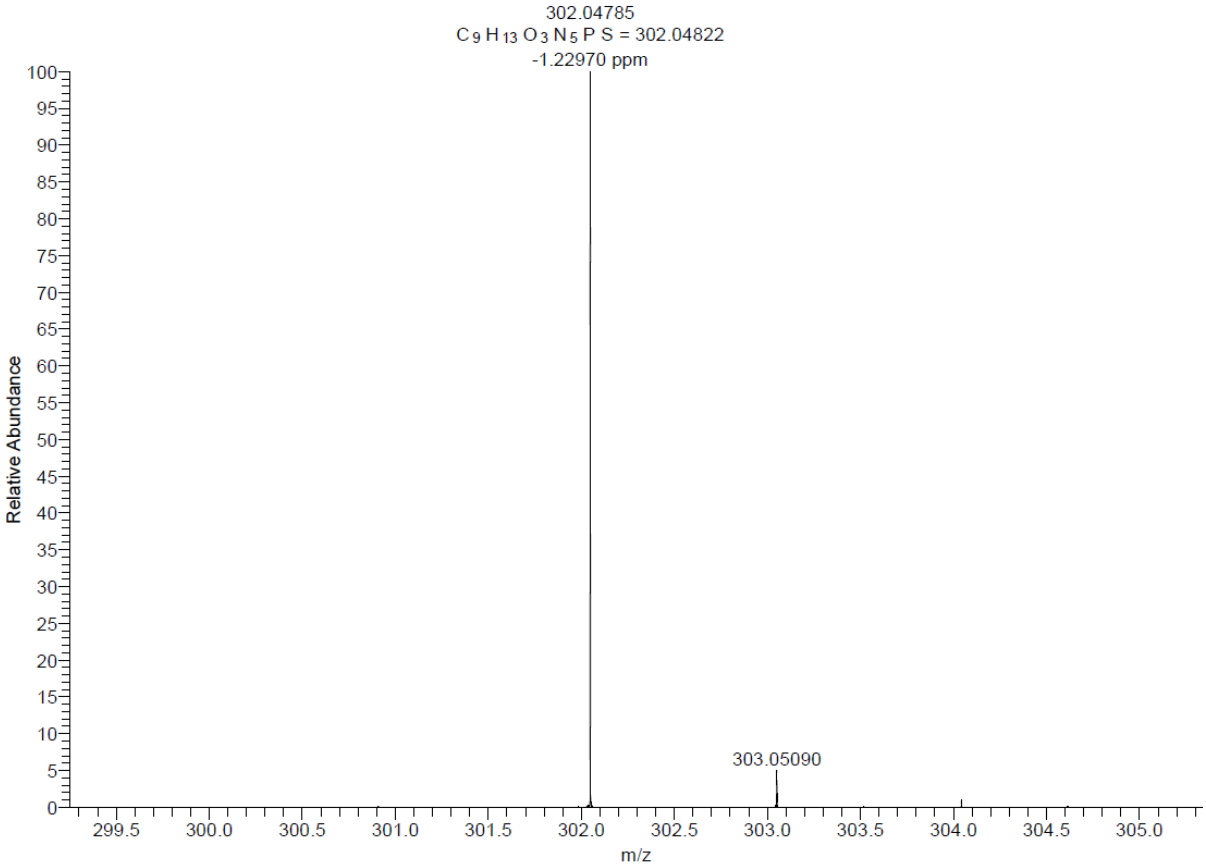

4a

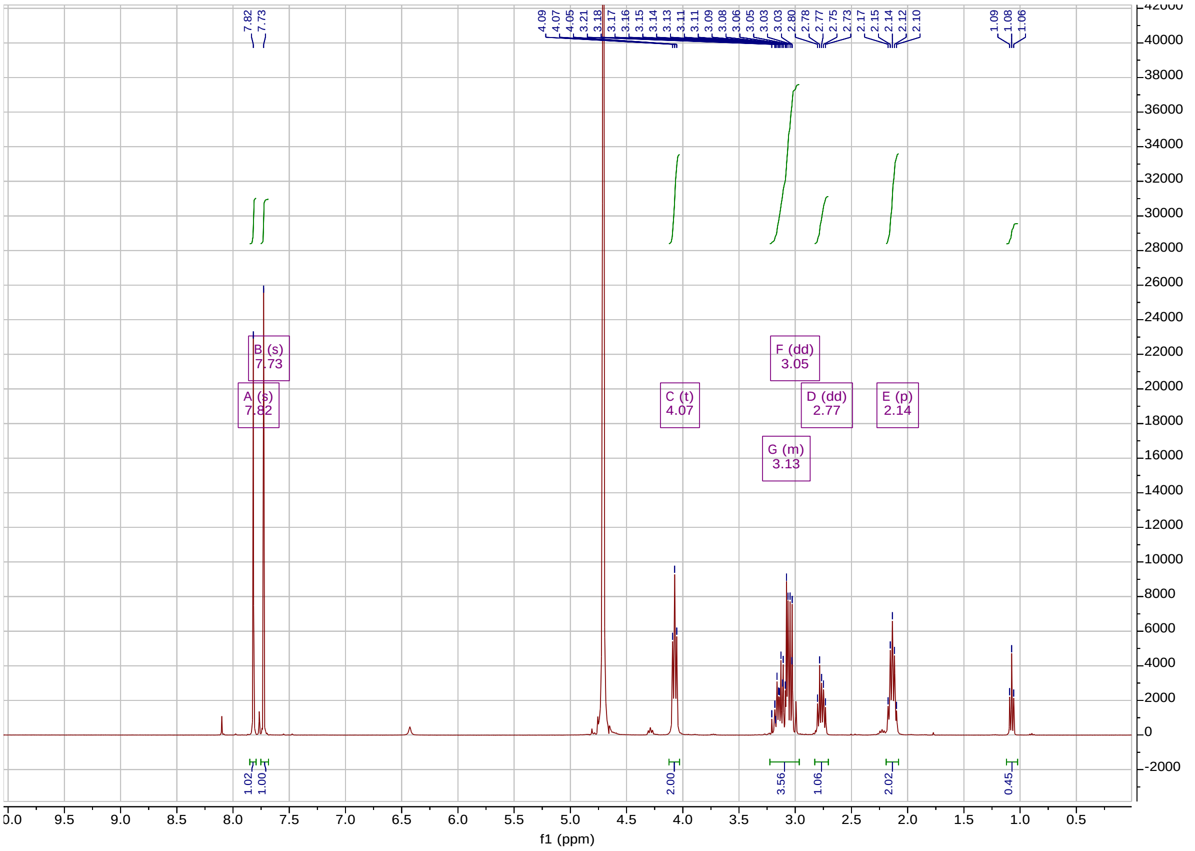

7.82
7.73
4.09
4.07
4.05
3.21
3.18
3.17
3.16
3.15
3.14
3.13
3.11
3.11
3.09
3.08
3.06
3.05
3.03
3.03
2.80
2.78
2.77
2.75
2.73
2.17
2.15
2.14
2.12
2.10
1.09
1.08
1.06
B (s) 7.73
A (s) 7.82
C (t) 4.07
F (dd) 3.05
D (dd) 2.77
E (p) 2.14
G (m) 3.13
1.02
1.00
2.00
3.56
1.06
2.02
0.45
f1 (ppm)

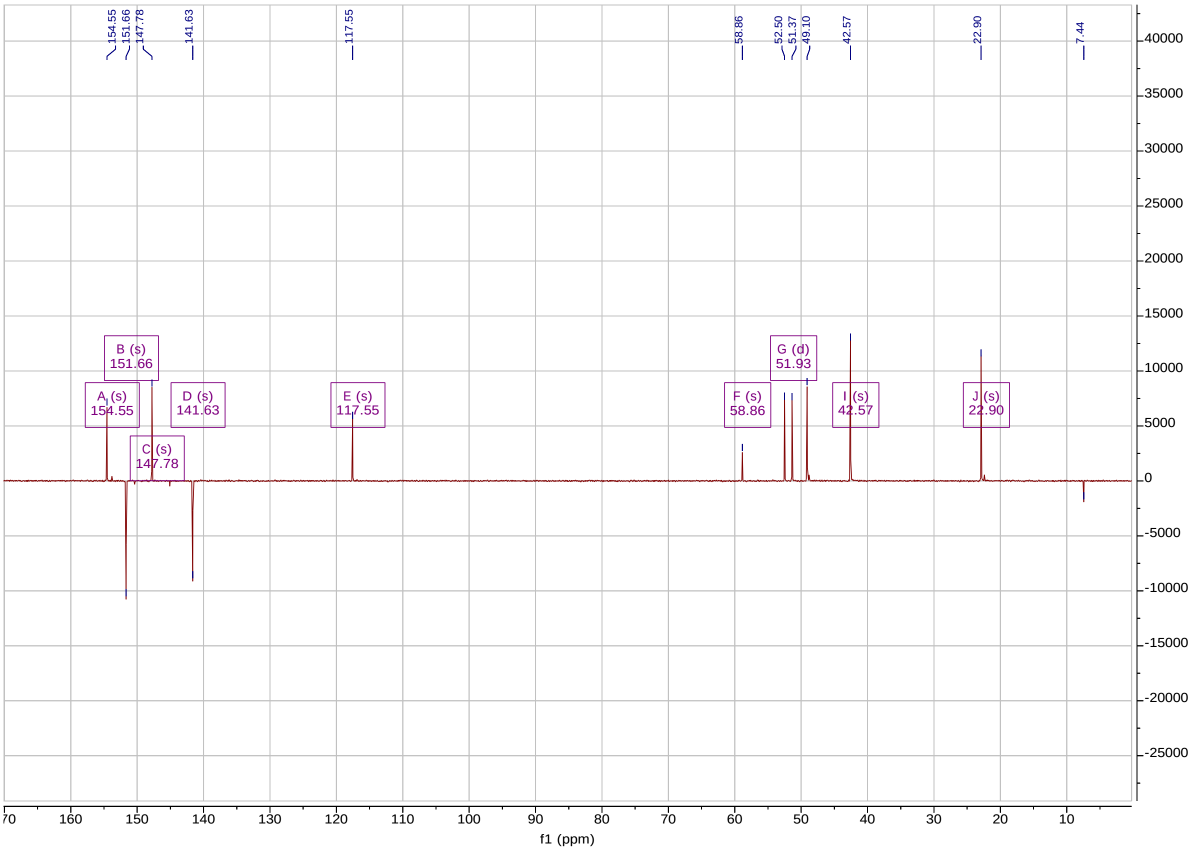

154.55
151.66
147.78
141.63
117.55
58.86
52.50
51.37
49.10
42.57
22.90
7.44
B (s) 151.66
A (s) 154.55
D (s) 141.63
C (s) 147.78
E (s) 117.55
G (d) 51.93
F (s) 58.86
I (s) 42.57
J (s) 22.90
f1 (ppm)

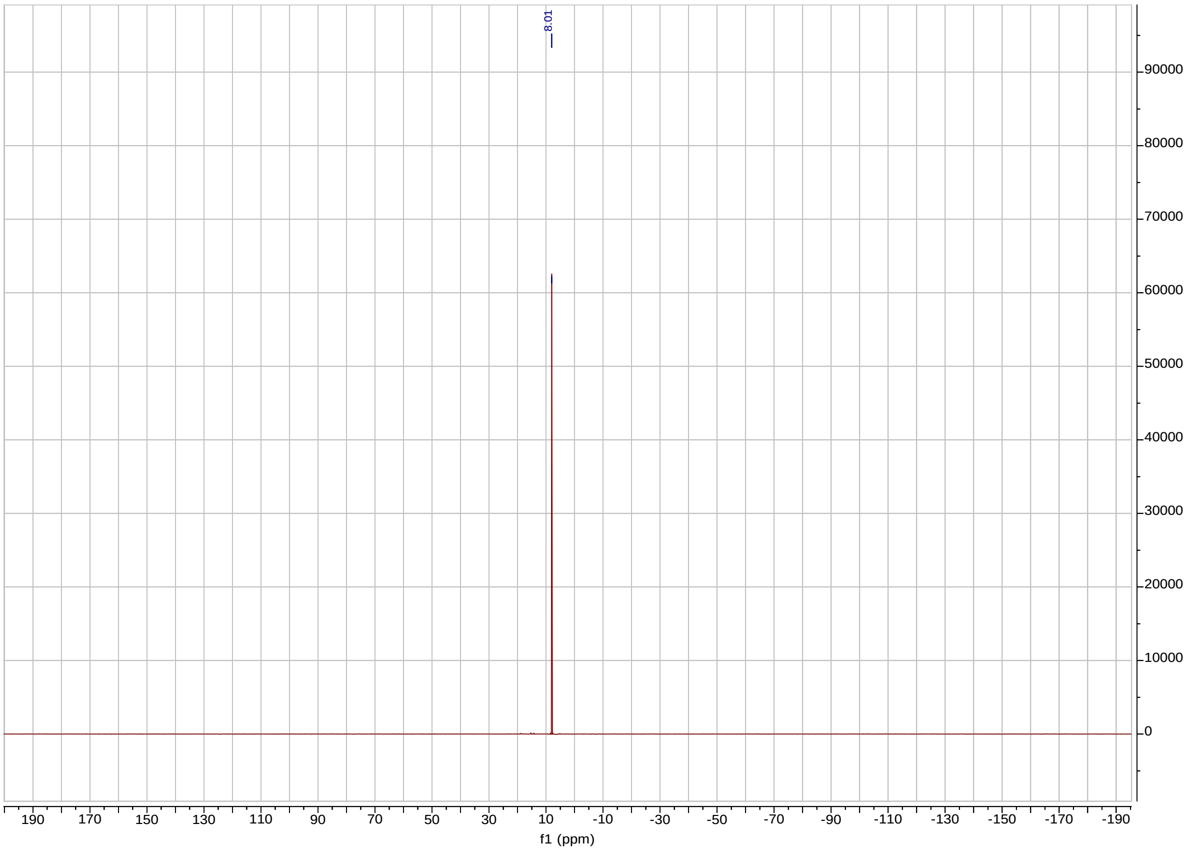

8.01
90000
80000
70000
60000
50000
40000
30000
20000
10000
0
190 170 150 130 110 90 70 50 30 10 -10 -30 -50 -70 -90 -110 -130 -150 -170 -190
f1 (ppm)

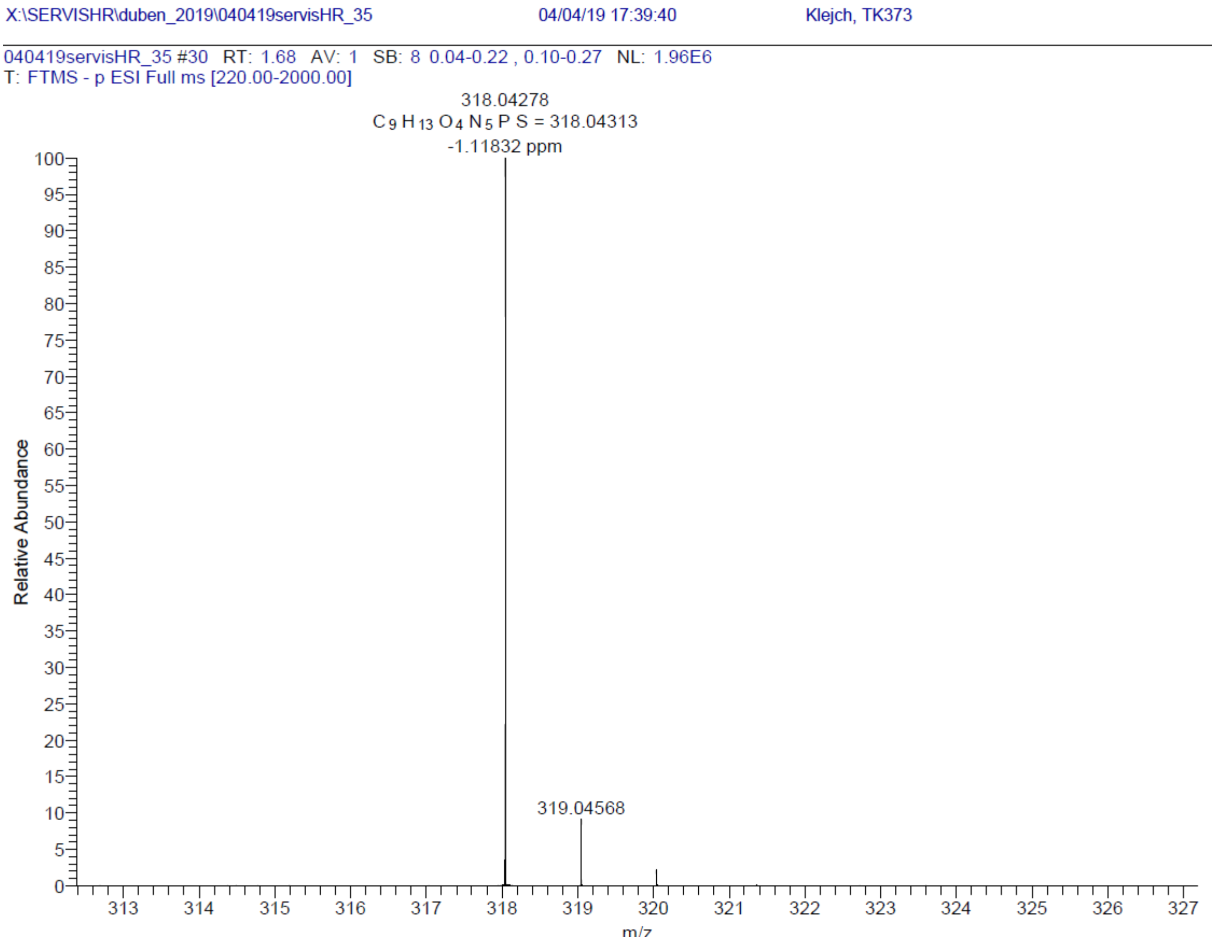

X:\SERVISHR\duben_2019\040419servisHR_35
04/04/19 17:39:40
Klejch, TK373
040419servisHR_35 #30 RT: 1.68 AV: 1 SB: 8 0.04-0.22 , 0.10-0.27 NL: 1.96E6
T: FTMS - p ESI Full ms [220.00-2000.00]
318.04278
C 9 H 13 O 4 N 5 P S = 318.04313
-1.11832 ppm
319.04568
Relative Abundance
m/z

4b

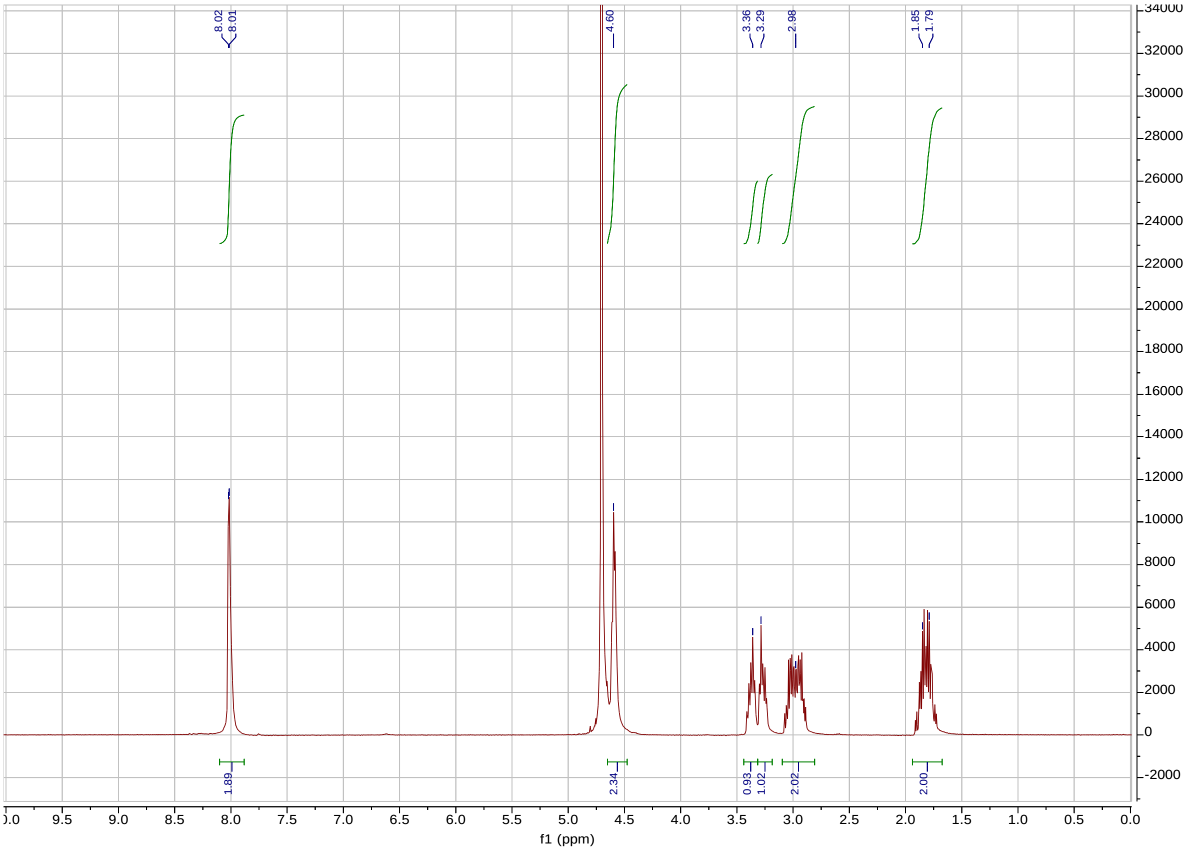

8.02
8.01
4.60
3.36
3.29
2.98
1.85
1.79
1.89
2.34
0.93
1.02
2.02
2.00
f1 (ppm)

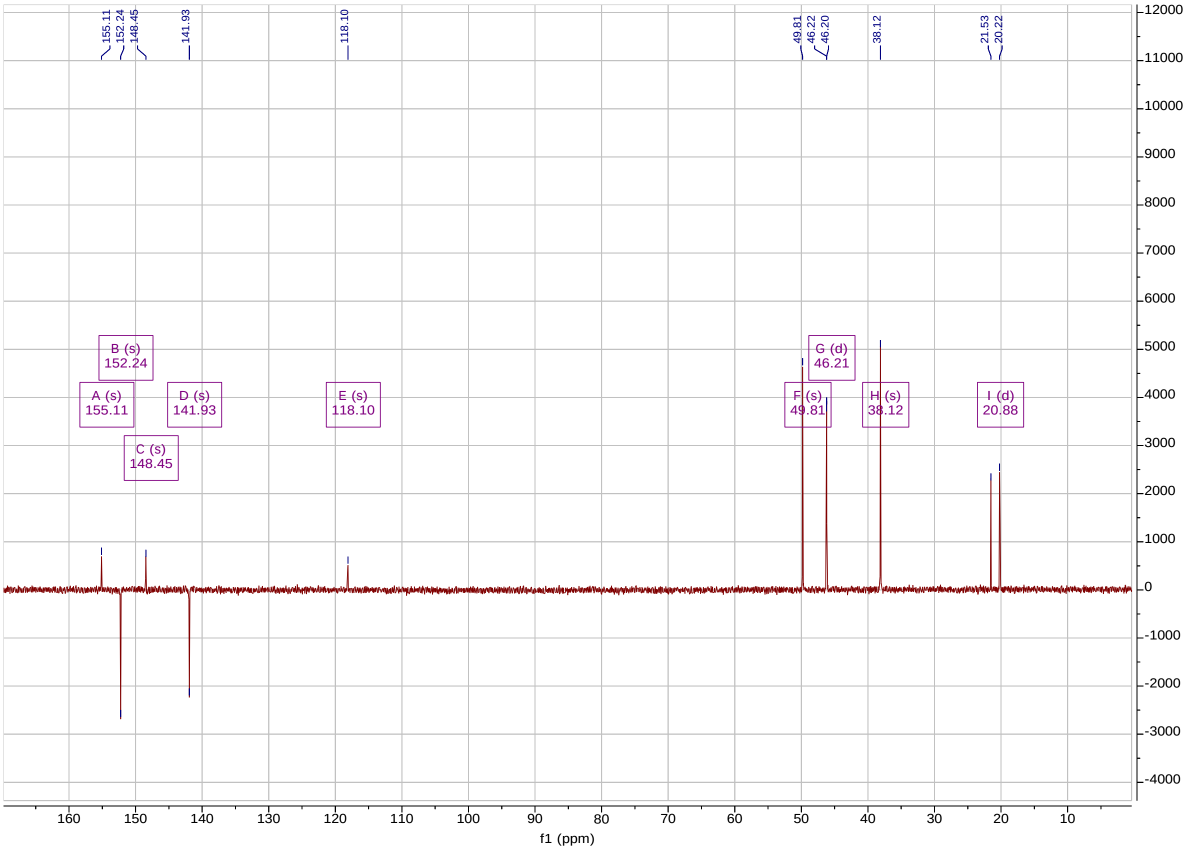

155.11
152.24
148.45
141.93
118.10
49.81
46.22
46.20
38.12
21.53
20.22
A (s) 155.11
B (s) 152.24
C (s) 148.45
D (s) 141.93
E (s) 118.10
F (s) 49.81
G (d) 46.21
H (s) 38.12
I (d) 20.88
f1 (ppm)

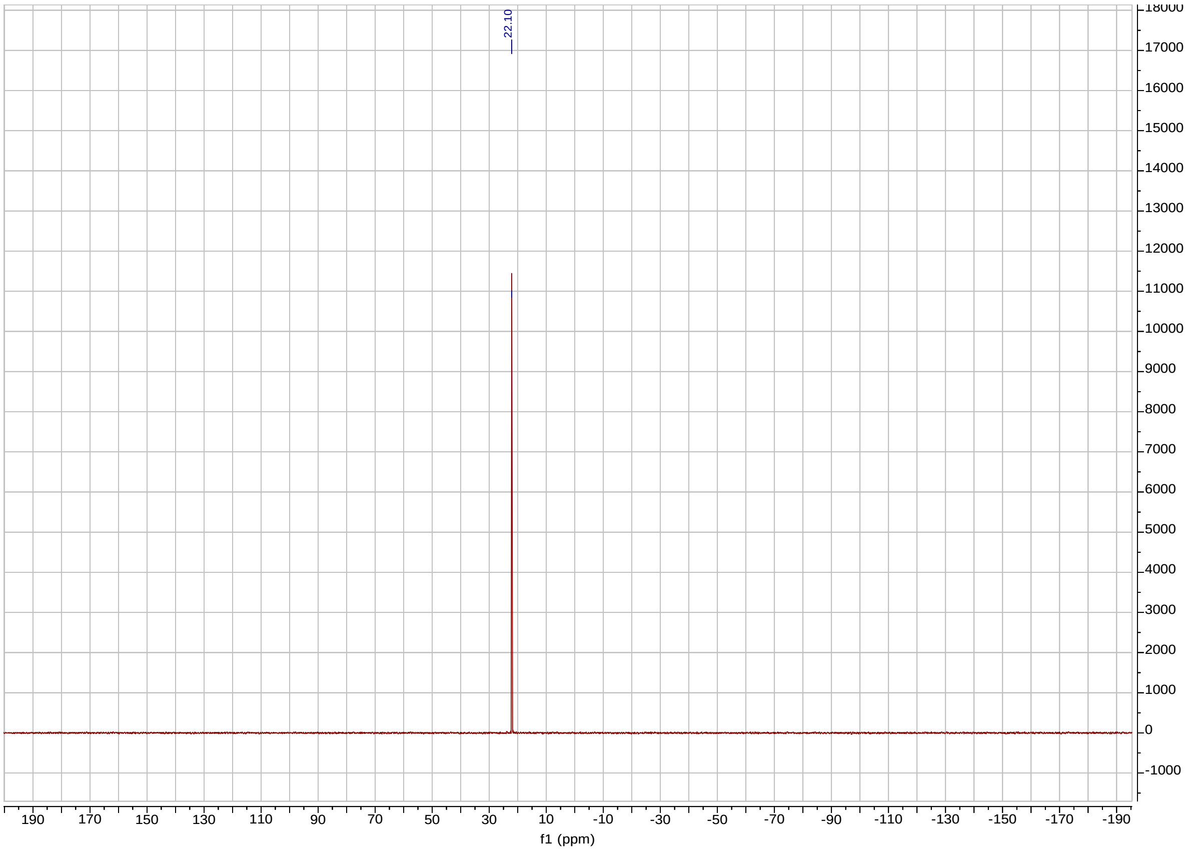

X:\SERVISHR\duben_2019\040419servisHR_34 04/04/19 17:35:37 Klejch, TK372

040419servisHR_34 #29 RT: 1.62 AV: 1 SB: 17 0.04-0.22 , 0.10-0.80 NL: 2.69E6
T: FTMS - p ESI Full ms [220.00-2000.00]

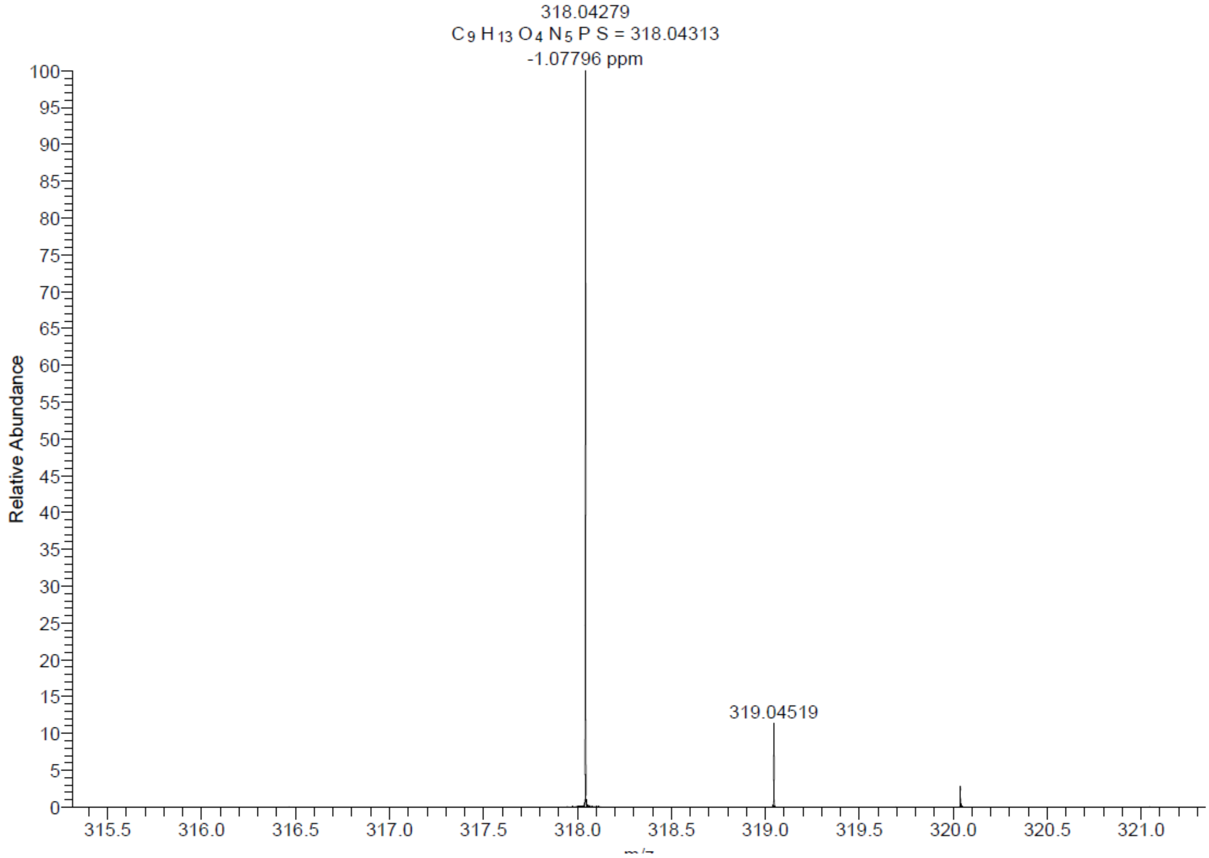

9a

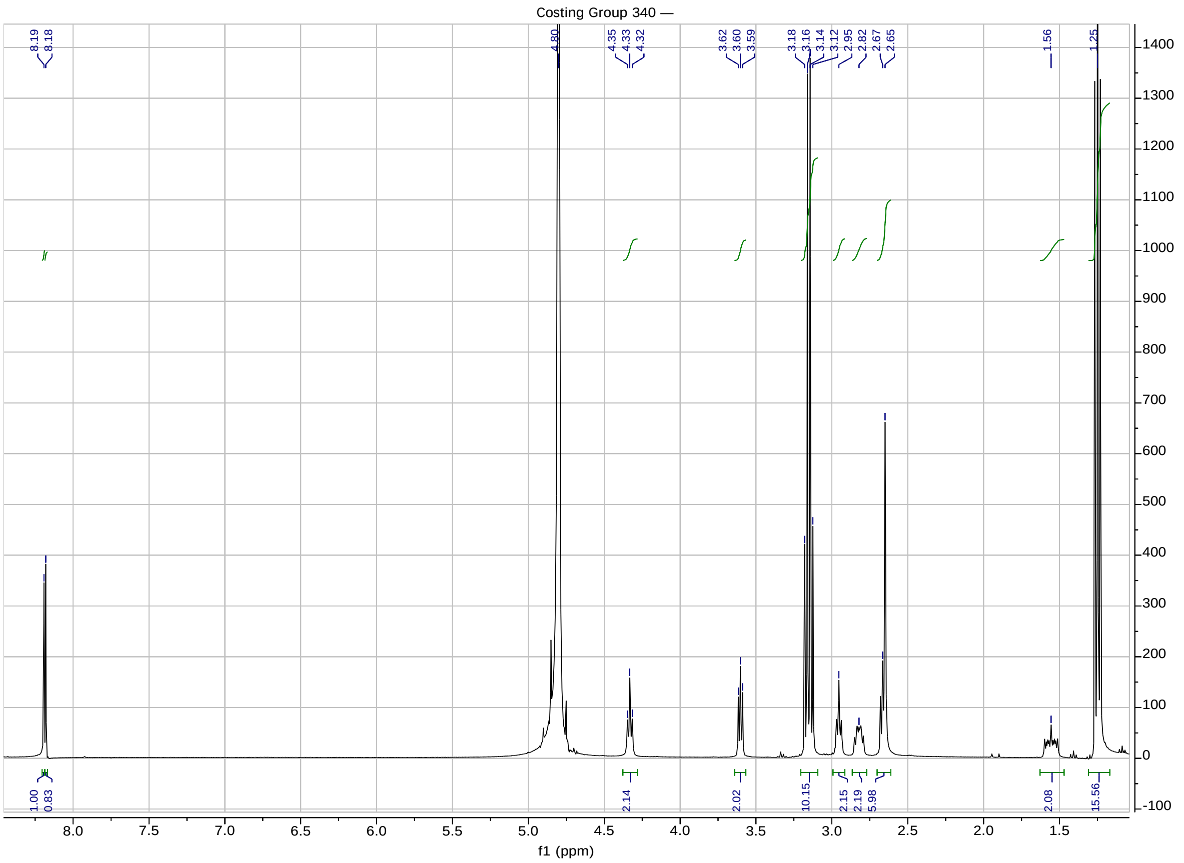
Costing Group 340 —
8.19
8.18
4.80
4.35
4.33
4.32
3.62
3.60
3.59
3.18
3.16
3.14
3.12
2.95
2.82
2.67
2.65
1.56
1.25
1.00
0.83
2.14
2.02
10.15
2.15
2.19
5.98
2.08
15.56
f1 (ppm)

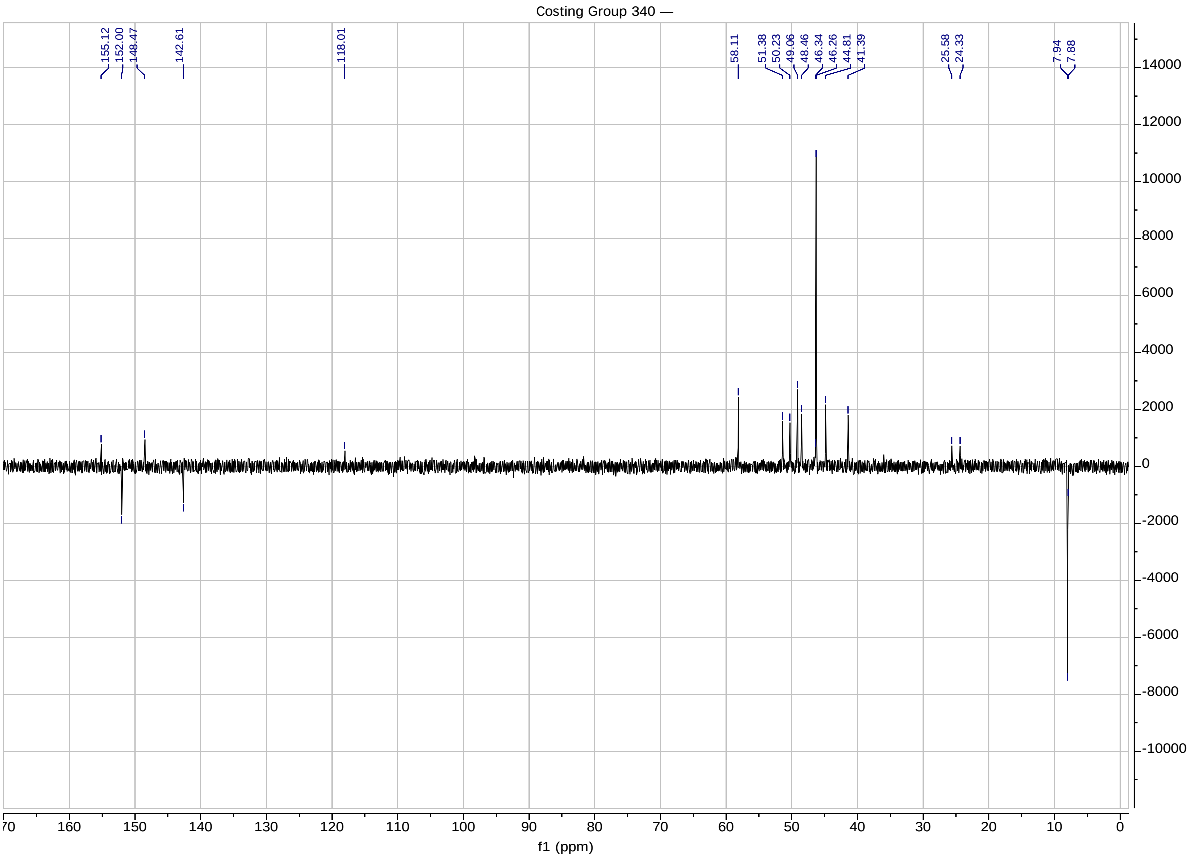
Costing Group 340 —
155.12
152.00
148.47
142.61
118.01
58.11
51.38
50.23
49.06
48.46
46.34
46.26
44.81
41.39
25.58
24.33
7.94
7.88
f1 (ppm)

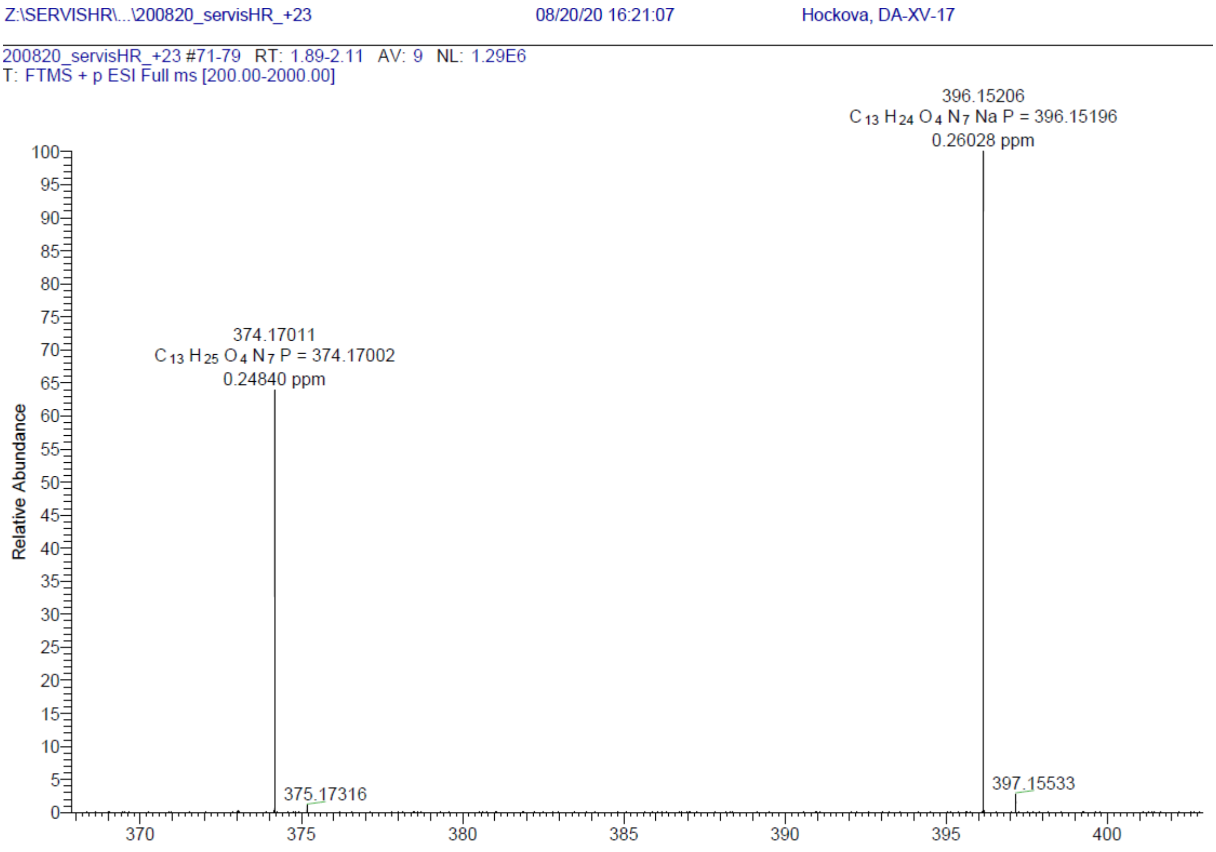

Z:\SERVISHR\...\200820_servisHR_+23
08/20/20 16:21:07
Hockova, DA-XV-17
200820_servisHR_+23 #71-79 RT: 1.89-2.11 AV: 9 NL: 1.29E6
T: FTMS + p ESI Full ms [200.00-2000.00]
396.15206
C 13 H 24 O 4 N 7 Na P = 396.15196
0.26028 ppm
374.17011
C 13 H 25 O 4 N 7 P = 374.17002
0.24840 ppm
375.17316
397.15533
Relative Abundance

9b

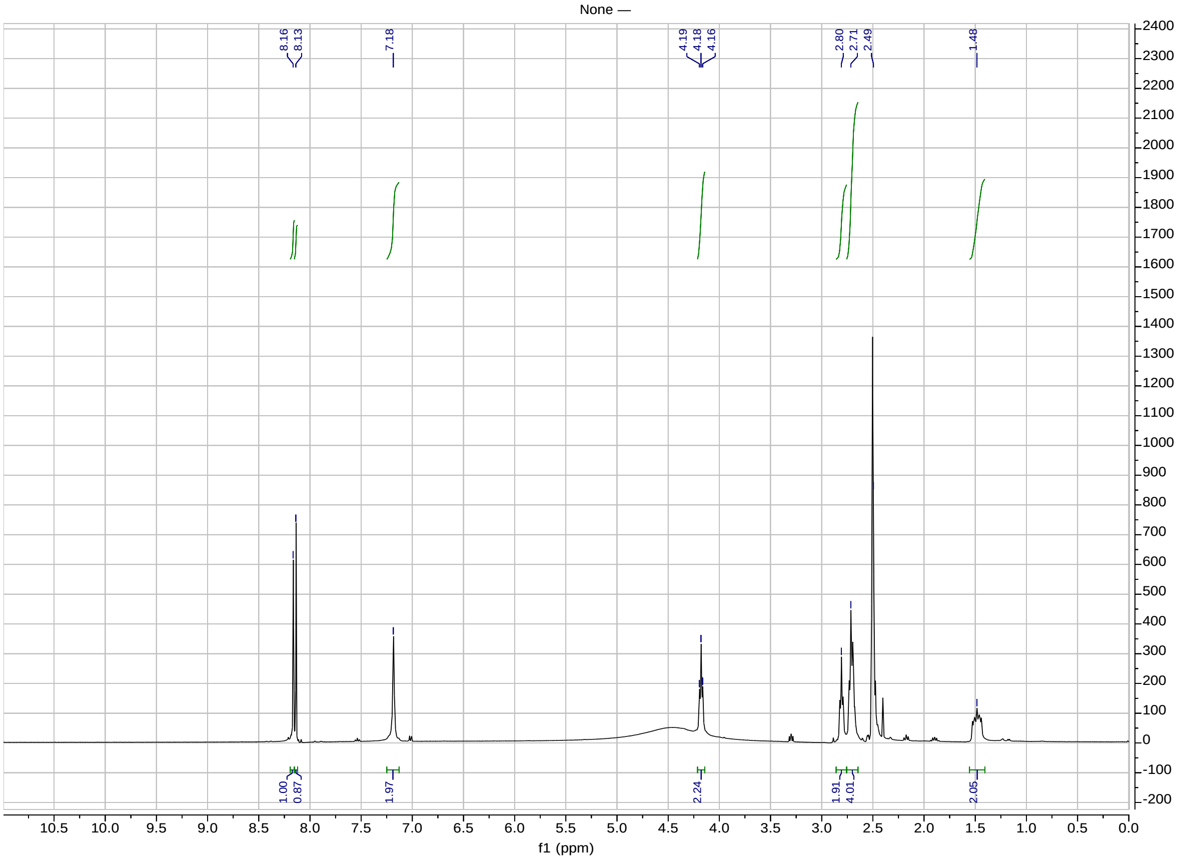

None —
8.16
8.13
7.18
4.19
4.18
4.16
2.80
2.71
2.49
1.48
1.00
0.87
1.97
2.24
1.91
4.01
2.05
f1 (ppm)

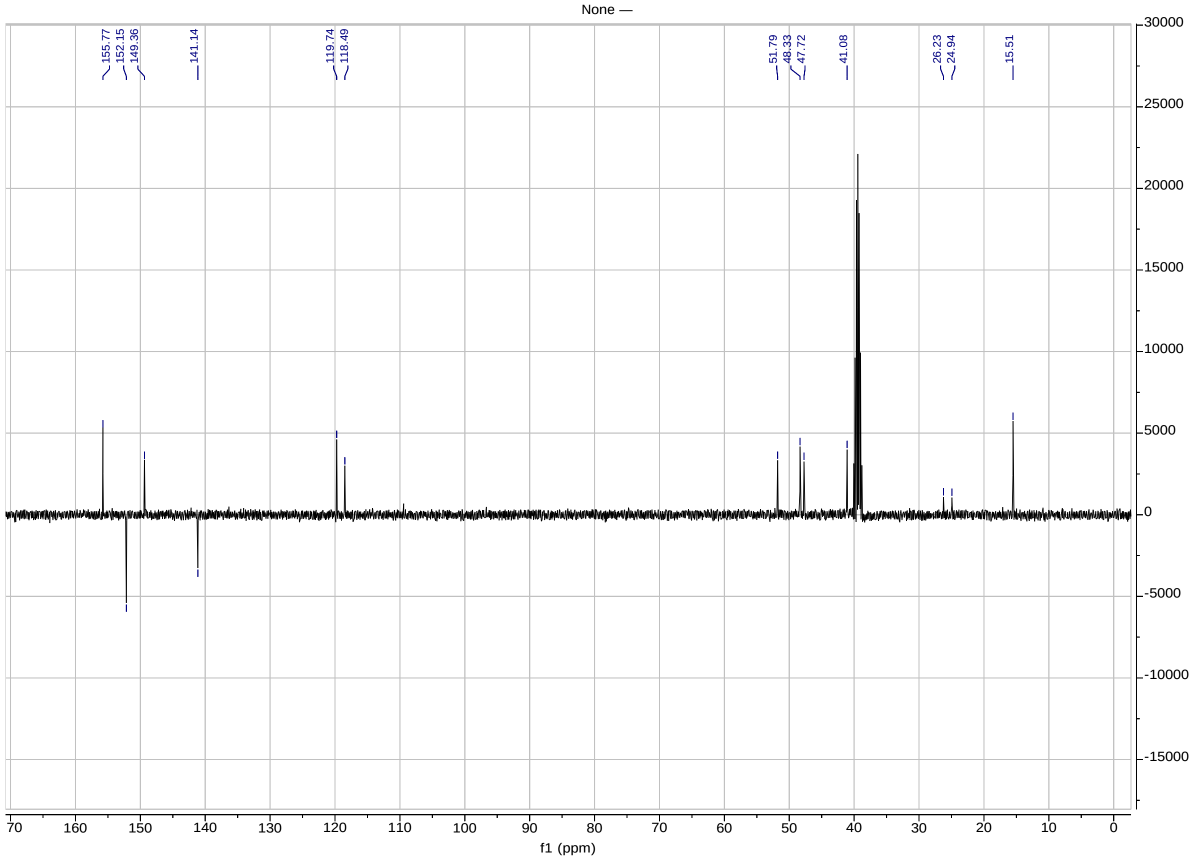
None —
155.77
152.15
149.36
141.14
119.74
118.49
51.79
48.33
47.72
41.08
26.23
24.94
15.51
f1 (ppm)

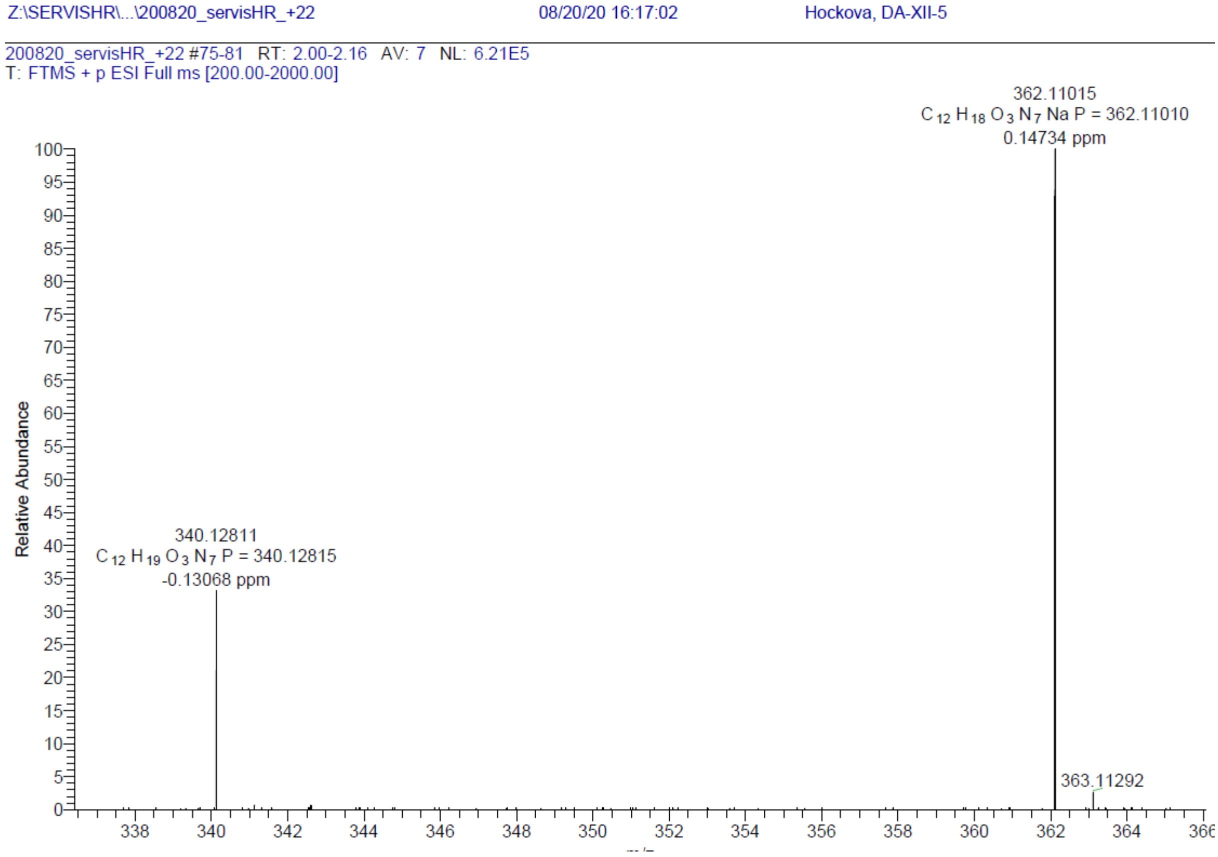
Z:\SERVISHR\...\200820_servisHR_+22
08/20/20 16:17:02
Hockova, DA-XII-5
200820_servisHR_+22 #75-81 RT: 2.00-2.16 AV: 7 NL: 6.21E5
T: FTMS + p ESI Full ms [200.00-2000.00]
362.11015
C 12 H 18 O 3 N 7 Na P = 362.11010
0.14734 ppm
340.12811
C 12 H 19 O 3 N 7 P = 340.12815
-0.13068 ppm
363.11292
Relative Abundance

9c

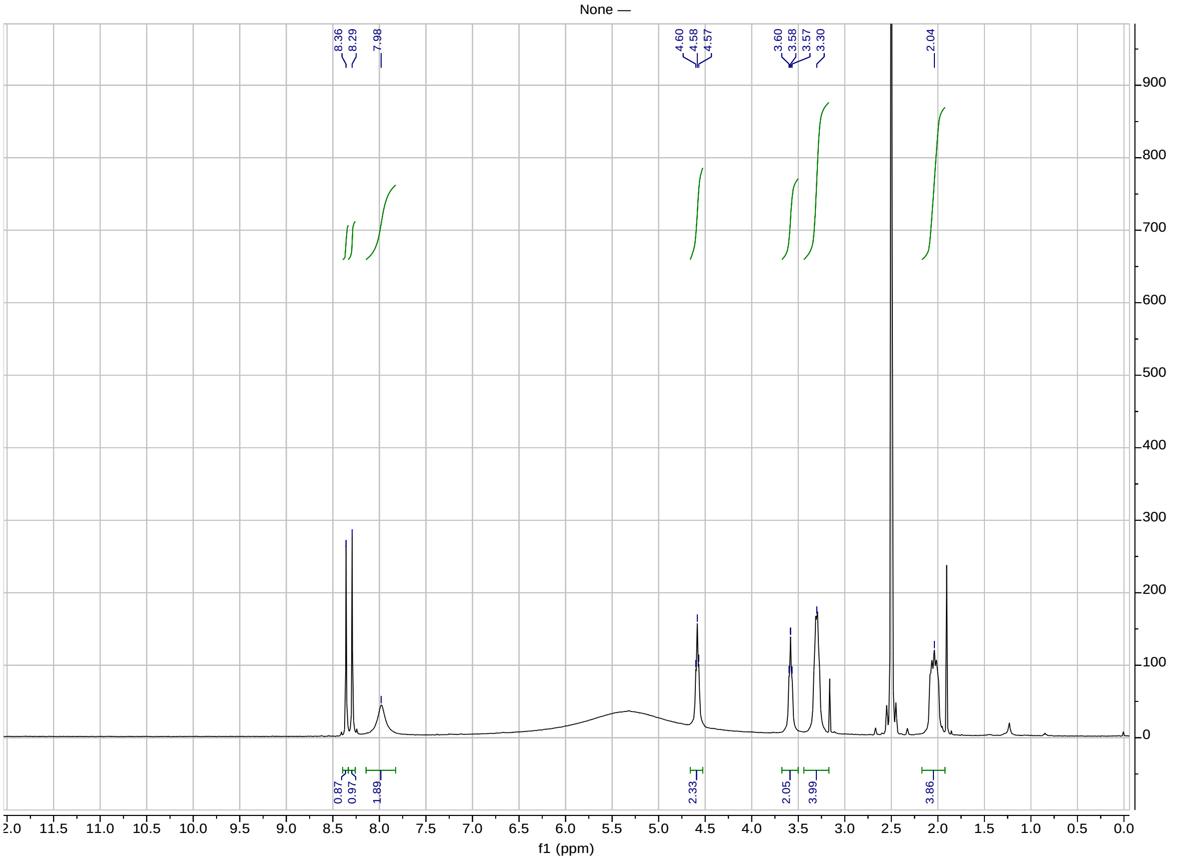
None —
8.36
8.29
7.98
4.60
4.58
4.57
3.60
3.58
3.57
3.30
2.04
0.87
0.97
1.89
2.33
2.05
3.99
3.86
f1 (ppm)

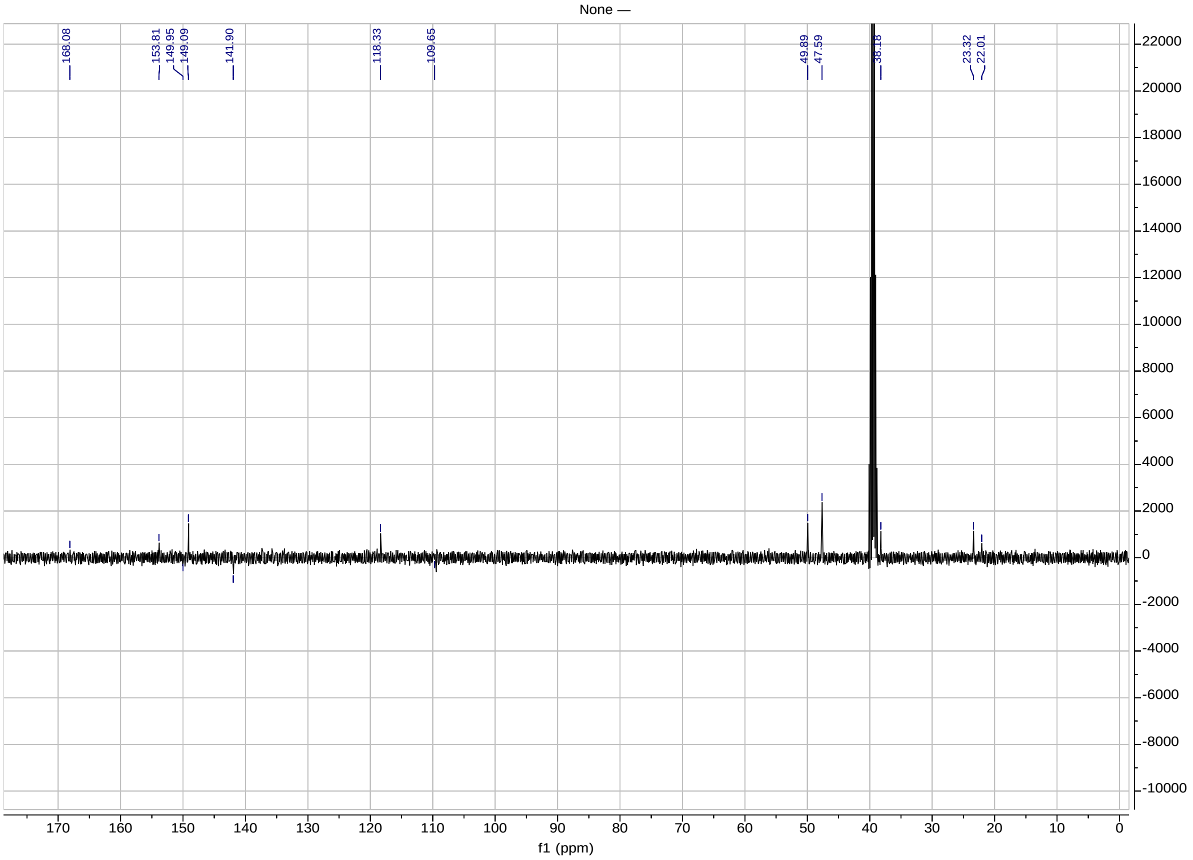
None —
168.08
153.81
149.95
149.09
141.90
118.33
109.65
49.89
47.59
38.18
23.32
22.01
f1 (ppm)

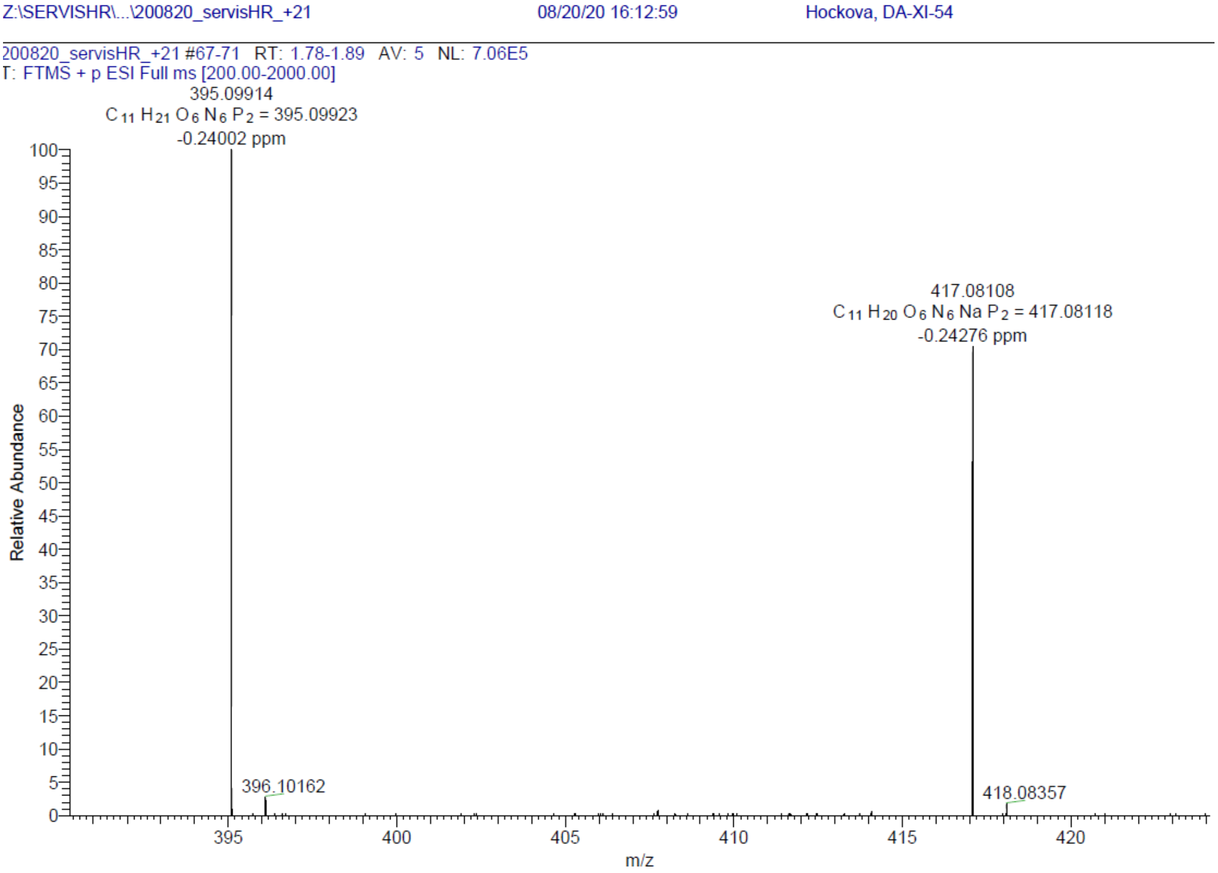
Z:\SERVISHR\...\200820_servisHR_+21
08/20/20 16:12:59
Hockova, DA-XI-54
200820_servisHR_+21 #67-71 RT: 1.78-1.89 AV: 5 NL: 7.06E5
T: FTMS + p ESI Full ms [200.00-2000.00]
395.09914
C 11 H 21 O 6 N 6 P 2 = 395.09923
-0.24002 ppm
417.08108
C 11 H 20 O 6 N 6 Na P 2 = 417.08118
-0.24276 ppm
396.10162
418.08357
Relative Abundance
m/z

9d

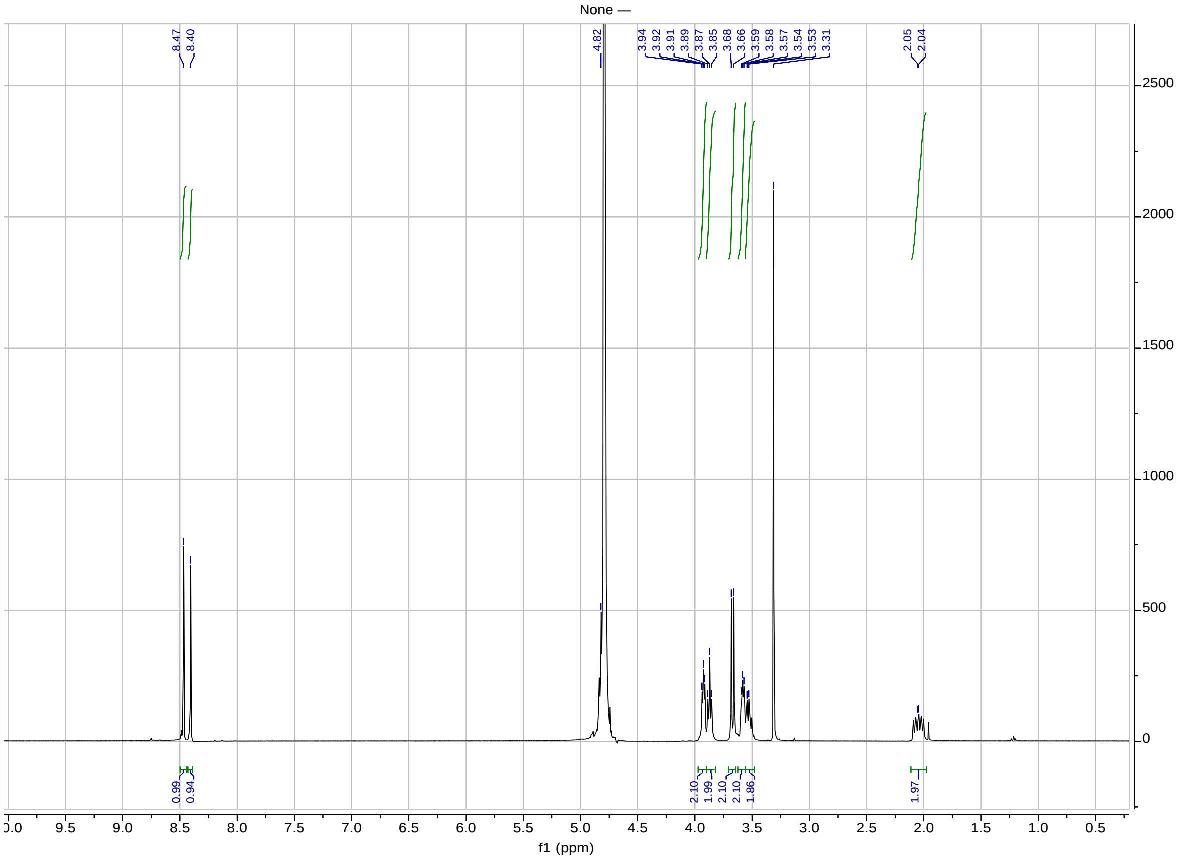
None —
8.47
8.40
4.82
3.94
3.92
3.91
3.89
3.87
3.85
3.68
3.66
3.59
3.58
3.57
3.54
3.53
3.31
2.05
2.04
0.99
0.94
2.10
1.99
2.10
1.86
1.97
f1 (ppm)

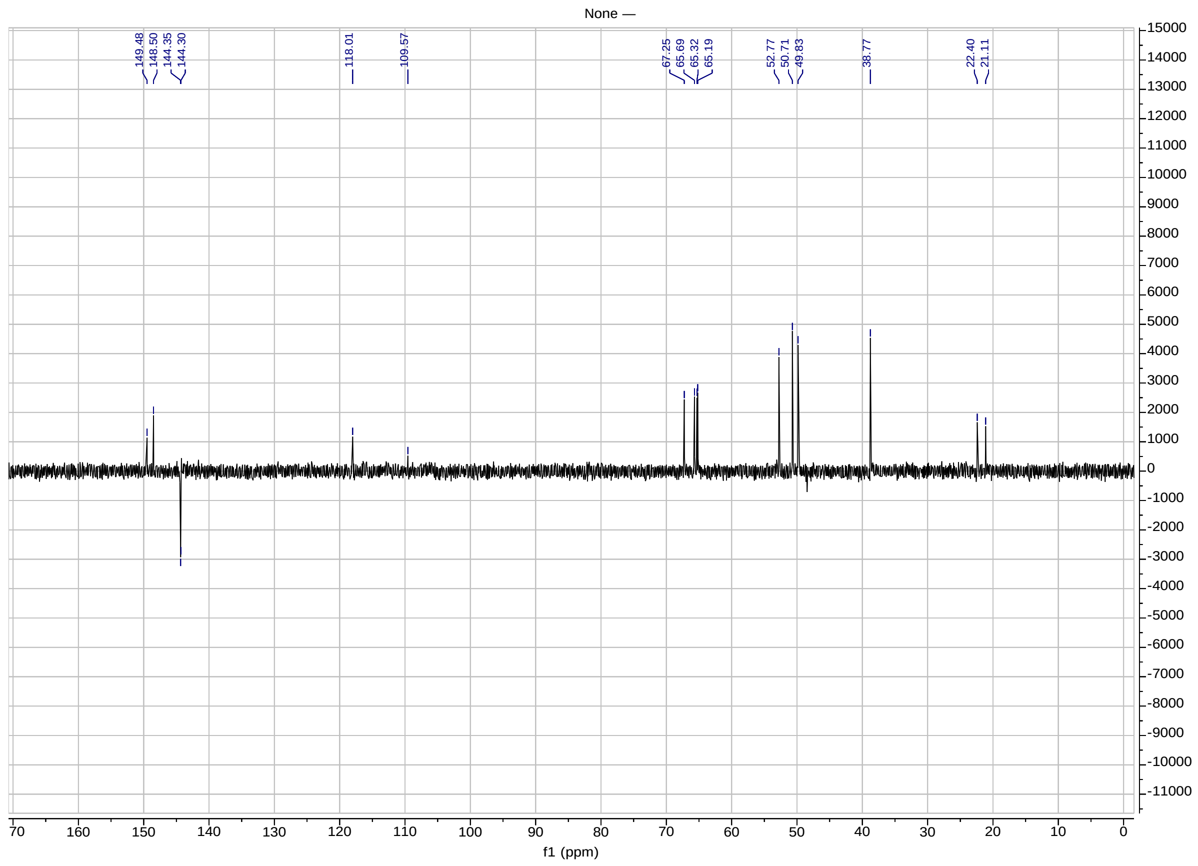

None —
149.48
148.50
144.35
144.30
118.01
109.57
67.25
65.69
65.32
65.19
52.77
50.71
49.83
38.77
22.40
21.11
f1 (ppm)

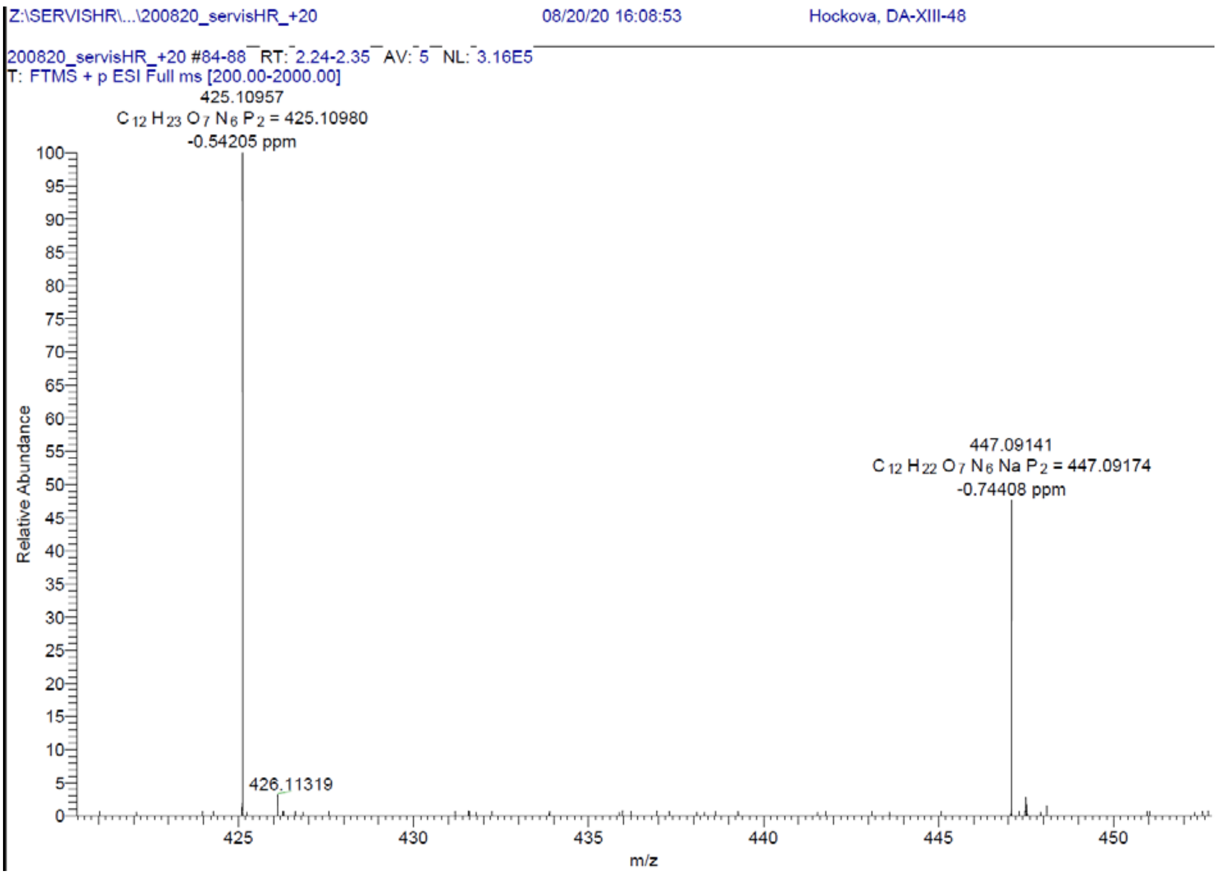

Z:\SERVISHR\...\200820_servisHR_+20
08/20/20 16:08:53
Hockova, DA-XIII-48
200820_servisHR_+20 #84-88 RT: 2.24-2.35 AV: 5 NL: 3.16E5
T: FTMS + p ESI Full ms [200.00-2000.00]
425.10957
C 12 H 23 O 7 N 6 P 2 = 425.10980
-0.54205 ppm
426.11319
447.09141
C 12 H 22 O 7 N 6 Na P 2 = 447.09174
-0.74408 ppm
Relative Abundance
m/z

9e

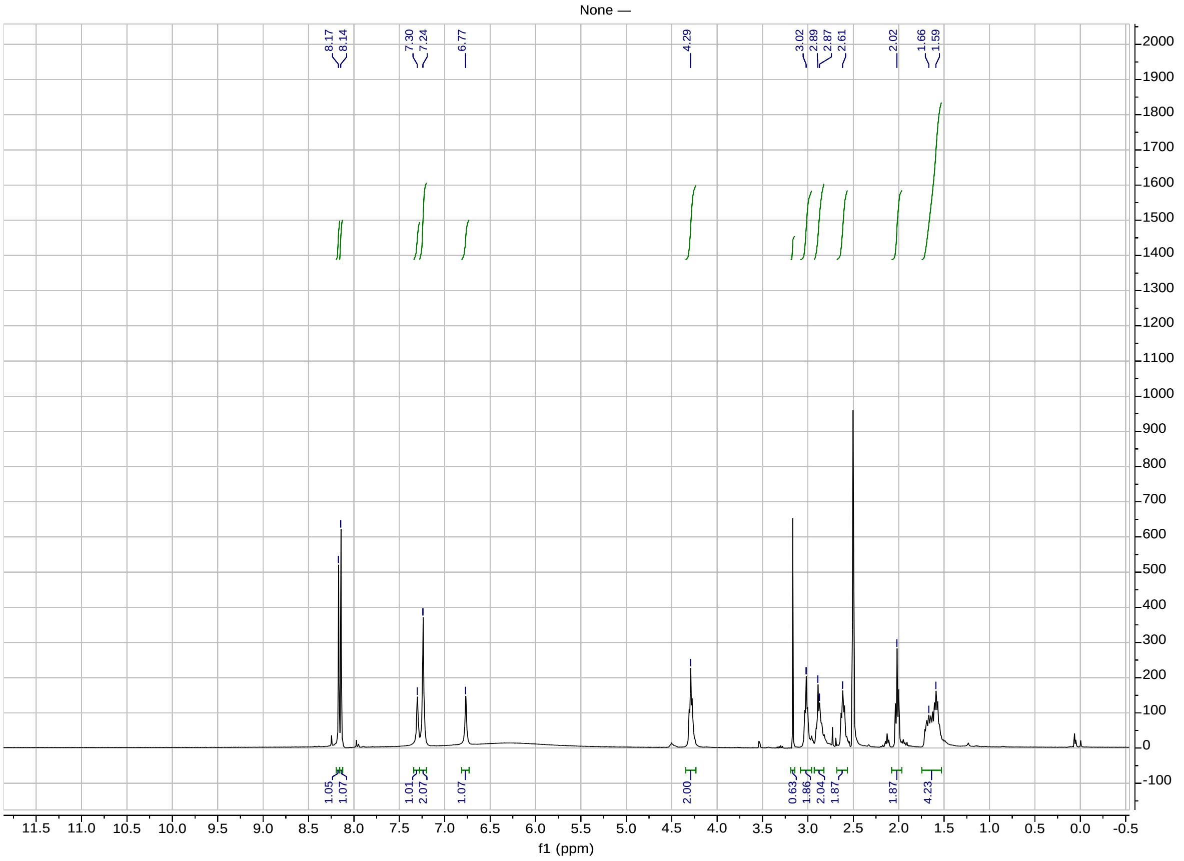
None —
8.17
8.14
7.30
7.24
6.77
4.29
3.02
2.89
2.87
2.61
2.02
1.66
1.59
1.05
1.07
1.01
2.07
1.07
2.00
0.63
1.86
2.04
1.87
1.87
4.23
f1 (ppm)

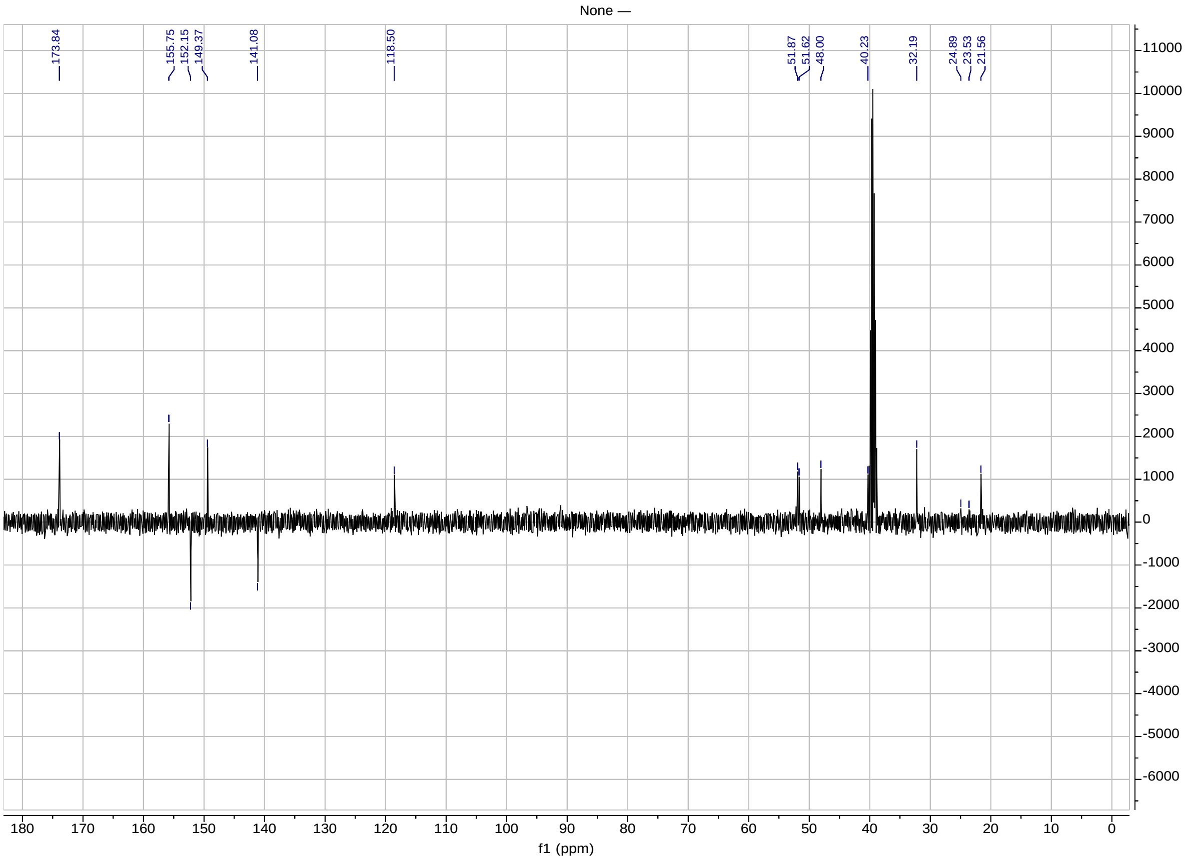
None —
173.84
155.75
152.15
149.37
141.08
118.50
51.87
51.62
48.00
40.23
32.19
24.89
23.53
21.56
f1 (ppm)

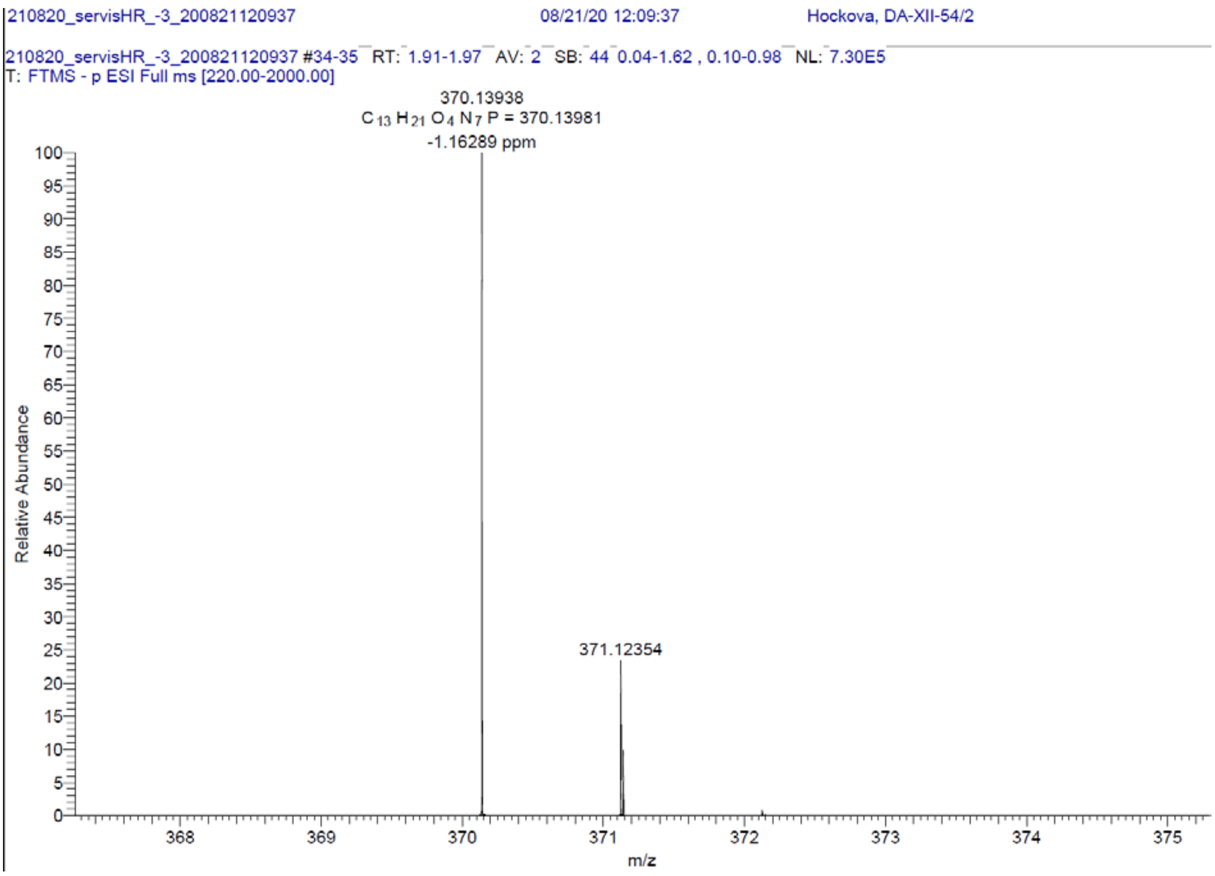
210820_servisHR_-3_200821120937
08/21/20 12:09:37
Hockova, DA-XII-54/2
210820_servisHR_-3_200821120937 #34-35 RT: 1.91-1.97 AV: 2 SB: 44 0.04-1.62 , 0.10-0.98 NL: 7.30E5
T: FTMS - p ESI Full ms [220.00-2000.00]
370.13938
C 13 H 21 O 4 N 7 P = 370.13981
-1.16289 ppm
371.12354
Relative Abundance
m/z

9f

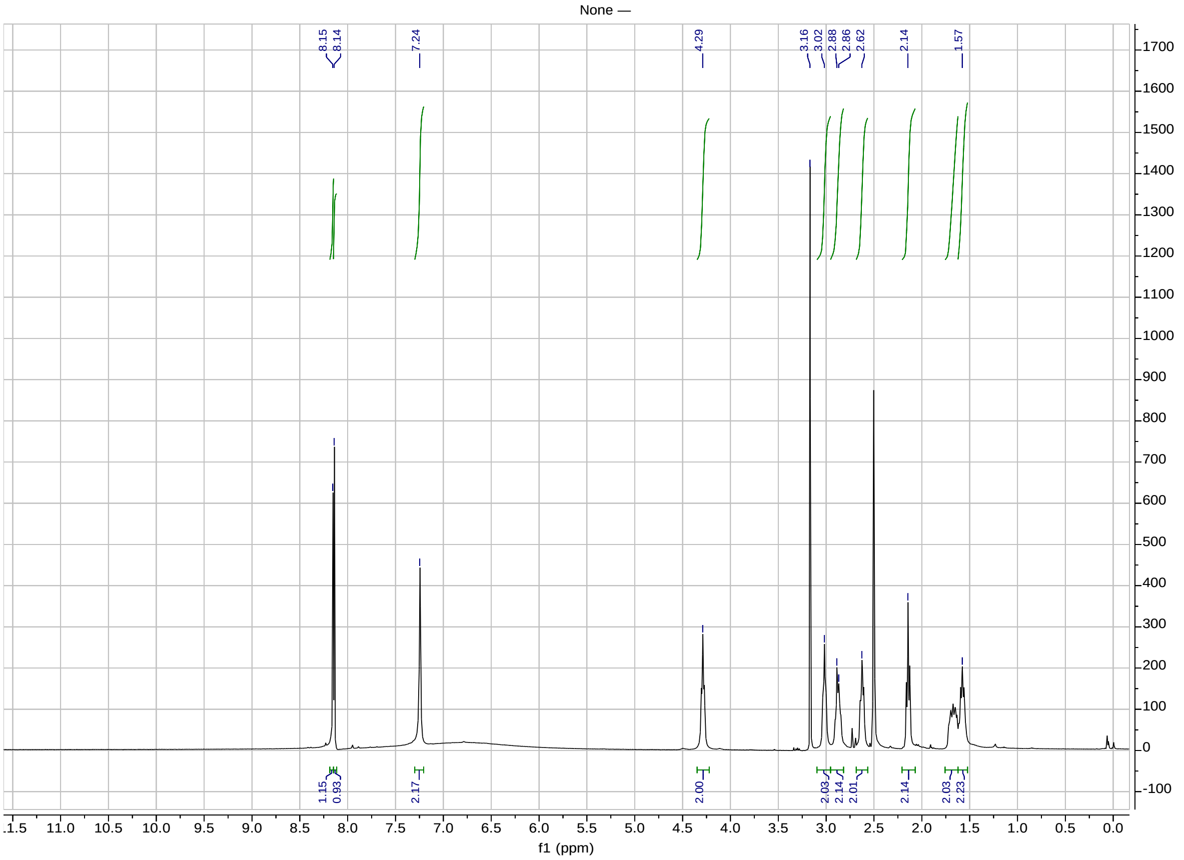
None —
8.15
8.14
7.24
4.29
3.16
3.02
2.88
2.86
2.62
2.14
1.57
1.15
0.93
2.17
2.00
2.03
2.14
2.01
2.14
2.03
2.23
f1 (ppm)

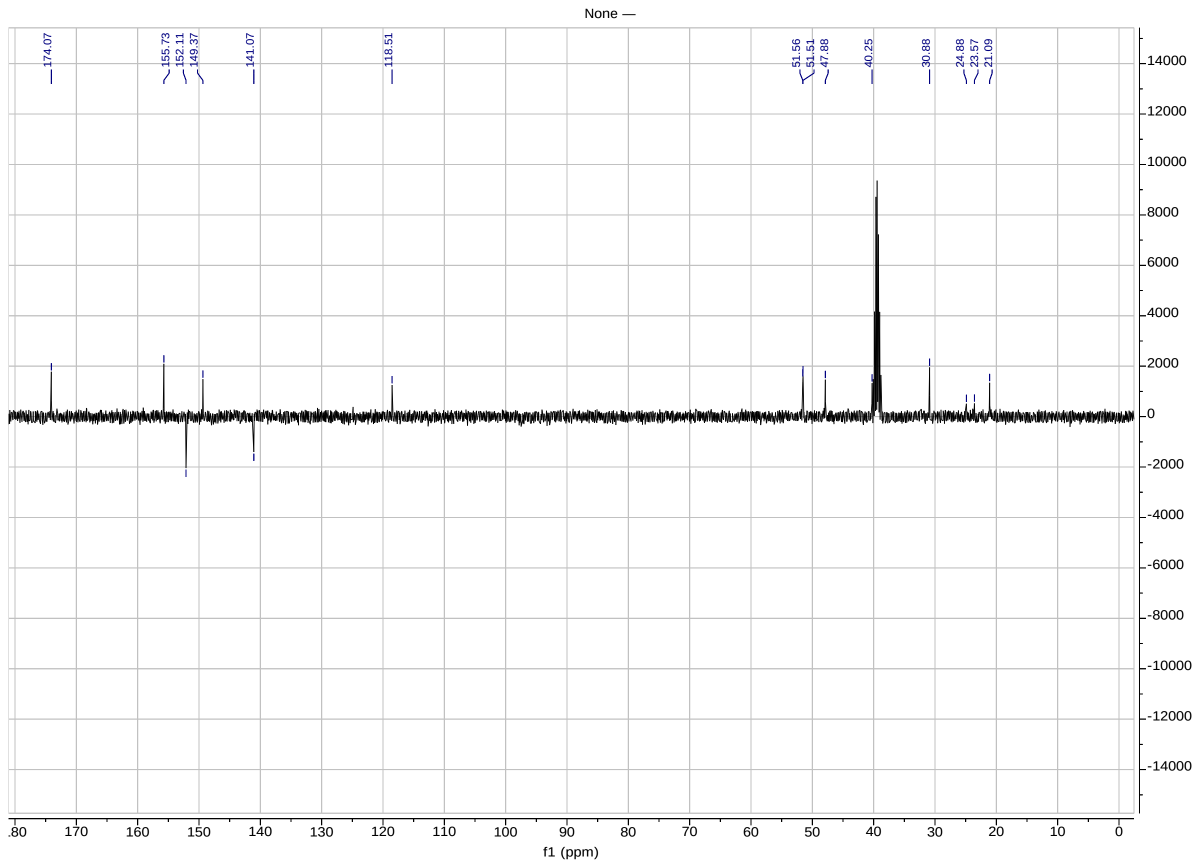
None —
174.07
155.73
152.11
149.37
141.07
118.51
51.56
51.51
47.88
40.25
30.88
24.88
23.57
21.09
f1 (ppm)

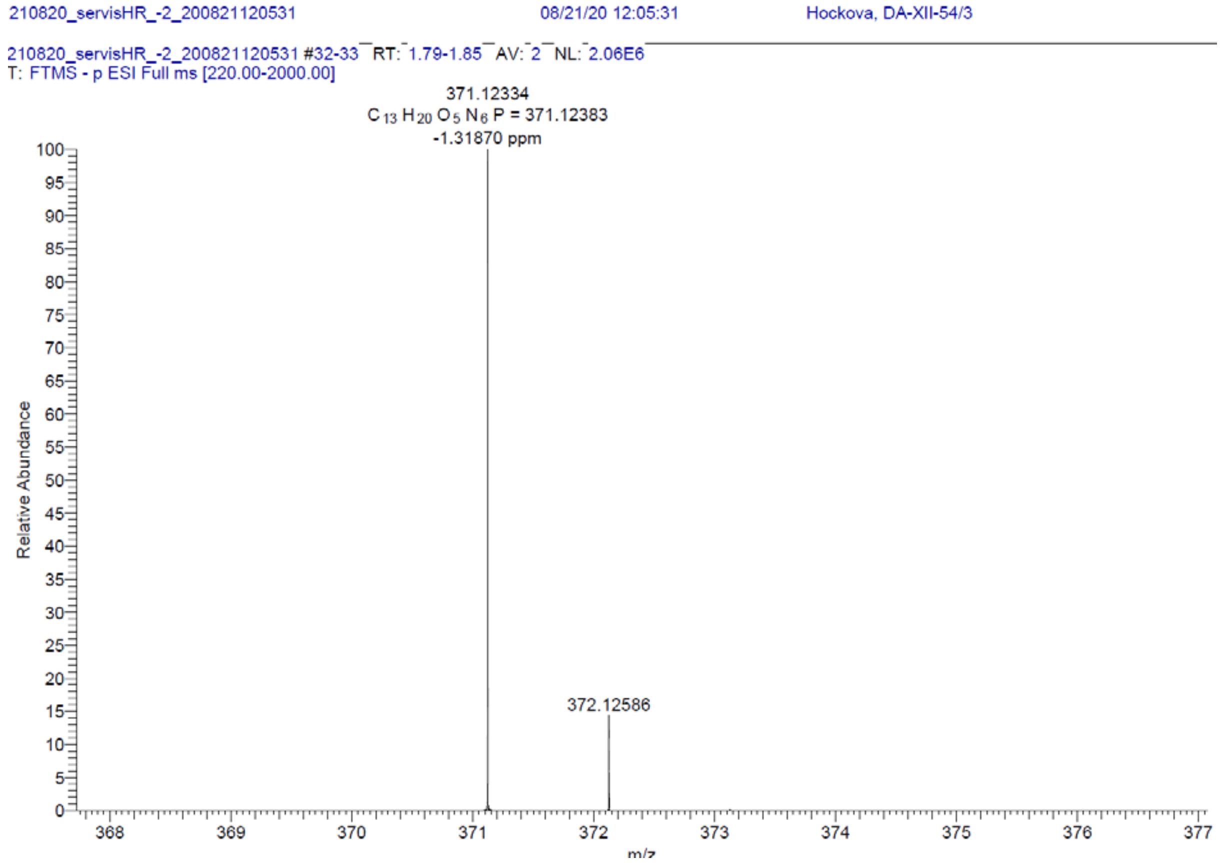
210820_servisHR_-2_200821120531
08/21/20 12:05:31
Hockova, DA-XII-54/3
210820_servisHR_-2_200821120531 #32-33 RT: 1.79-1.85 AV: 2 NL: 2.06E6
T: FTMS - p ESI Full ms [220.00-2000.00]
371.12334
C 13 H 20 O 5 N 6 P = 371.12383
-1.31870 ppm
372.12586
Relative Abundance
m/z

9g

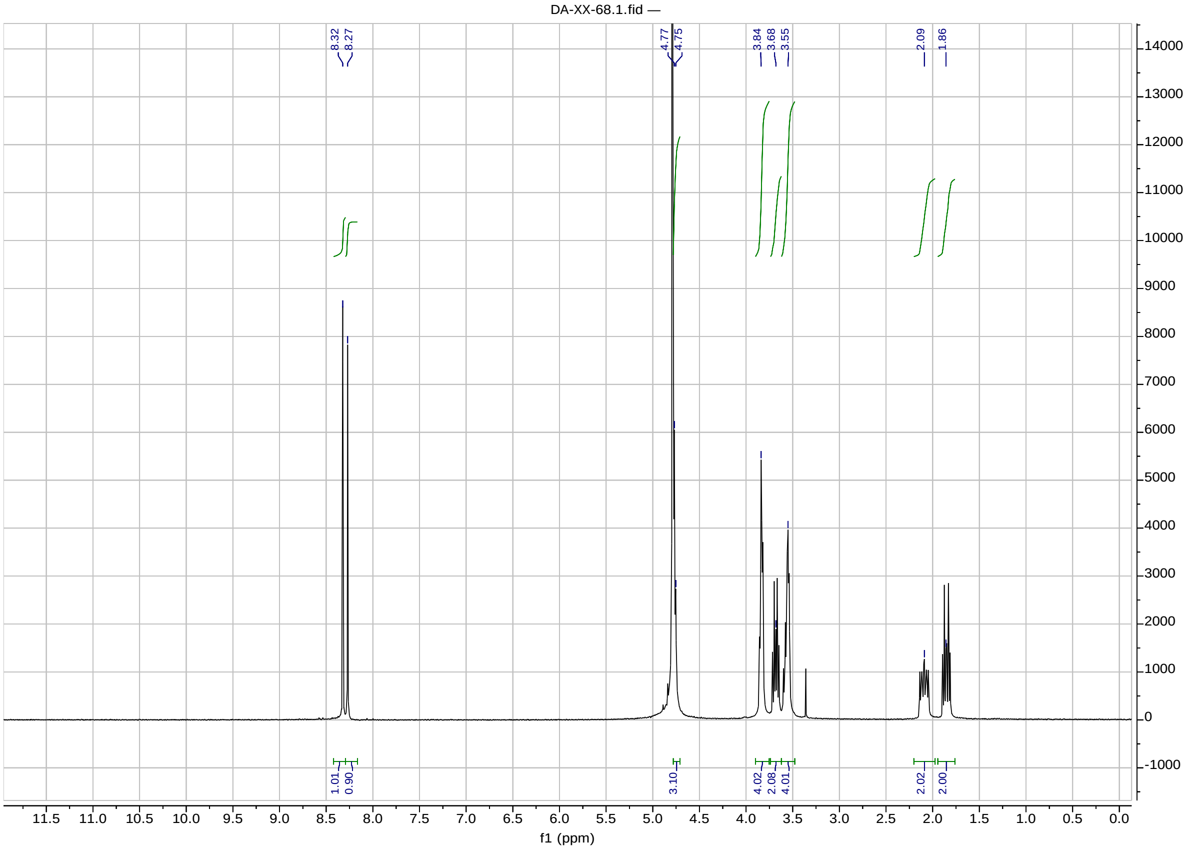
DA-XX-68.1.fid —
8.32
8.27
4.77
4.75
3.84
3.68
3.55
2.09
1.86
1.01
0.90
3.10
4.02
2.08
4.01
2.02
2.00
f1 (ppm)

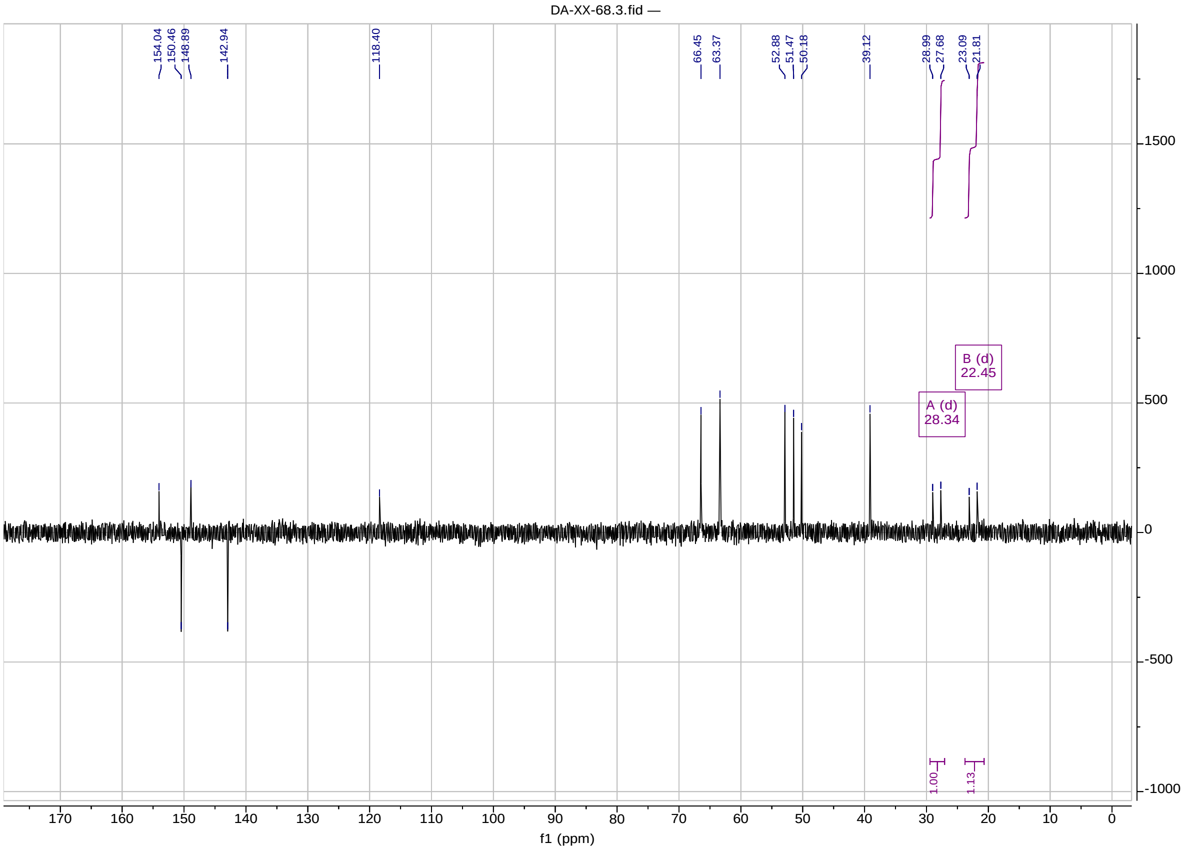
DA-XX-68.3.fid —
154.04
150.46
148.89
142.94
118.40
66.45
63.37
52.88
51.47
50.18
39.12
28.99
27.68
23.09
21.81
B (d)
22.45
A (d)
28.34
1.00
1.13
f1 (ppm)

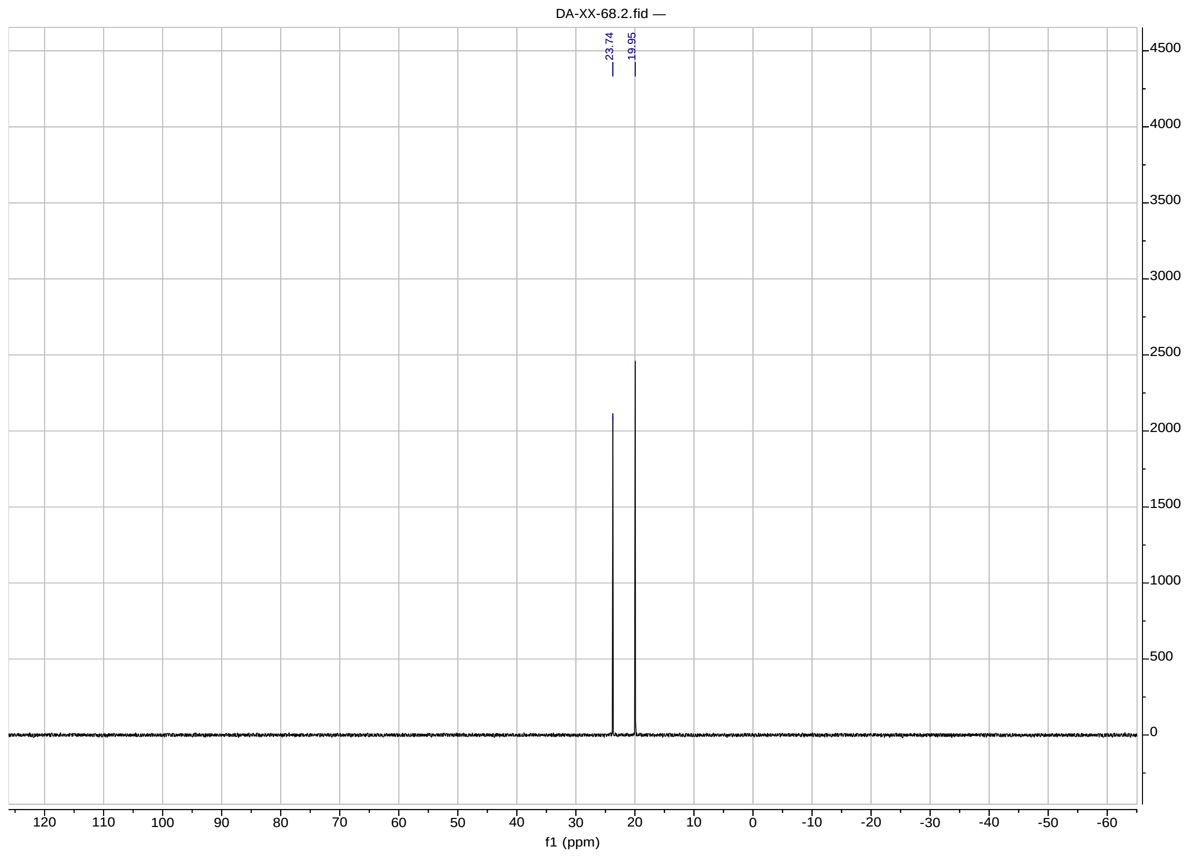
DA-XX-68.2.fid —
23.74
19.95
4500
4000
3500
3000
2500
2000
1500
1000
500
0
120 110 100 90 80 70 60 50 40 30 20 10 0 -10 -20 -30 -40 -50 -60
f1 (ppm)

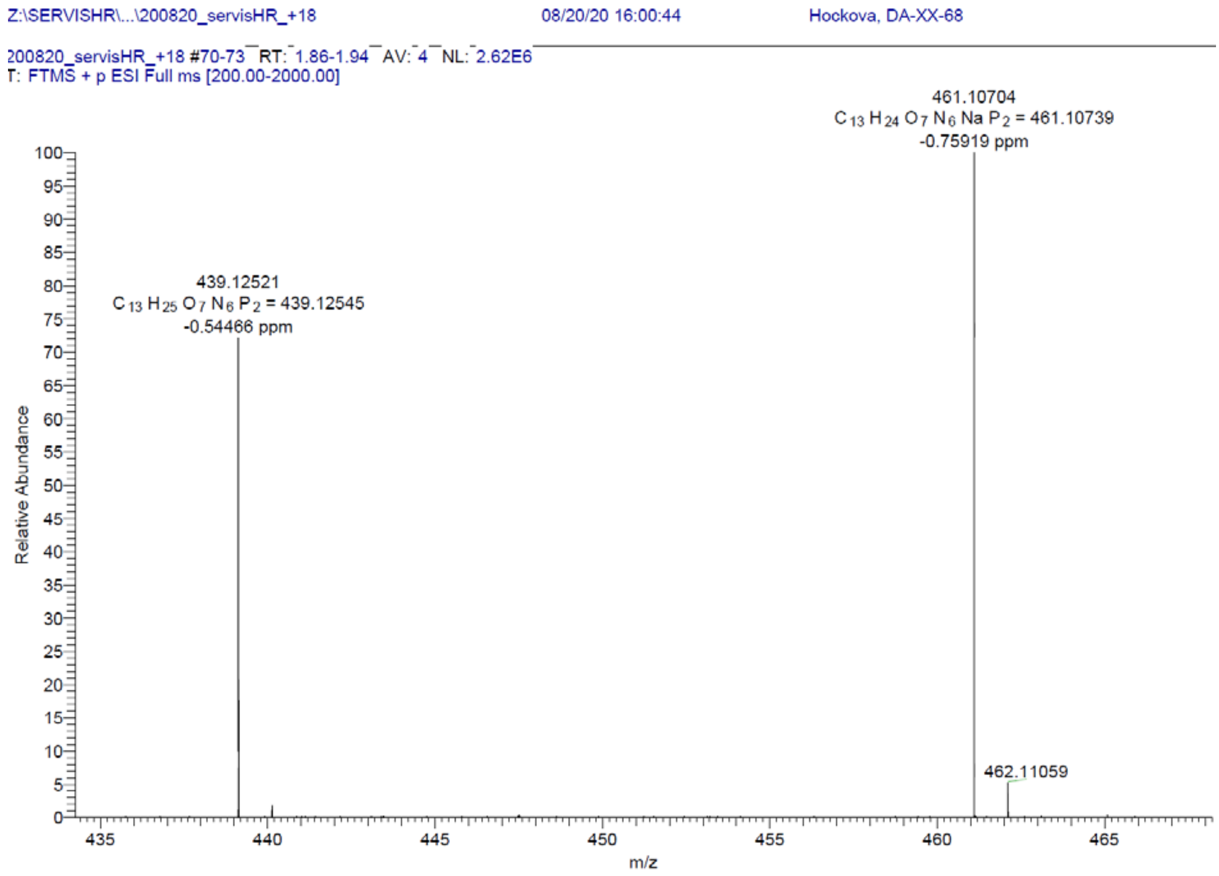
Z:\SERVISHR\...\200820_servisHR_+18
08/20/20 16:00:44
Hockova, DA-XX-68
200820_servisHR_+18 #70-73 RT: 1.86-1.94 AV: 4 NL: 2.62E6
T: FTMS + p ESI Full ms [200.00-2000.00]
461.10704
C 13 H 24 O 7 N 6 Na P 2 = 461.10739
-0.75919 ppm
439.12521
C 13 H 25 O 7 N 6 P 2 = 439.12545
-0.54466 ppm
462.11059
Relative Abundance
m/z
435 440 445 450 455 460 465

17

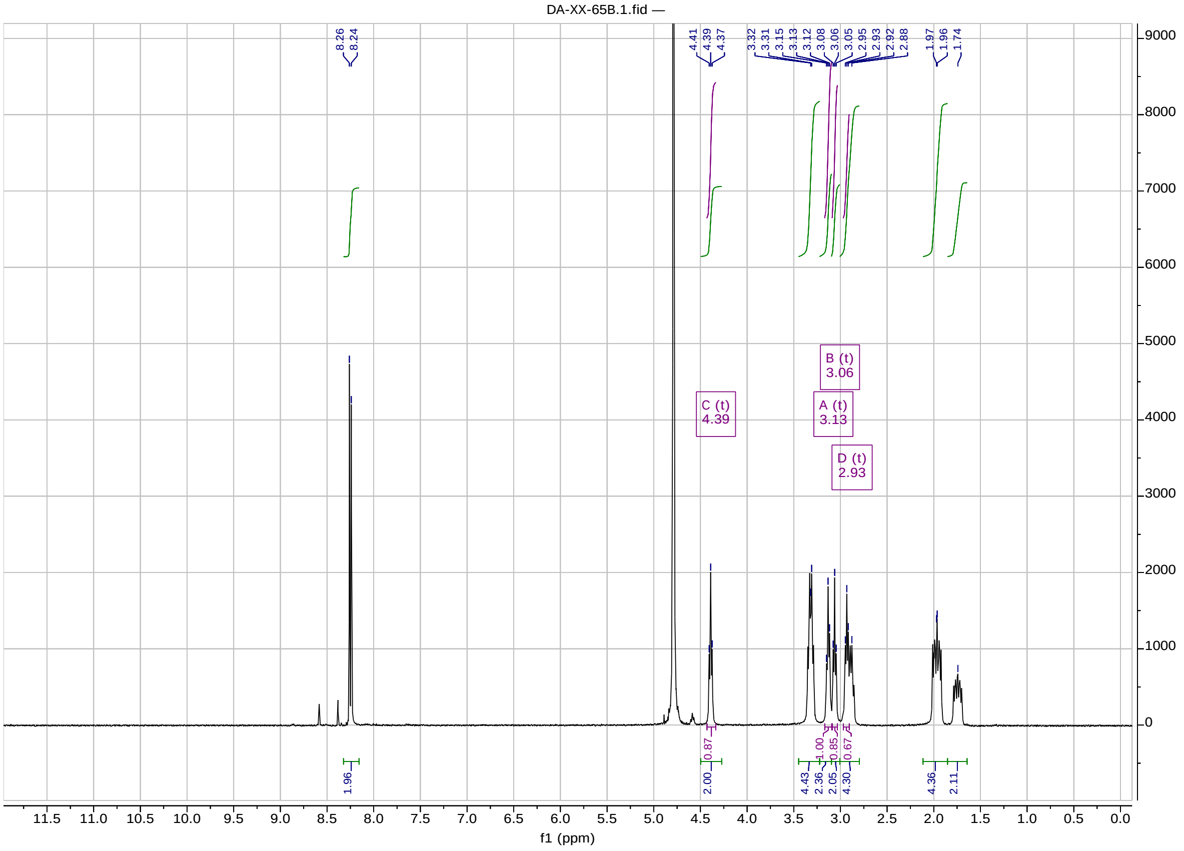
DA-XX-65B.1.fid —
8.26
8.24
4.41
4.39
4.37
3.32
3.31
3.15
3.13
3.12
3.08
3.06
3.05
2.95
2.93
2.92
2.88
1.97
1.96
1.74
B (t)
3.06
C (t)
4.39
A (t)
3.13
D (t)
2.93
1.96
2.00
0.87
4.43
2.36
1.00
2.05
0.85
4.30
0.67
4.36
2.11
f1 (ppm)

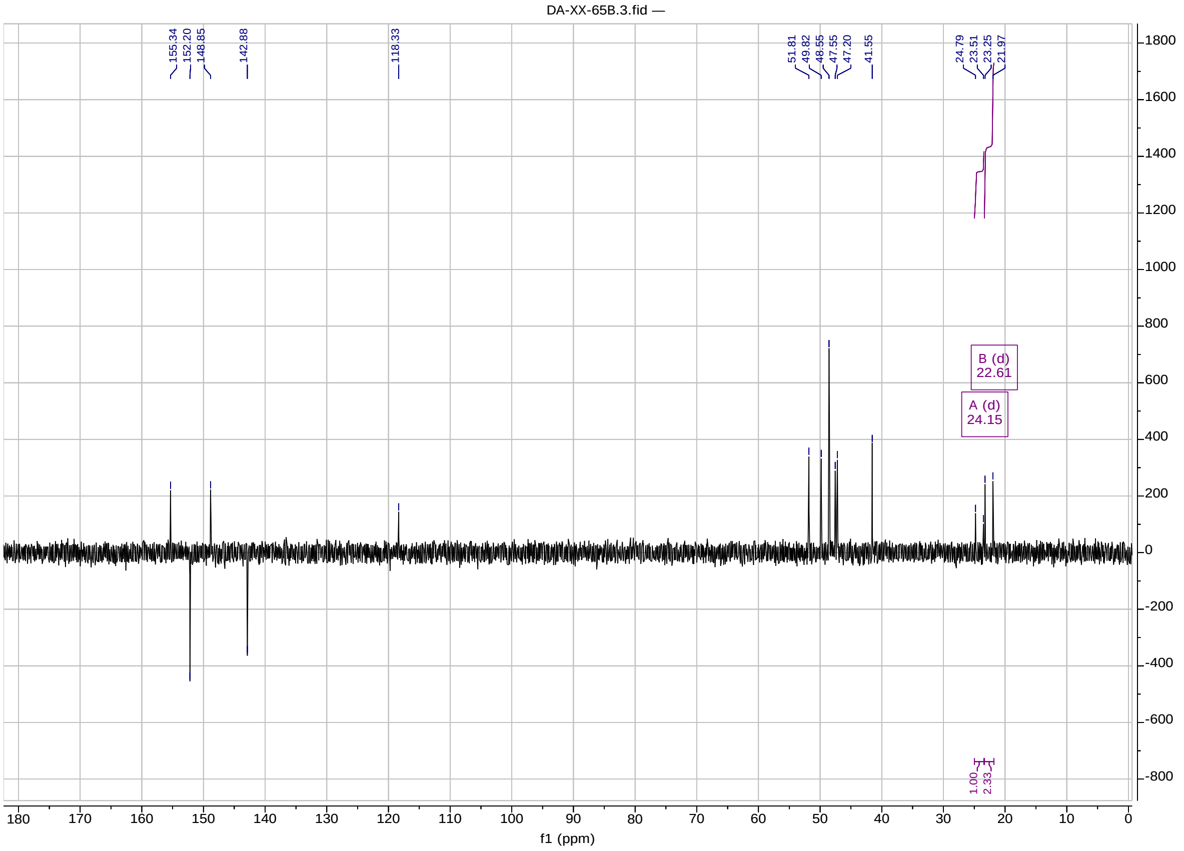
DA-XX-65B.3.fid —
155.34
152.20
148.85
142.88
118.33
51.81
49.82
48.55
47.55
47.20
41.55
24.79
23.51
23.25
21.97
B (d)
22.61
A (d)
24.15
1.00
2.33
f1 (ppm)

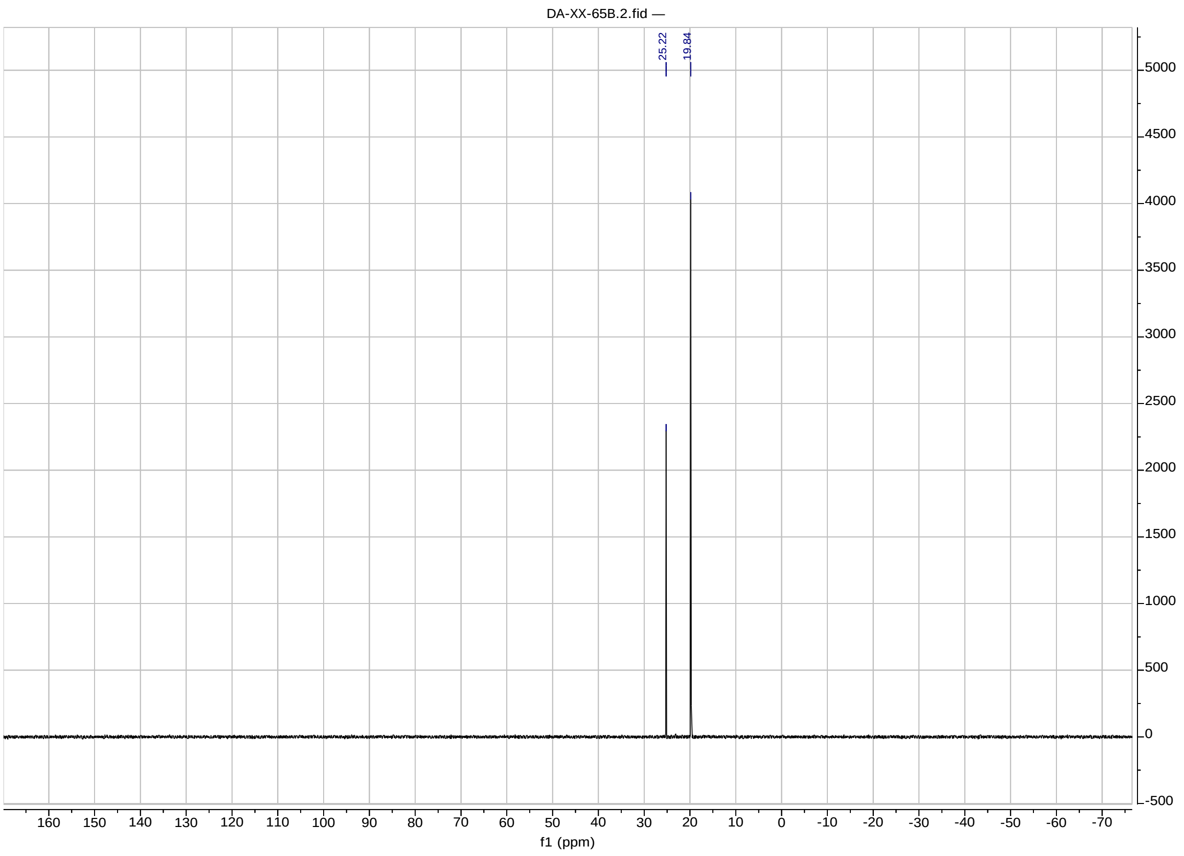
DA-XX-65B.2.fid —
25.22
19.84
f1 (ppm)

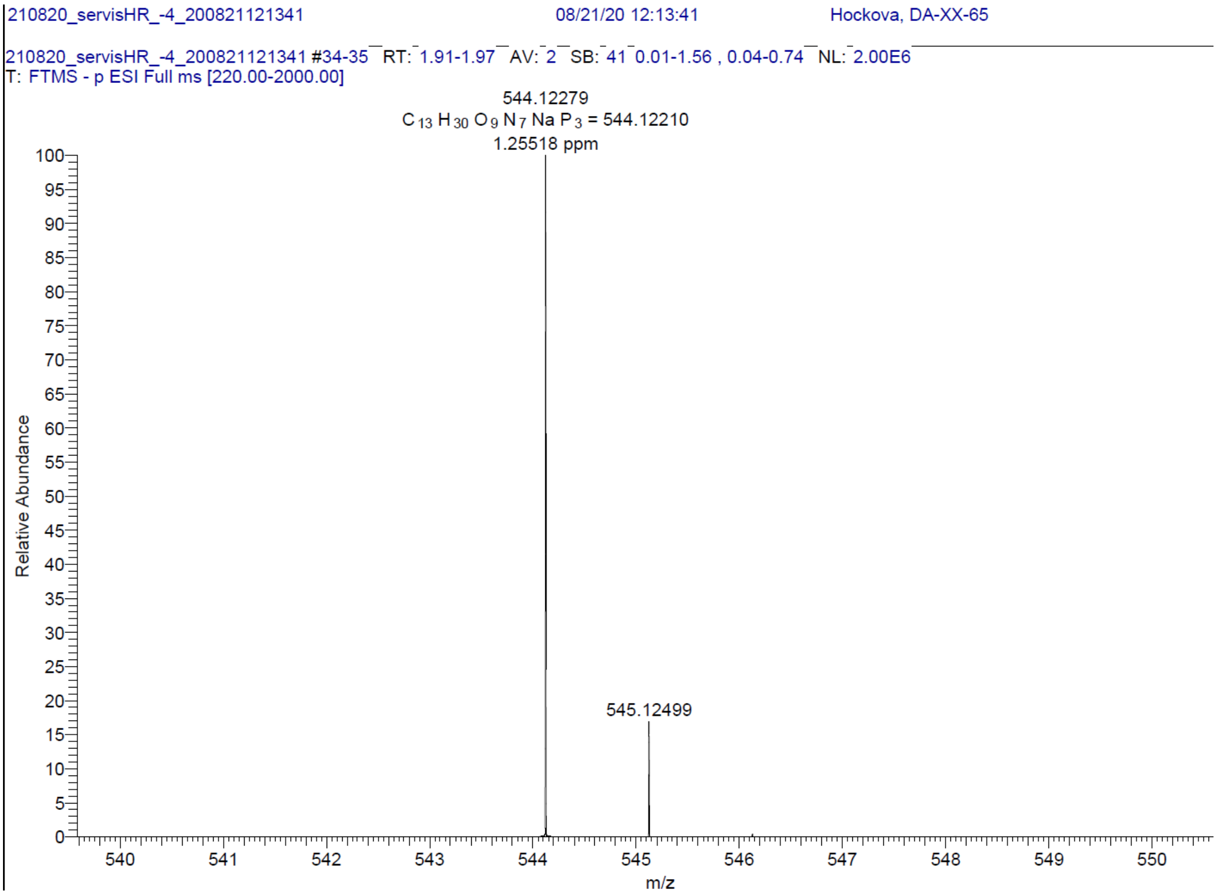
210820_servisHR_-4_200821121341
08/21/20 12:13:41
Hockova, DA-XX-65
210820_servisHR_-4_200821121341 #34-35 RT: 1.91-1.97 AV: 2 SB: 41 0.01-1.56 , 0.04-0.74 NL: 2.00E6
T: FTMS - p ESI Full ms [220.00-2000.00]
544.12279
C 13 H 30 O 9 N 7 Na P 3 = 544.12210
1.25518 ppm
545.12499
Relative Abundance
m/z

20

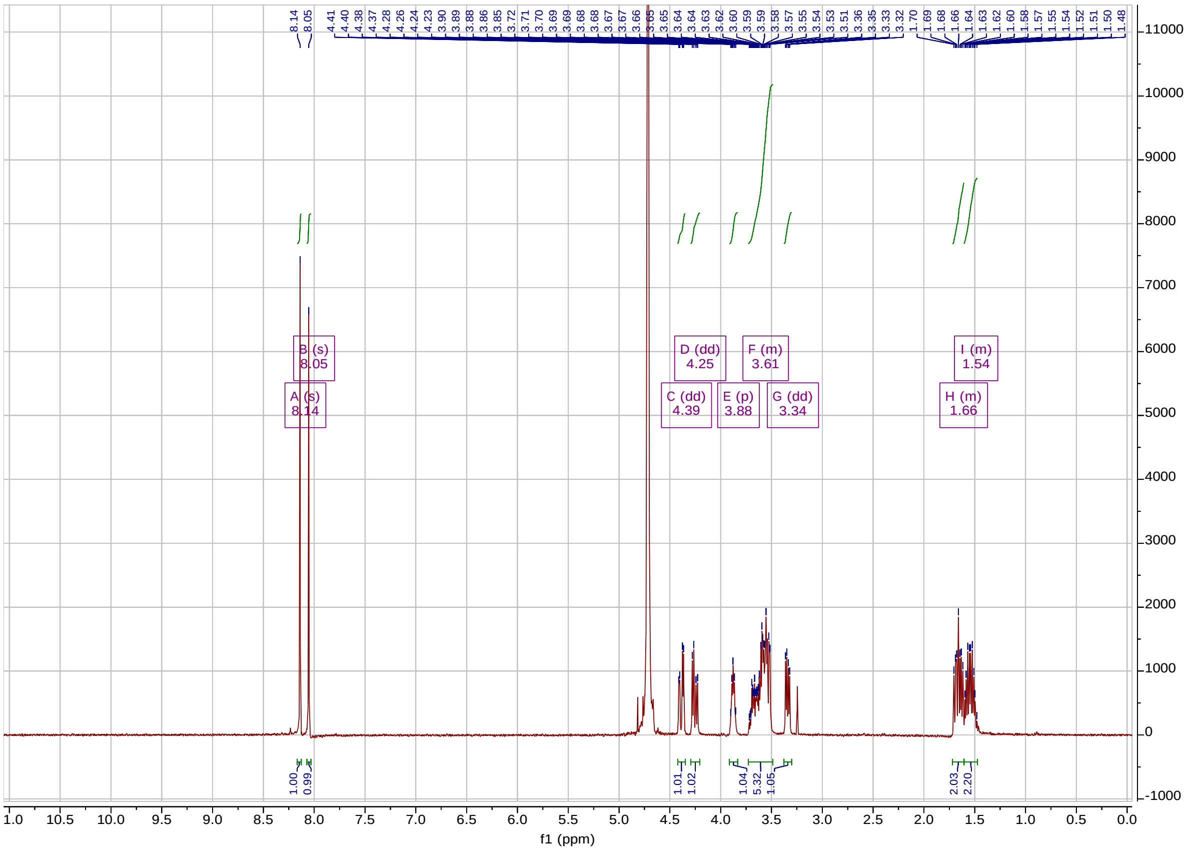
A (s) 8.14
B (s) 8.05
C (dd) 4.39
D (dd) 4.25
E (p) 3.88
F (m) 3.61
G (dd) 3.34
H (m) 1.66
I (m) 1.54
1.00
0.99
1.01
1.02
1.04
5.32
1.05
2.03
2.20
f1 (ppm)

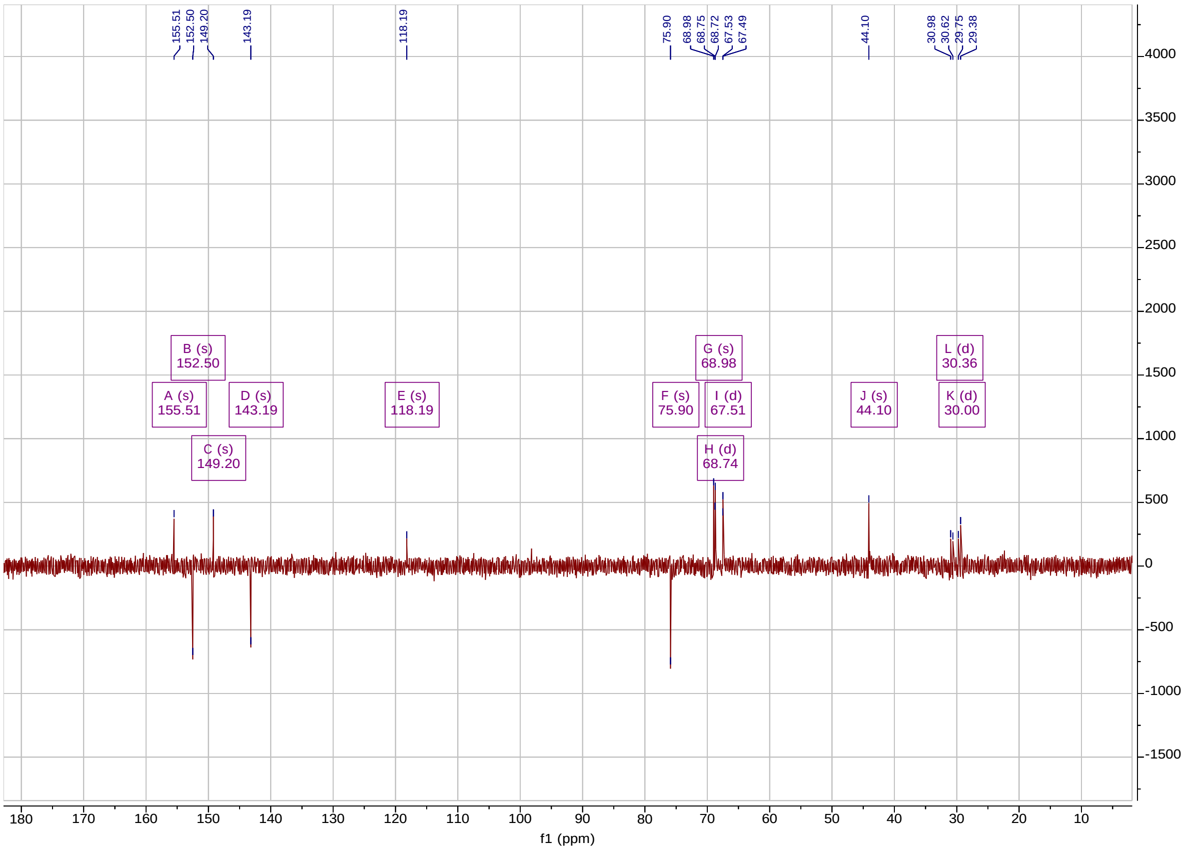
155.51
152.50
149.20
143.19
118.19
75.90
68.98
68.75
68.72
67.53
67.49
44.10
30.98
30.62
29.75
29.38
A (s) 155.51
B (s) 152.50
C (s) 149.20
D (s) 143.19
E (s) 118.19
F (s) 75.90
G (s) 68.98
H (d) 68.74
I (d) 67.51
J (s) 44.10
K (d) 30.00
L (d) 30.36
f1 (ppm)

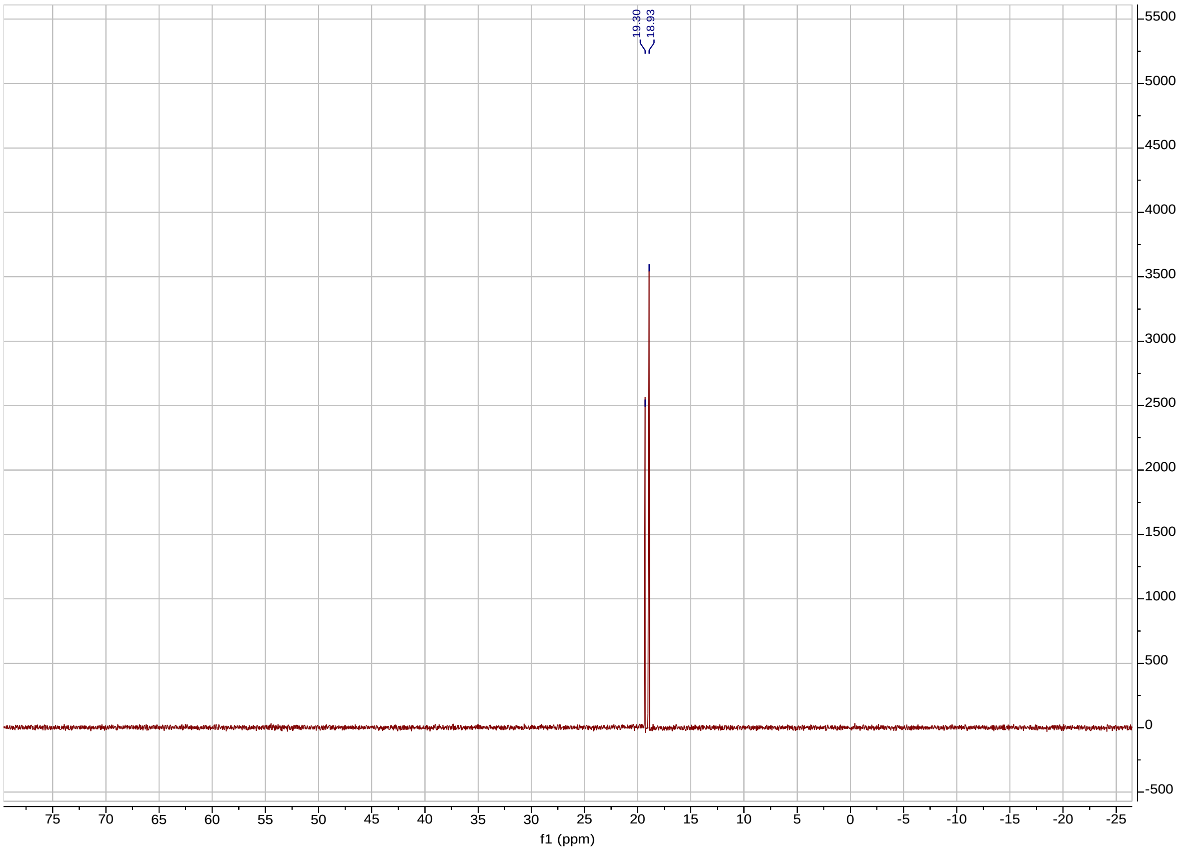

19.30
18.93
5500
5000
4500
4000
3500
3000
2500
2000
1500
1000
500
0
-500
75 70 65 60 55 50 45 40 35 30 25 20 15 10 5 0 -5 -10 -15 -20 -25
f1 (ppm)

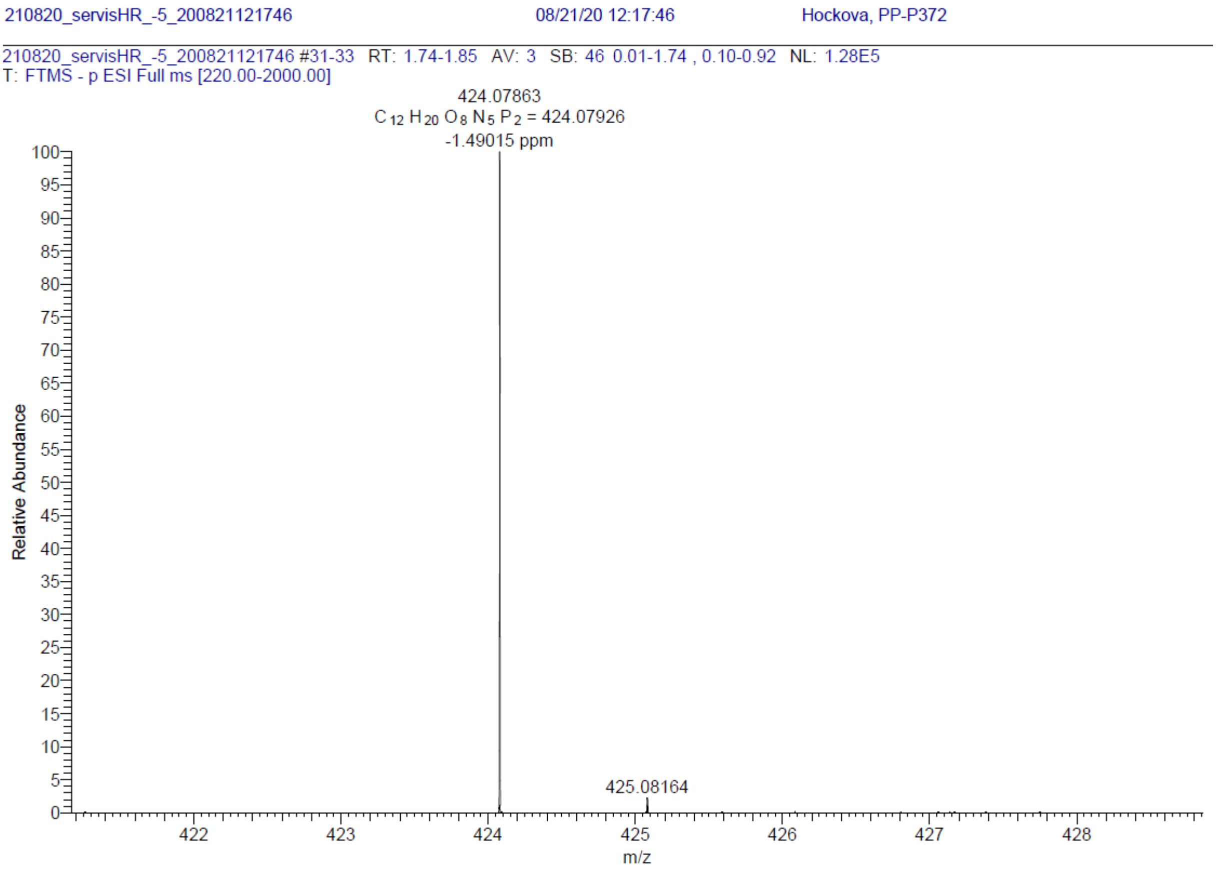

210820_servisHR_-5_200821121746
08/21/20 12:17:46
Hockova, PP-P372
210820_servisHR_-5_200821121746 #31-33 RT: 1.74-1.85 AV: 3 SB: 46 0.01-1.74 , 0.10-0.92 NL: 1.28E5
T: FTMS - p ESI Full ms [220.00-2000.00]
424.07863
C 12 H 20 O 8 N 5 P 2 = 424.07926
-1.49015 ppm
425.08164
Relative Abundance
m/z

21

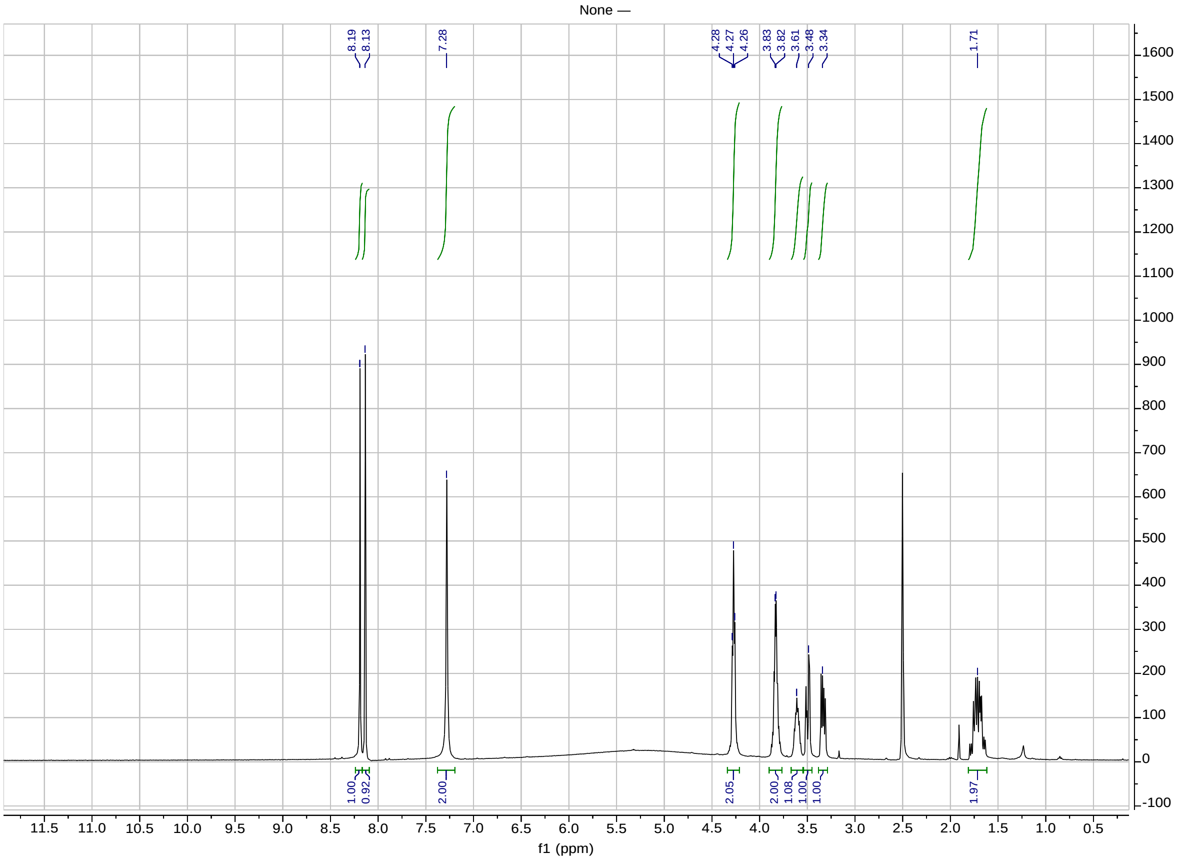
None —
8.19
8.13
7.28
4.28
4.27
4.26
3.83
3.82
3.61
3.48
3.34
1.71
1.00
0.92
2.00
2.05
2.00
1.08
1.00
1.00
1.97
f1 (ppm)

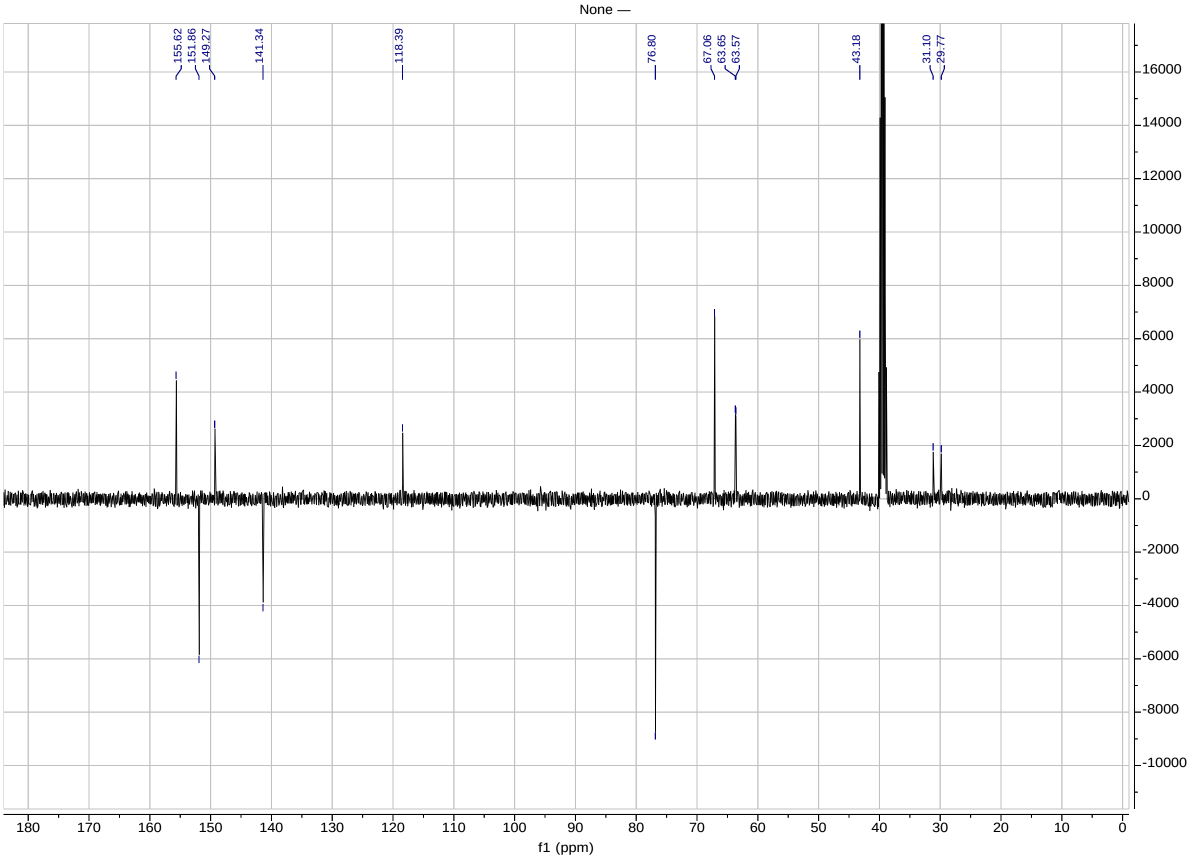
None —
155.62
151.86
149.27
141.34
118.39
76.80
67.06
63.65
63.57
43.18
31.10
29.77
f1 (ppm)

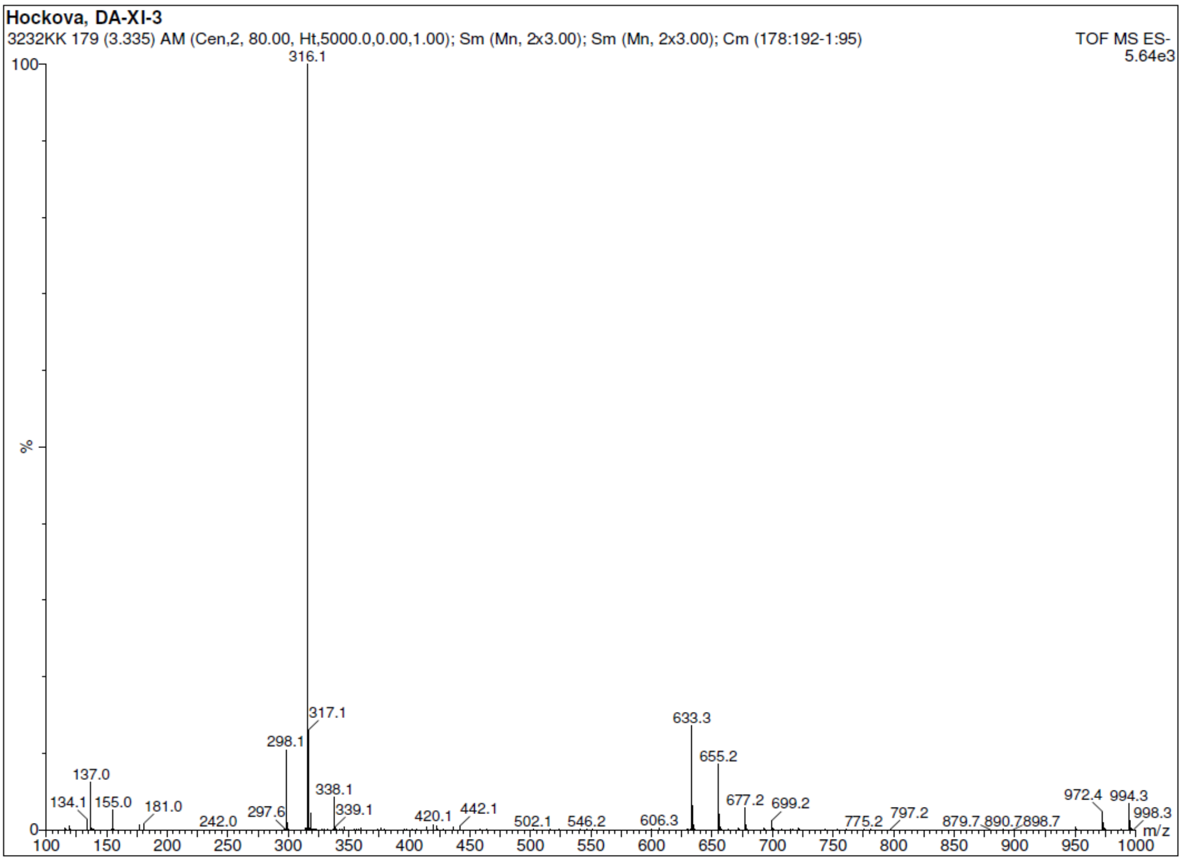

Hockova, DA-XI-3
3232KK 179 (3.335) AM (Cen,2, 80.00, Ht,5000.0,0.00,1.00); Sm (Mn, 2x3.00); Sm (Mn, 2x3.00); Cm (178:192-1:95)
TOF MS ES-
5.64e3
%
316.1
317.1
298.1
137.0
134.1
155.0
181.0
242.0
297.6
338.1
339.1
420.1
442.1
502.1
546.2
606.3
633.3
655.2
677.2
699.2
775.2
797.2
879.7
890.7
898.7
972.4
994.3
998.3
m/z

22

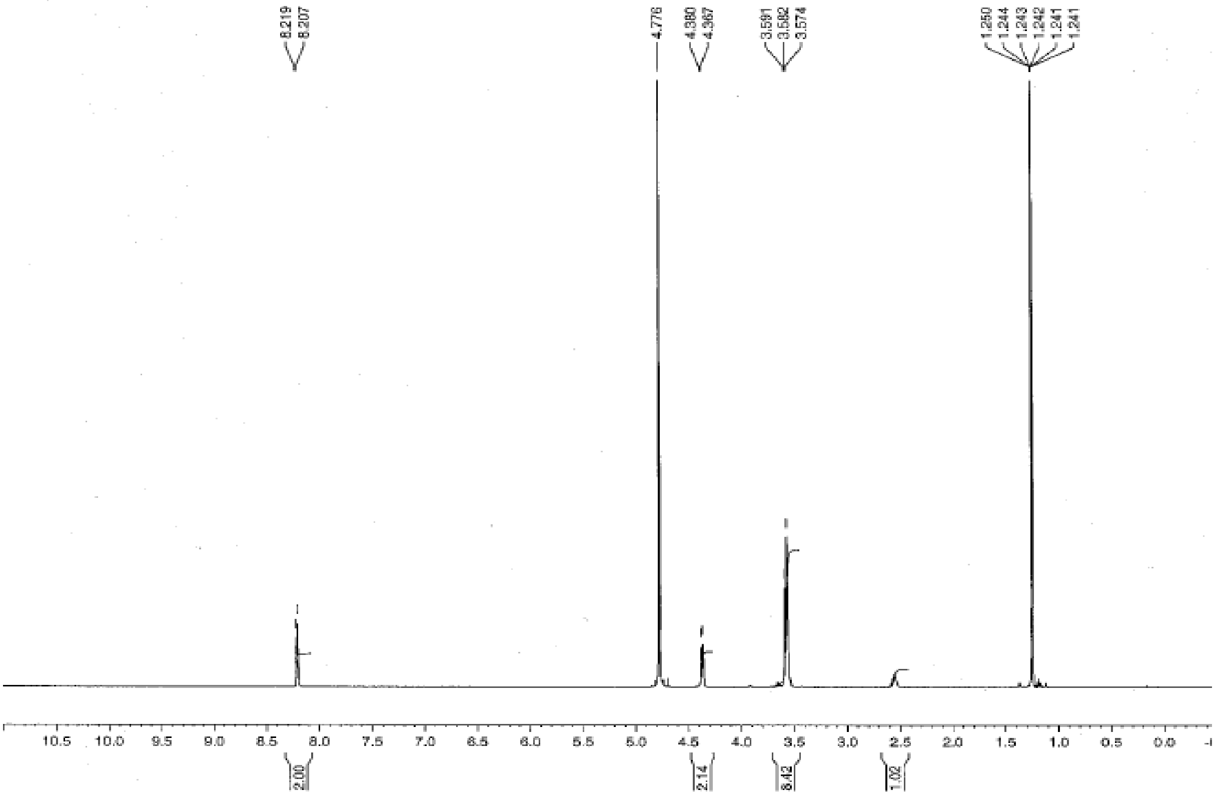

8.219
8.207
4.776
4.380
4.367
3.591
3.582
3.574
1.250
1.244
1.243
1.242
1.241
1.241
2.00
2.14
8.42
1.02

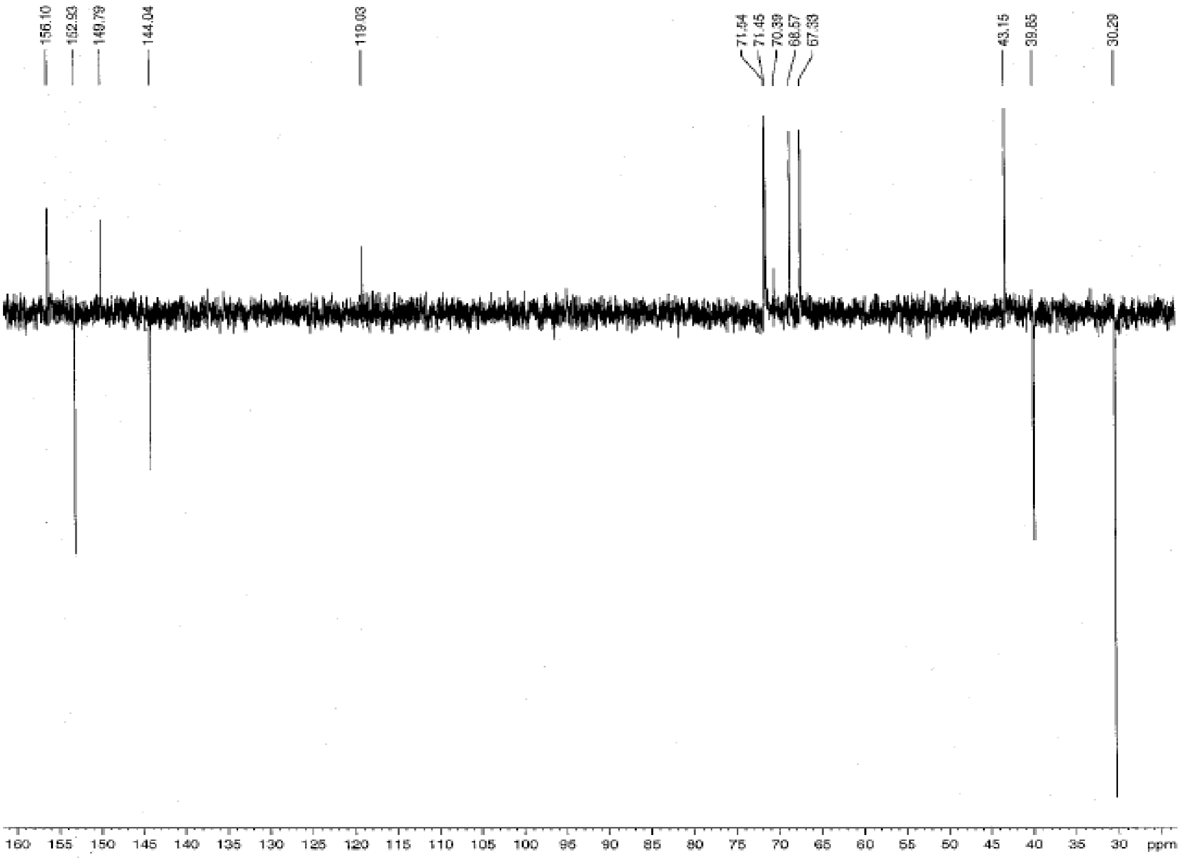
156.10
152.93
149.79
144.04
119.03
71.54
71.45
70.39
68.57
67.33
43.15
39.85
30.29
160 155 150 145 140 135 130 125 120 115 110 105 100 95 90 85 80 75 70 65 60 55 50 45 40 35 30 ppm

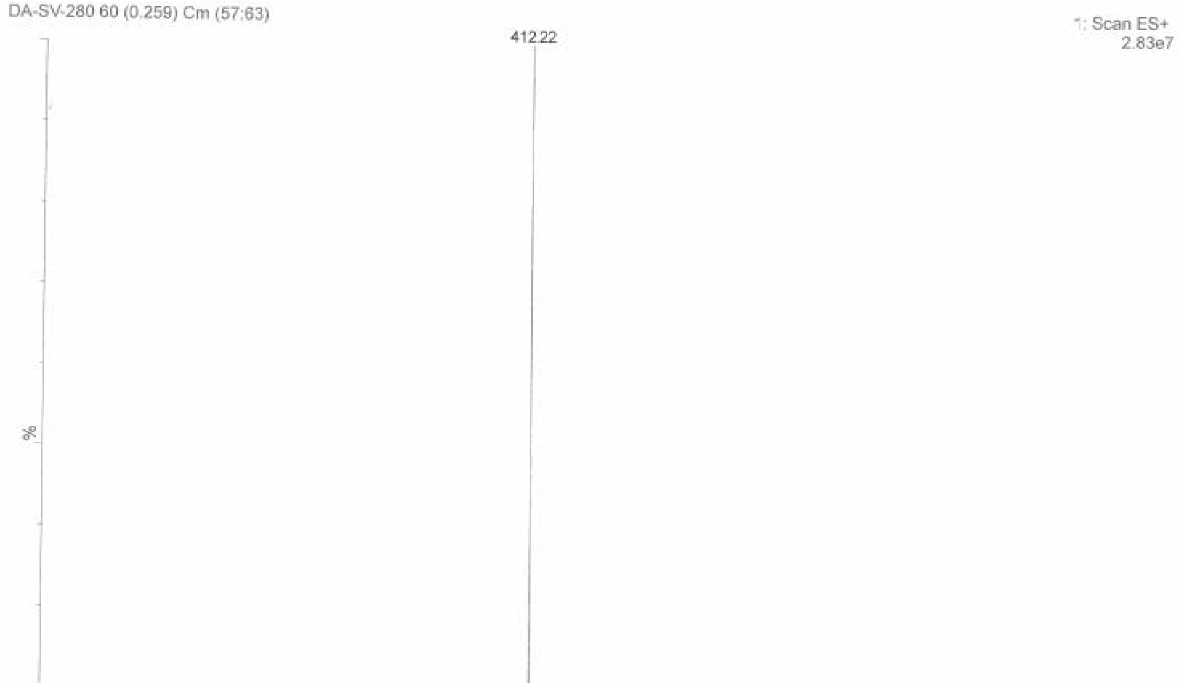
DA-SV-280 60 (0.259) Cm (57:63)
1: Scan ES+
2.83e7
412.22
413.15
102.38
%
2
100 150 200 250 300 350 400 450 500 550 600 650 700 750
m/z

24a

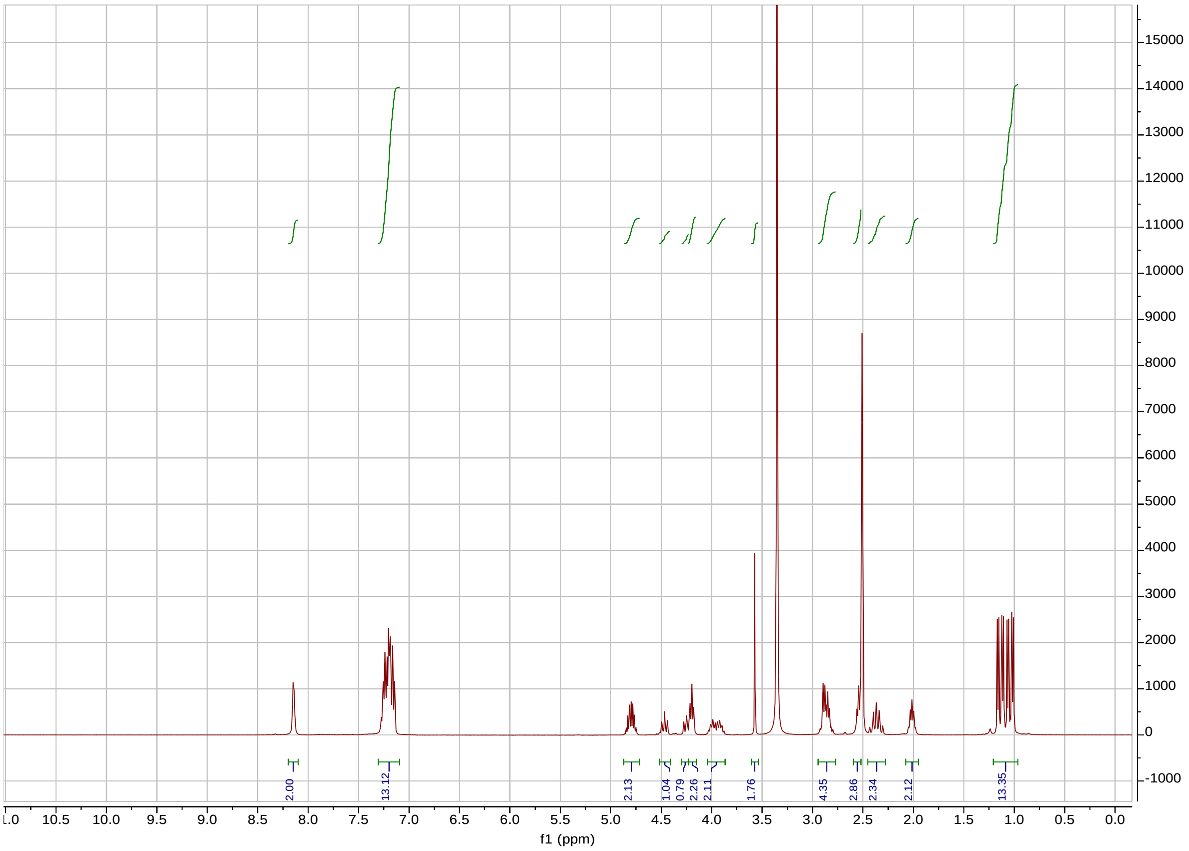

2.00
13.12
2.13
1.04
0.79
2.26
2.11
1.76
4.35
2.86
2.34
2.12
13.35
f1 (ppm)

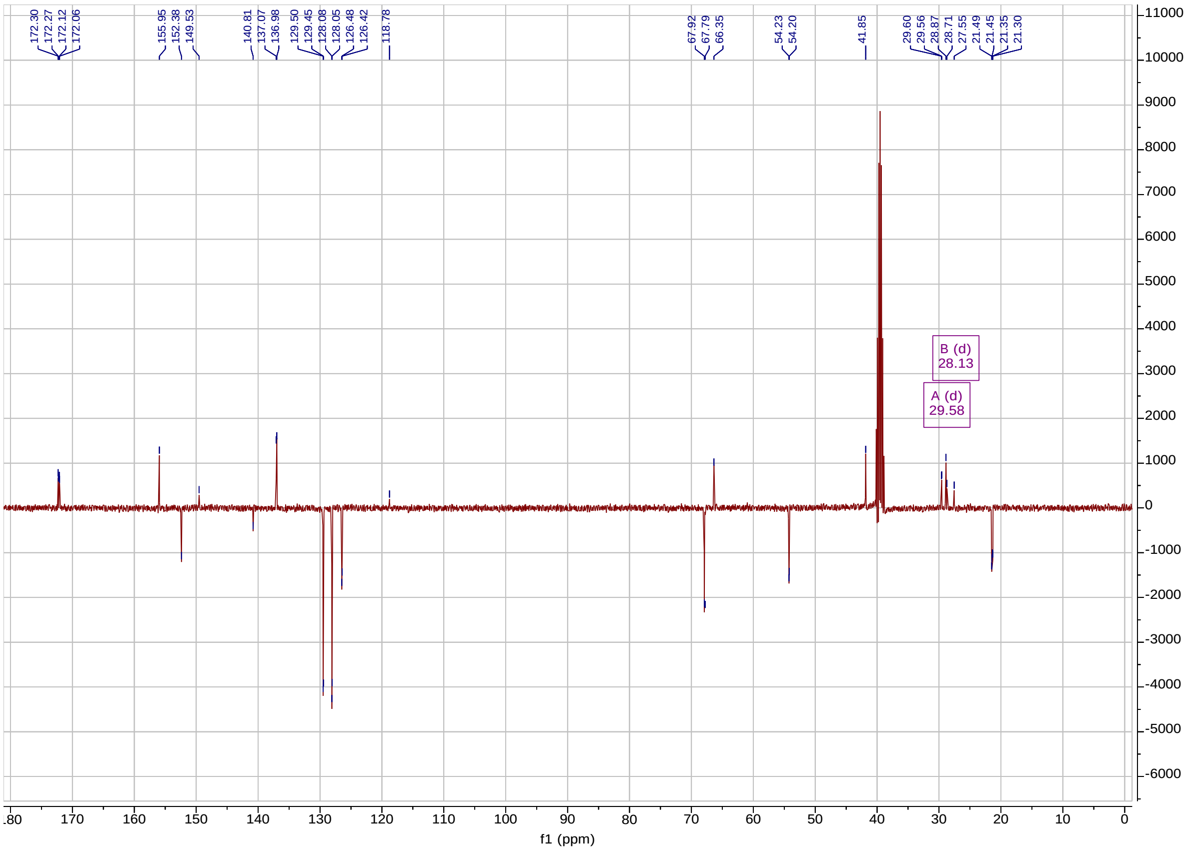

172.30
172.27
172.12
172.06
155.95
152.38
149.53
140.81
137.07
136.98
129.50
129.45
128.08
128.05
126.48
126.42
118.78
67.92
67.79
66.35
54.23
54.20
41.85
29.60
29.56
28.87
28.71
27.55
21.49
21.45
21.35
21.30
B (d)
28.13
A (d)
29.58
f1 (ppm)

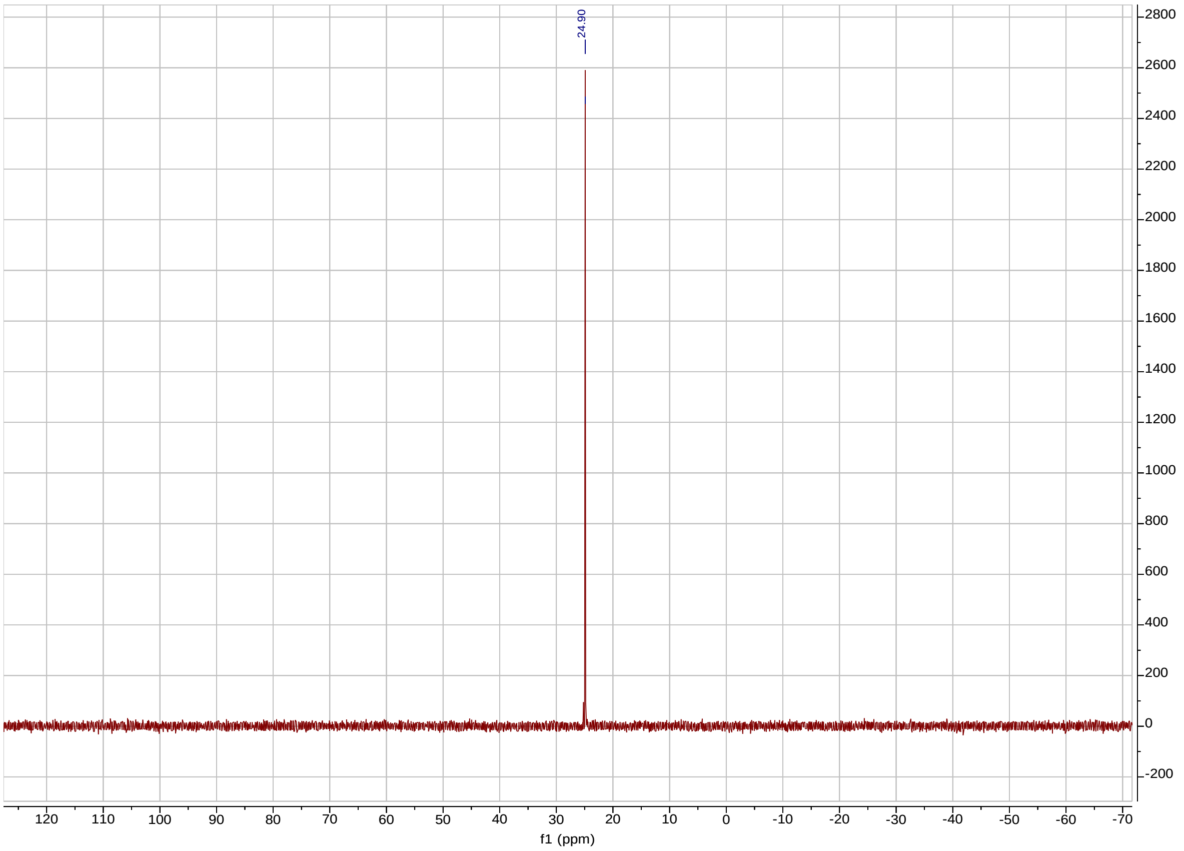

090620servis+HR_10_200617183715 06/17/20 18:37:15 Klejch, TK403

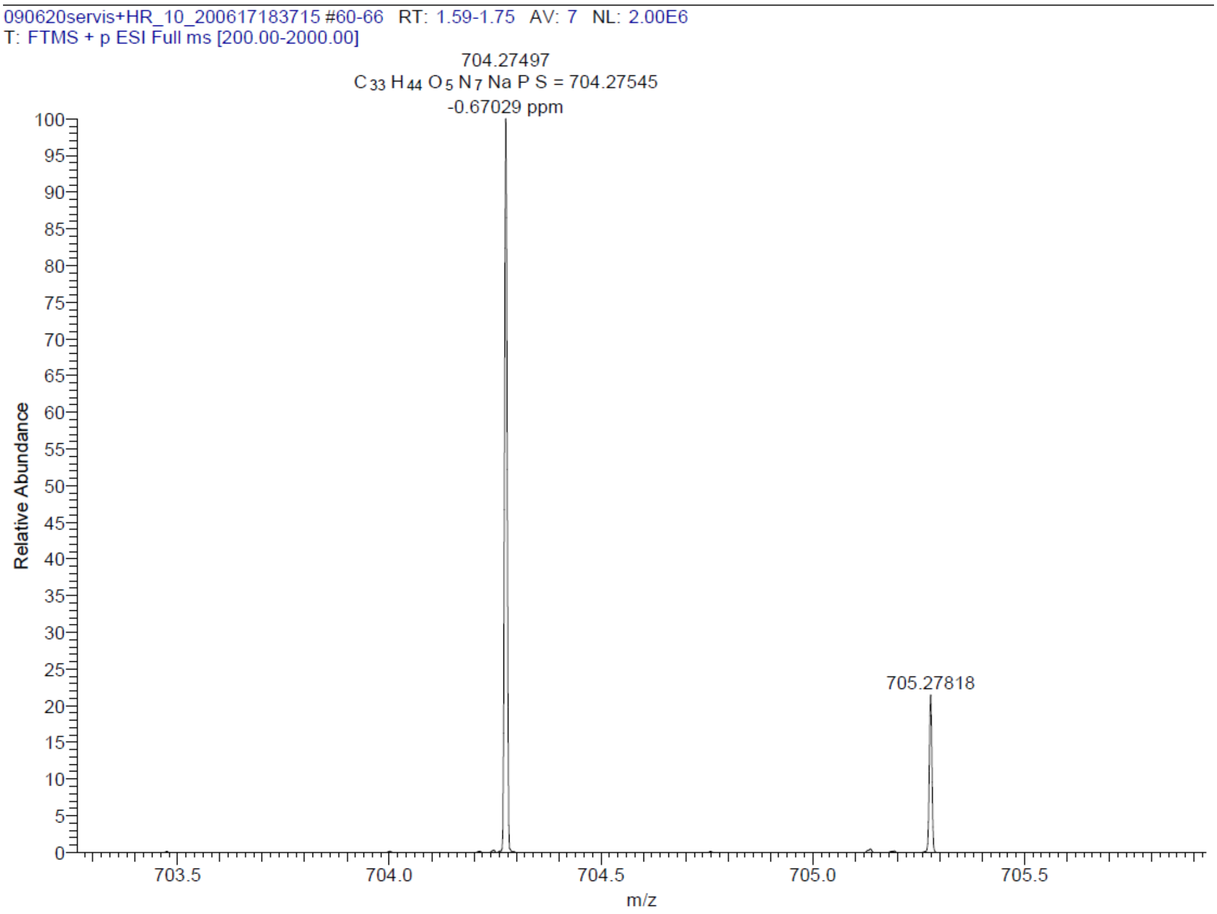

24b

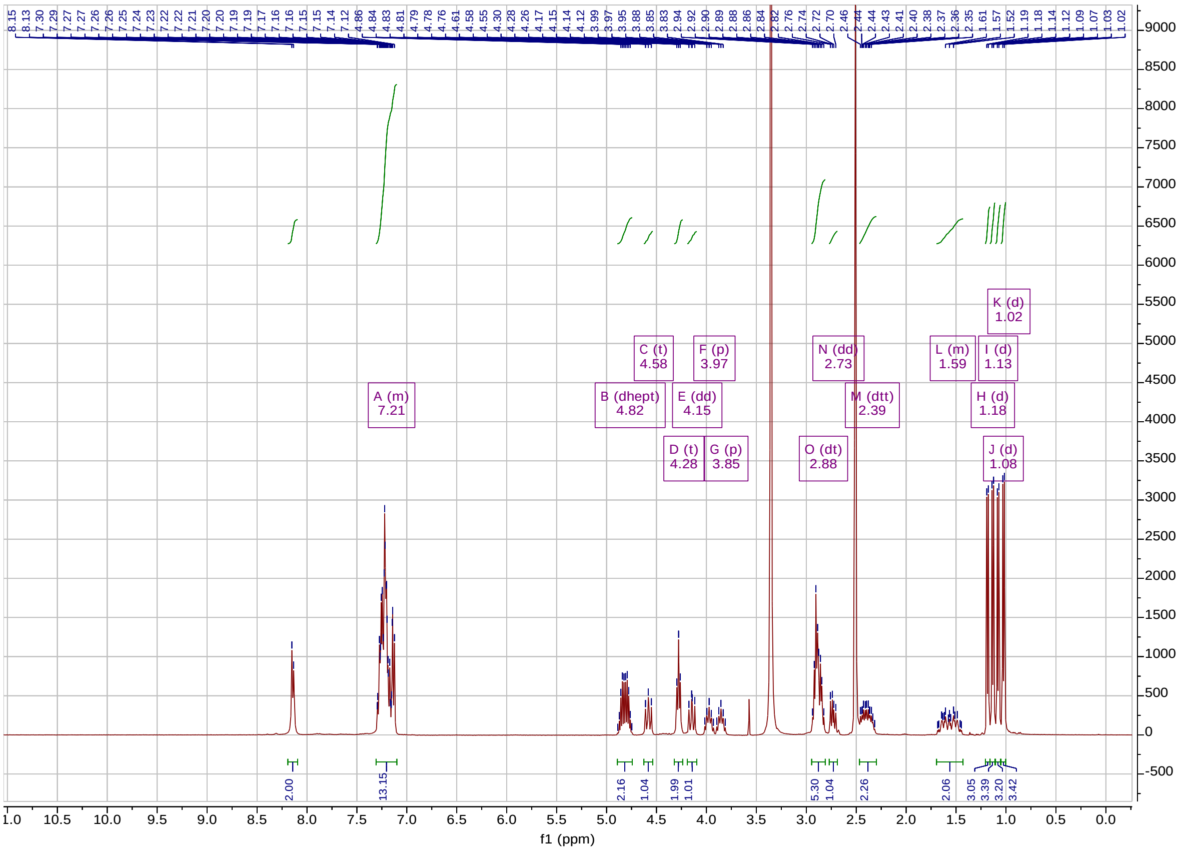
A (m) 7.21
B (dhept) 4.82
C (t) 4.58
D (t) 4.28
E (dd) 4.15
F (p) 3.97
G (p) 3.85
O (dt) 2.88
N (dd) 2.73
M (dtt) 2.39
L (m) 1.59
H (d) 1.18
I (d) 1.13
J (d) 1.08
K (d) 1.02
f1 (ppm)

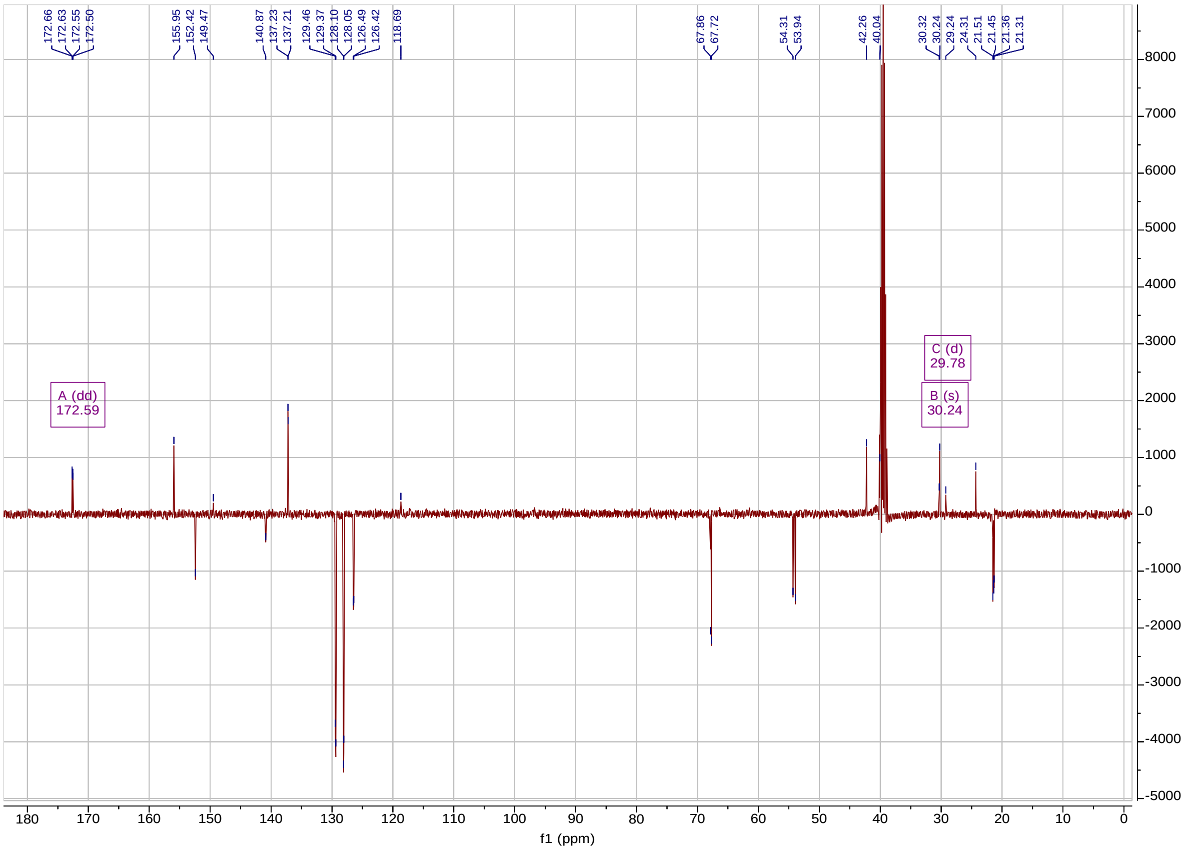
A (dd) 172.59
B (s) 30.24
C (d) 29.78
f1 (ppm)

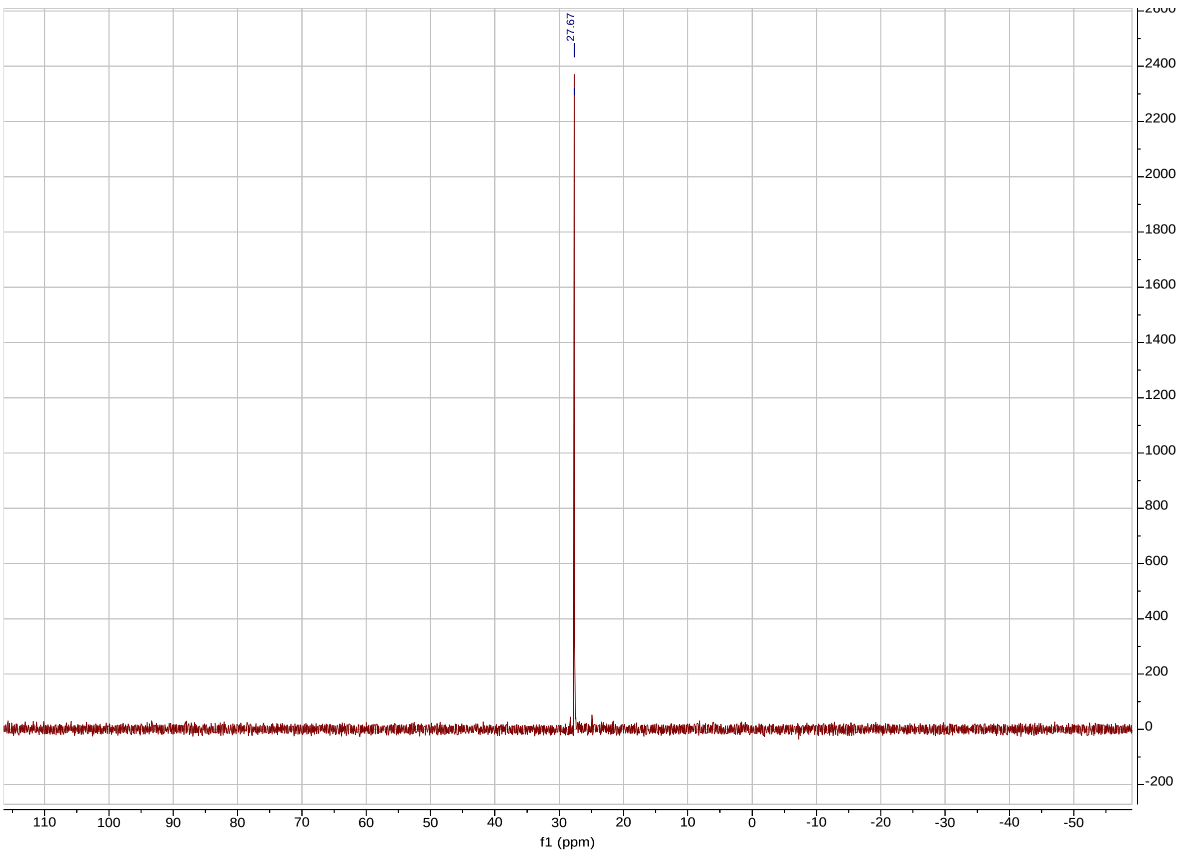
27.67
2400
2200
2000
1800
1600
1400
1200
1000
800
600
400
200
0
-200
110 100 90 80 70 60 50 40 30 20 10 0 -10 -20 -30 -40 -50
f1 (ppm)

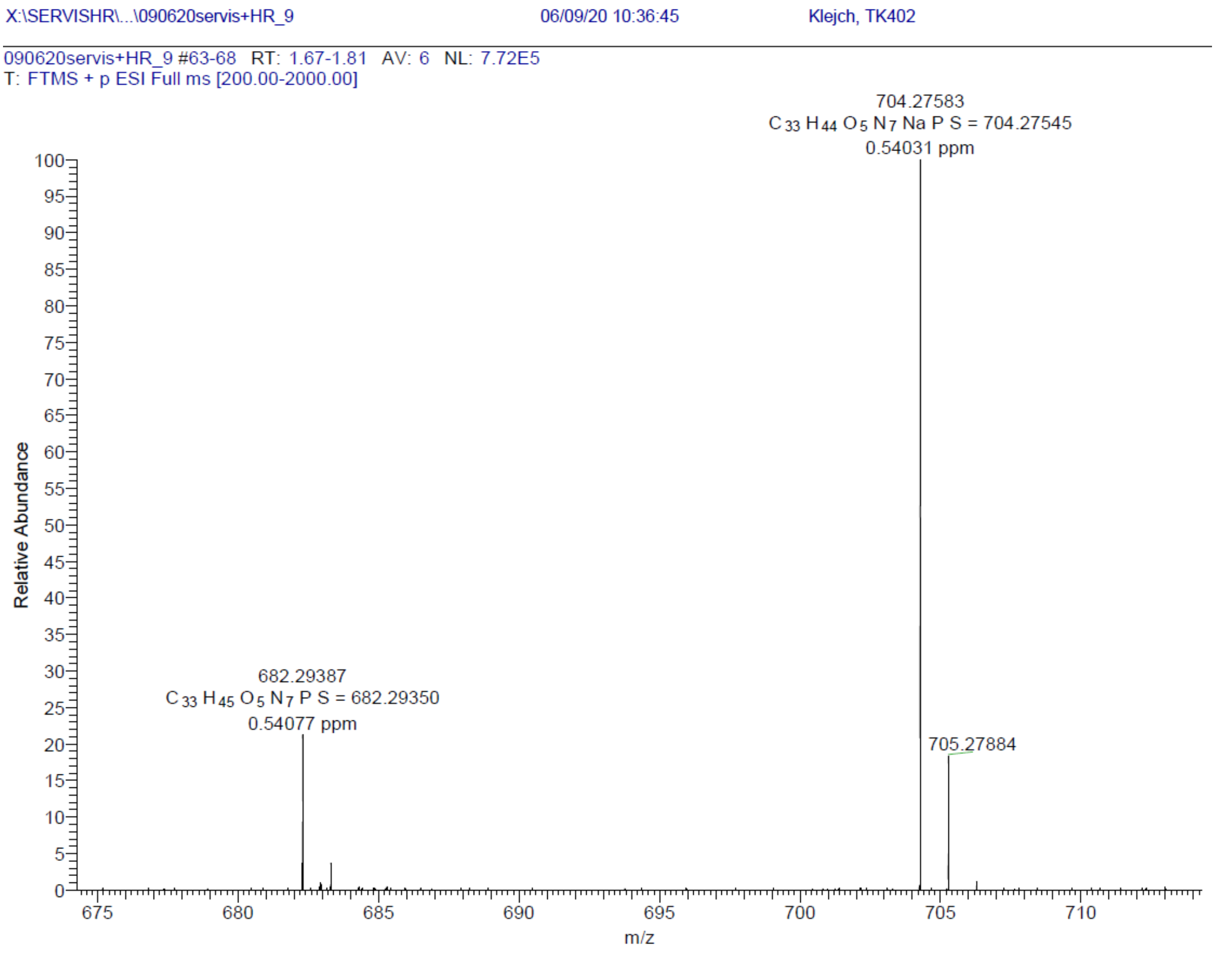
X:\SERVISHR\...\090620servis+HR_9
06/09/20 10:36:45
Klejch, TK402
090620servis+HR_9 #63-68 RT: 1.67-1.81 AV: 6 NL: 7.72E5
T: FTMS + p ESI Full ms [200.00-2000.00]
704.27583
C 33 H 44 O 5 N 7 Na P S = 704.27545
0.54031 ppm
682.29387
C 33 H 45 O 5 N 7 P S = 682.29350
0.54077 ppm
705.27884
Relative Abundance
m/z

25

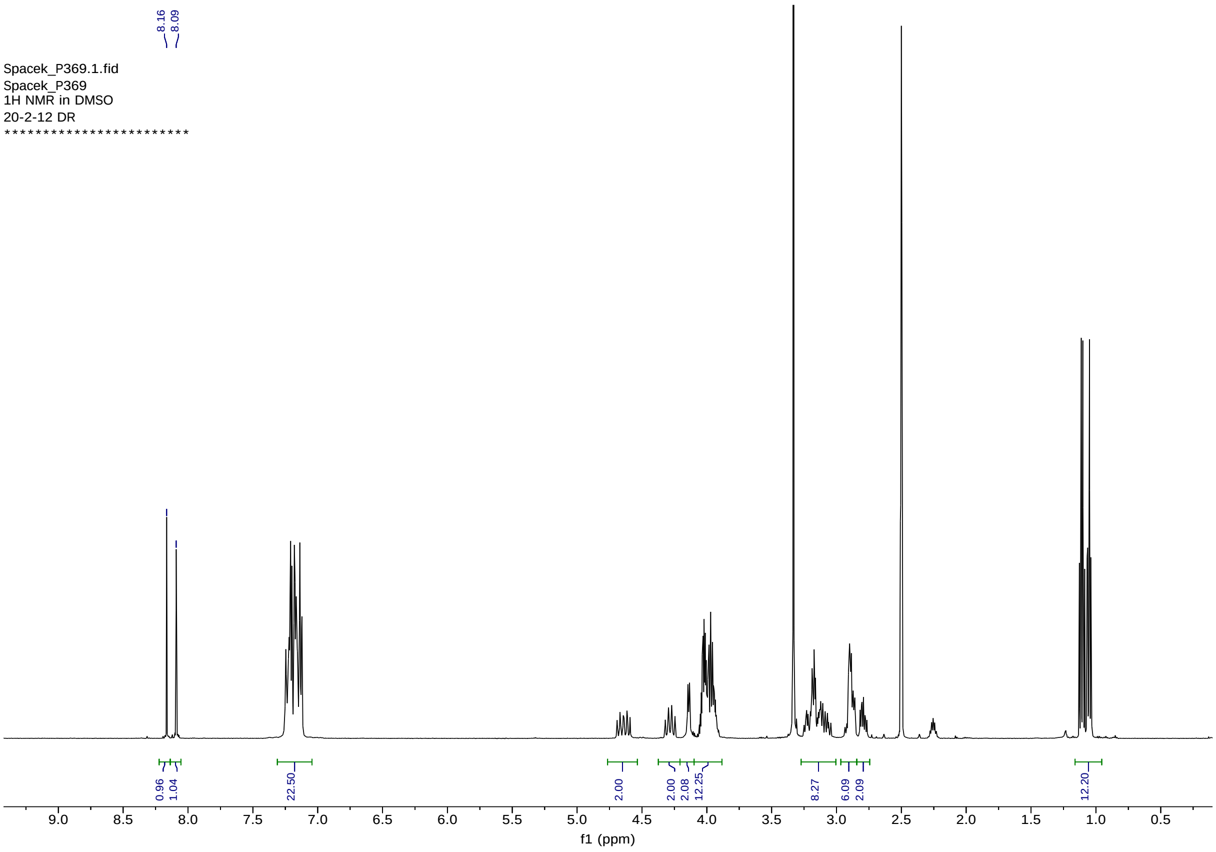
8.16
8.09
Spacek_P369.1.fid
Spacek_P369
1H NMR in DMSO
20-2-12 DR
****
0.96
1.04
22.50
2.00
2.00
2.08
12.25
8.27
6.09
2.09
12.20
9.0 8.5 8.0 7.5 7.0 6.5 6.0 5.5 5.0 4.5 4.0 3.5 3.0 2.5 2.0 1.5 1.0 0.5
f1 (ppm)

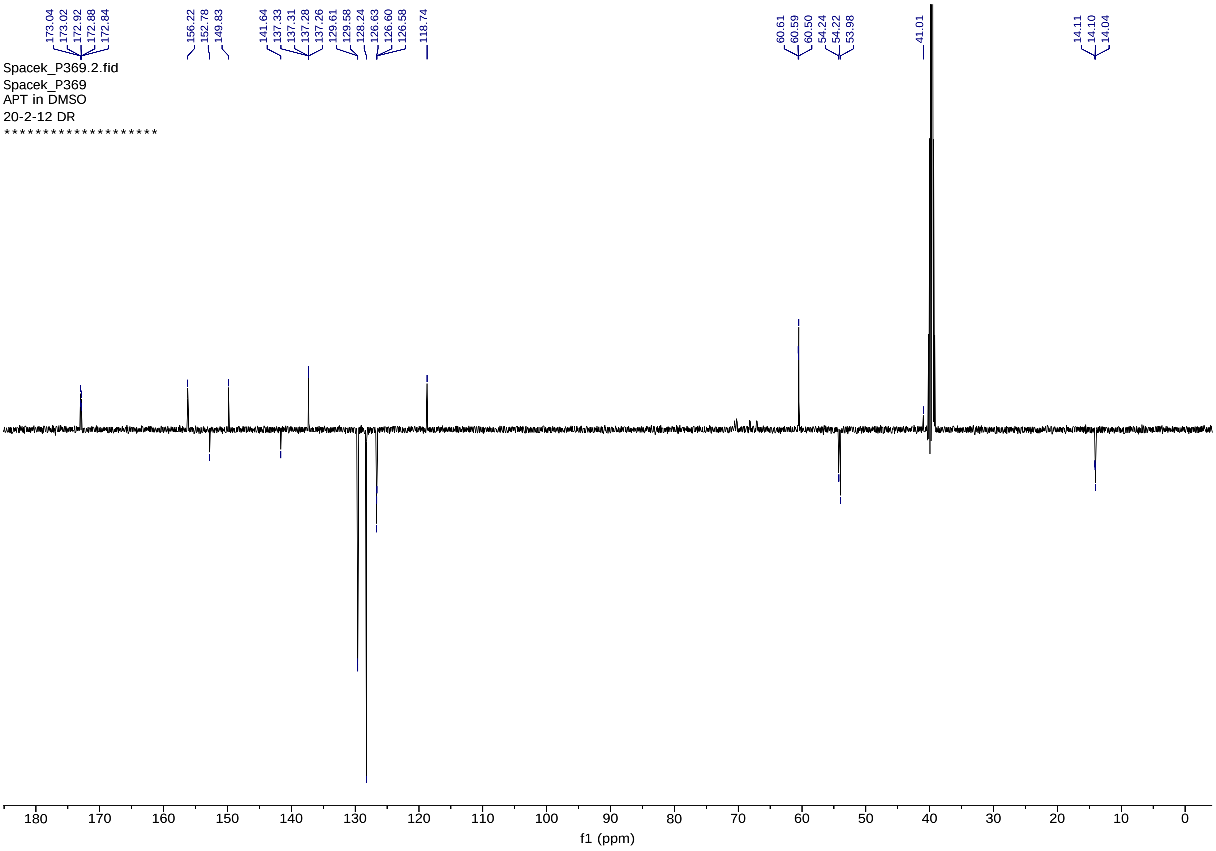
173.04
173.02
172.92
172.88
172.84
156.22
152.78
149.83
141.64
137.33
137.31
137.28
137.26
129.61
129.58
128.24
126.63
126.60
126.58
118.74
60.61
60.59
60.50
54.24
54.22
53.98
41.01
14.11
14.10
14.04
Spacek_P369.2.fid
Spacek_P369
APT in DMSO
20-2-12 DR
****
180 170 160 150 140 130 120 110 100 90 80 70 60 50 40 30 20 10 0
f1 (ppm)

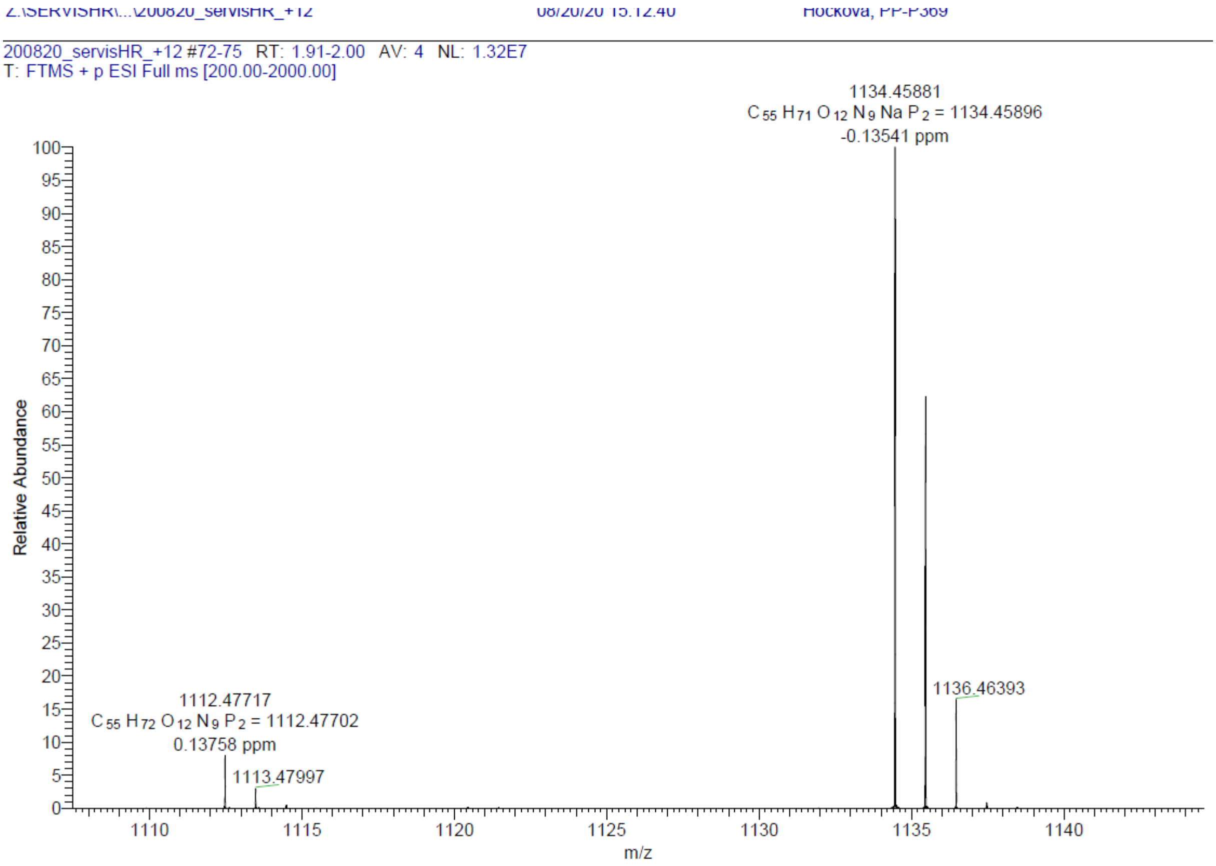
Z:\SERVISHR\...\200820_servisHR_+12
08/20/20 15:12:40
Hockova, PP-P369
200820_servisHR_+12 #72-75 RT: 1.91-2.00 AV: 4 NL: 1.32E7
T: FTMS + p ESI Full ms [200.00-2000.00]
1134.45881
C 55 H 71 O 12 N 9 Na P 2 = 1134.45896
-0.13541 ppm
1136.46393
1112.47717
C 55 H 72 O 12 N 9 P 2 = 1112.47702
0.13758 ppm
1113.47997
Relative Abundance
m/z

26

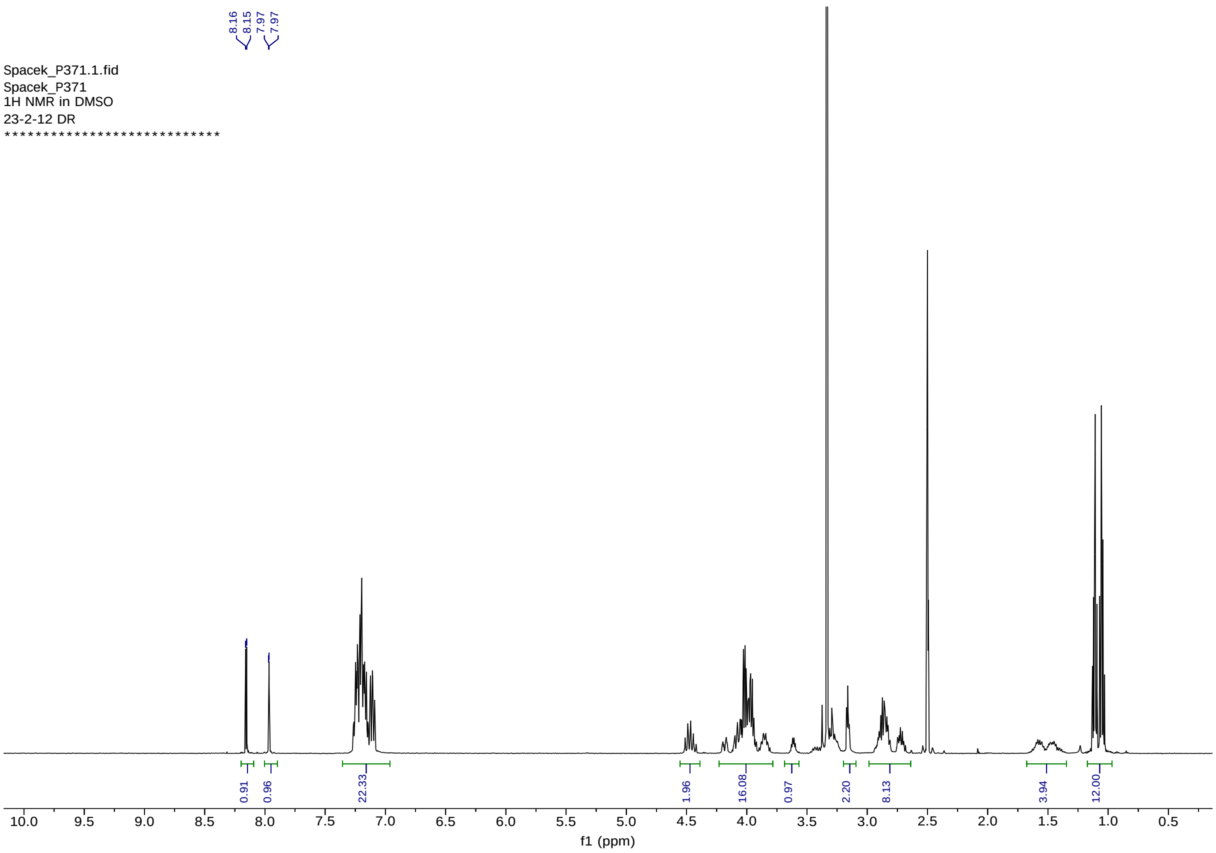
Spacek_P371.1.fid
Spacek_P371
1H NMR in DMSO
23-2-12 DR
****
8.16
8.15
7.97
7.97
0.91
0.96
22.33
1.96
16.08
0.97
2.20
8.13
3.94
12.00
f1 (ppm)

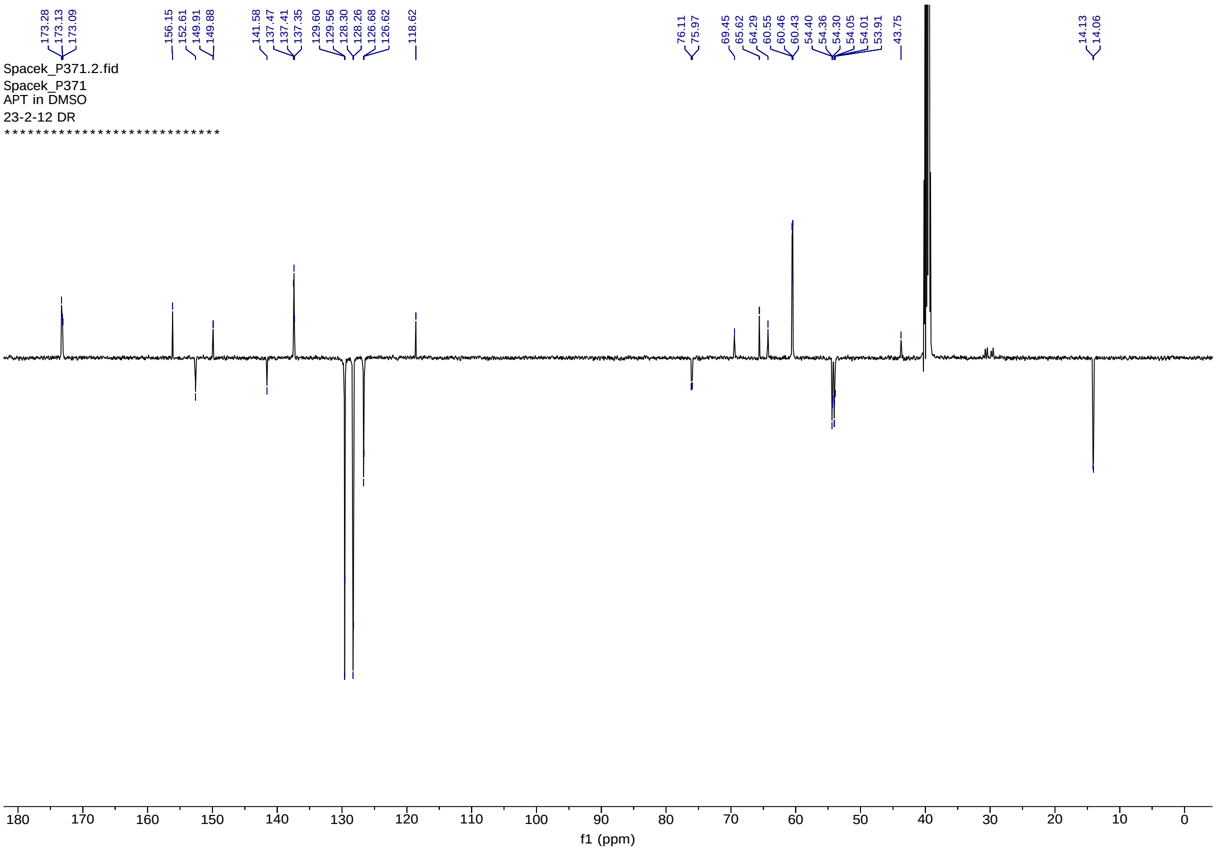

Z:\SERVISHR\...\200820_servisHR_+13 08/20/20 15:16:44 Hockova, PP-P371

200820_servisHR_+13 #80-83 RT: 2.13-2.21 AV: 4 NL: 4.45E5
T: FTMS + p ESI Full ms [200.00-2000.00]

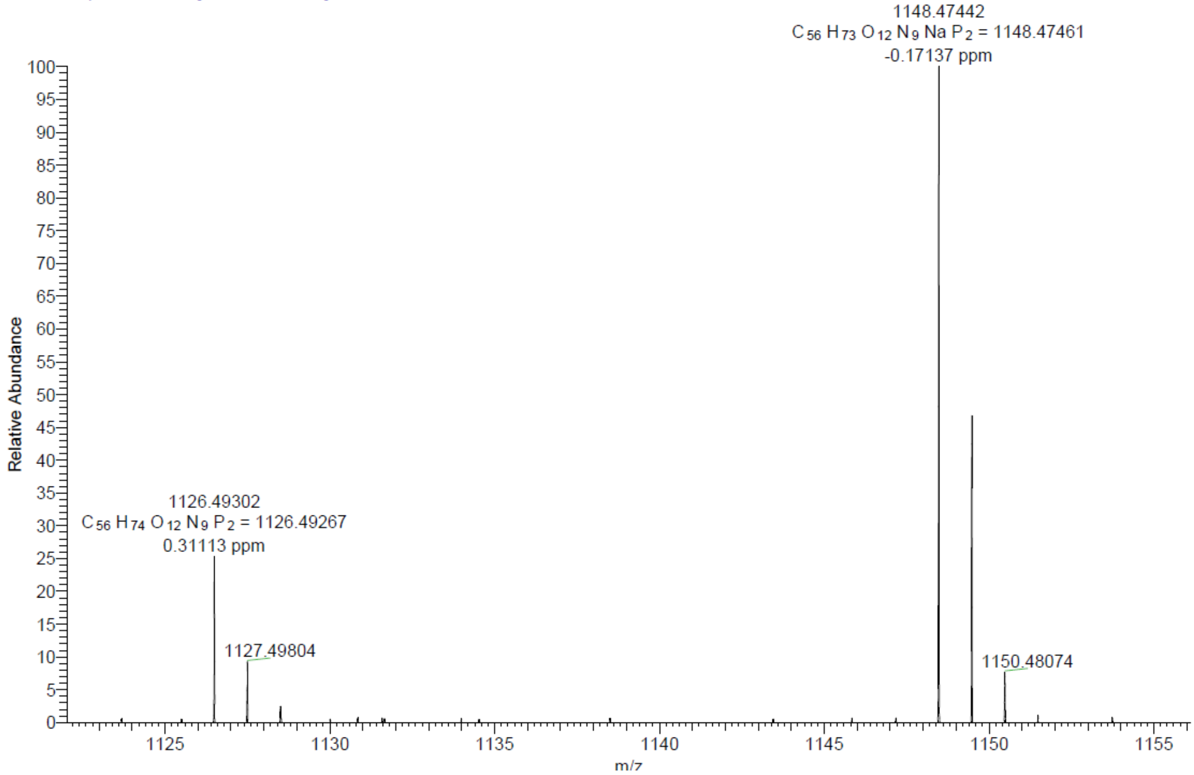

27

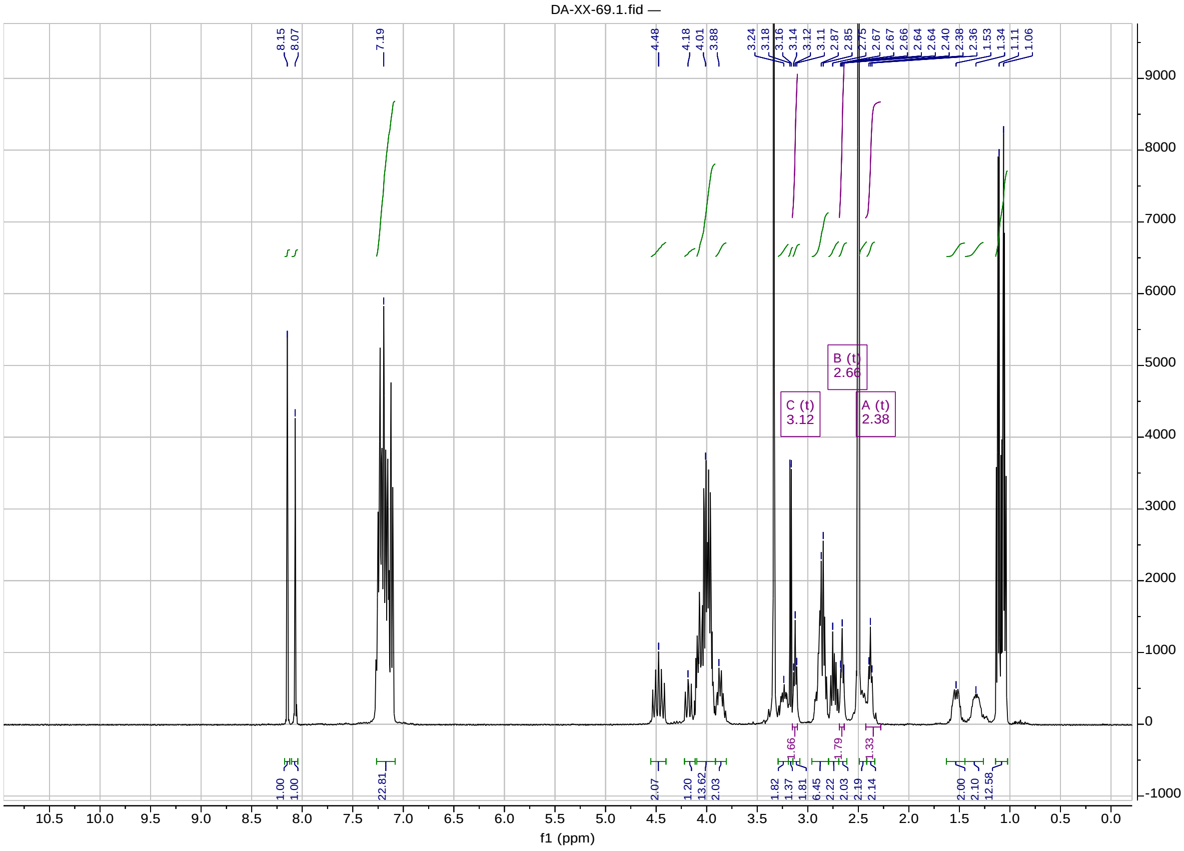

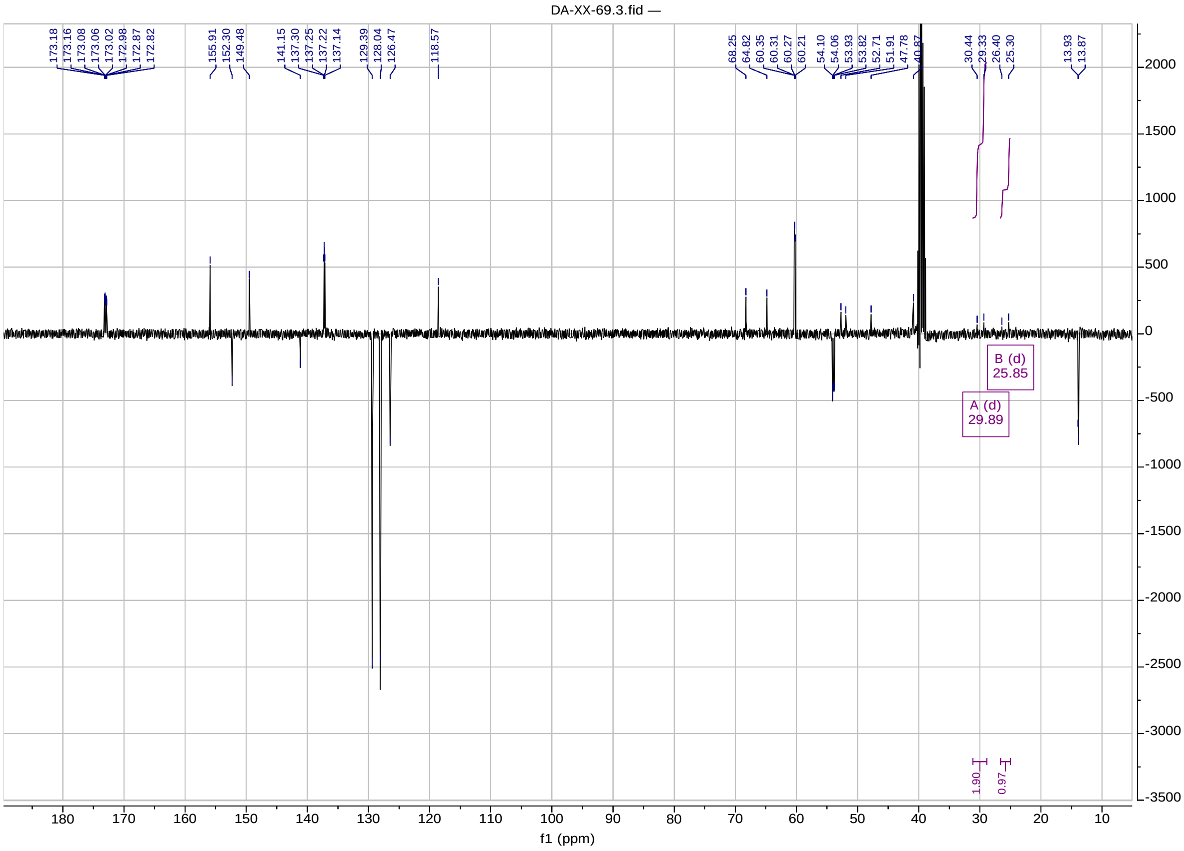

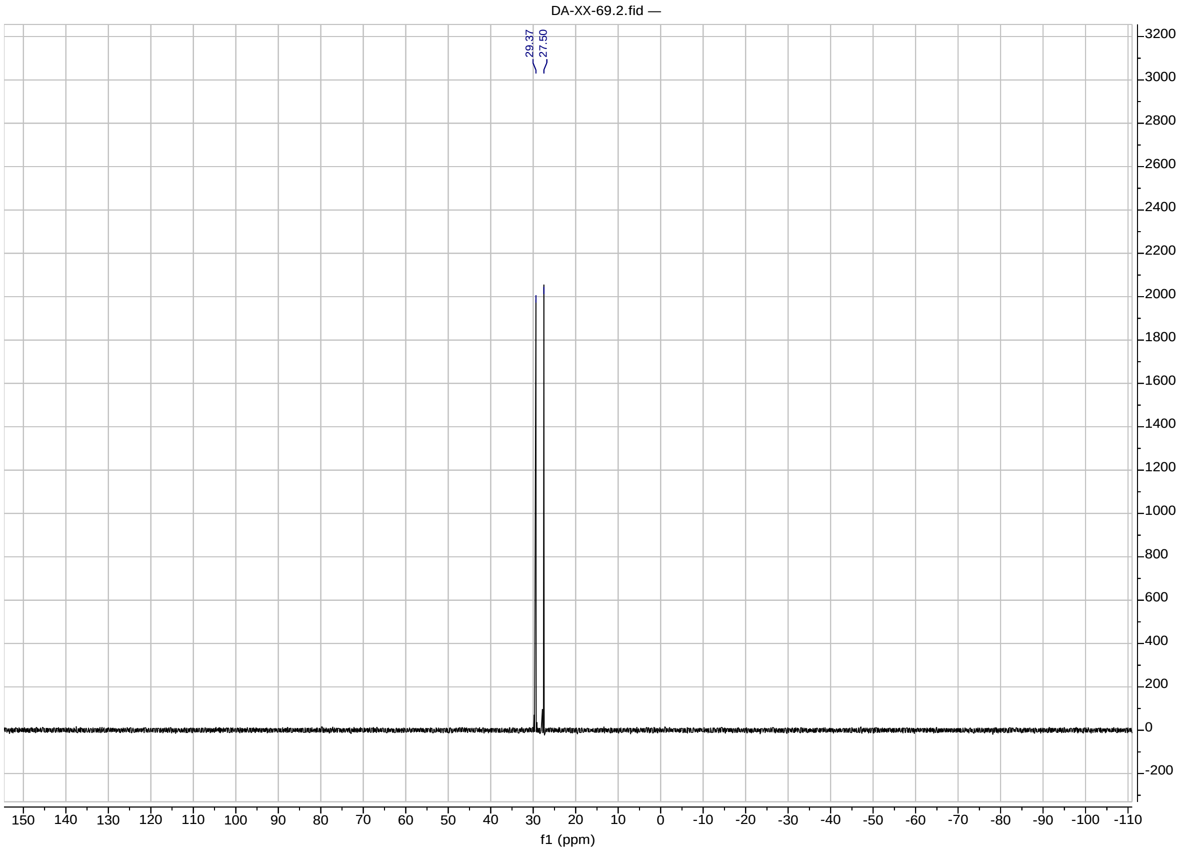
DA-XX-69.2.fid —
29.37
27.50
f1 (ppm)

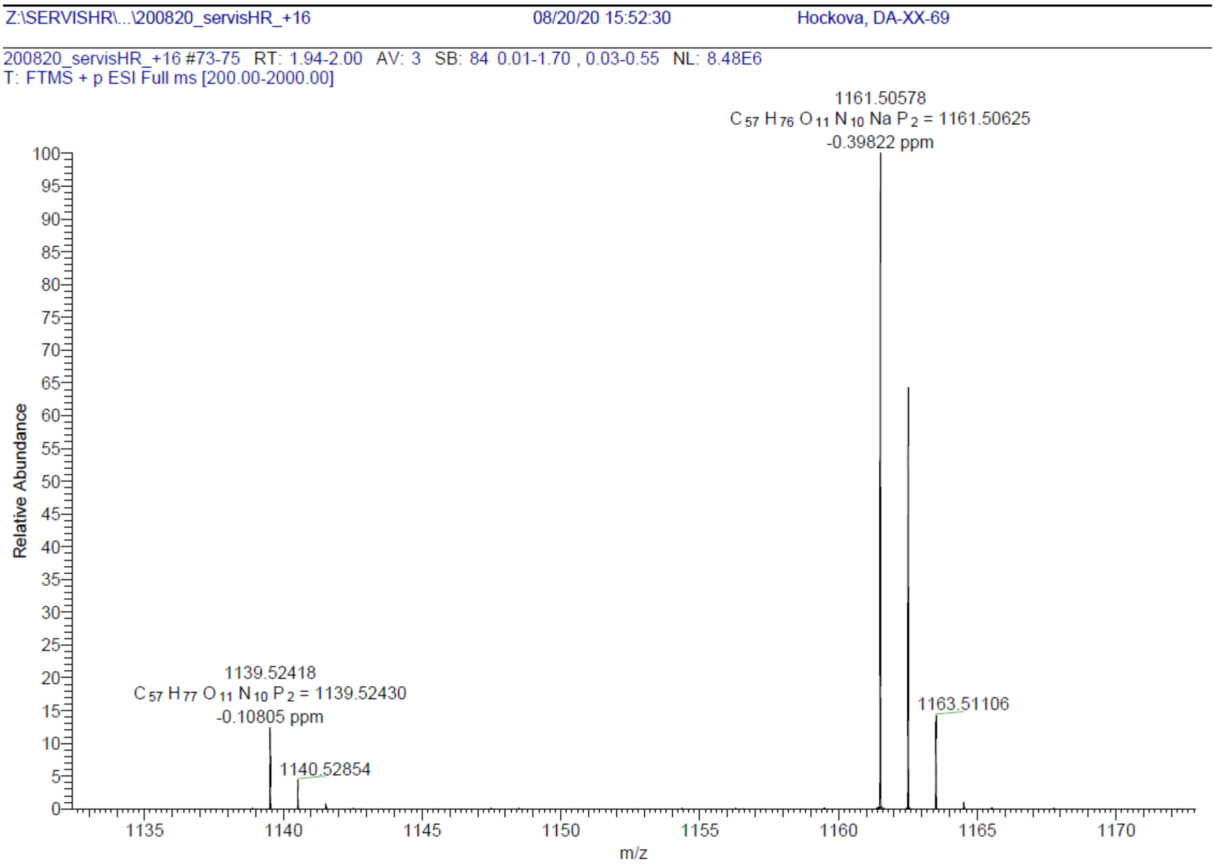
Z:\SERVISHR\...\200820_servisHR_+16
08/20/20 15:52:30
Hockova, DA-XX-69
200820_servisHR_+16 #73-75 RT: 1.94-2.00 AV: 3 SB: 84 0.01-1.70 , 0.03-0.55 NL: 8.48E6
T: FTMS + p ESI Full ms [200.00-2000.00]
1161.50578
C 57 H 76 O 11 N 10 Na P 2 = 1161.50625
-0.39822 ppm
1139.52418
C 57 H 77 O 11 N 10 P 2 = 1139.52430
-0.10805 ppm
1140.52854
1163.51106
Relative Abundance
m/z

28

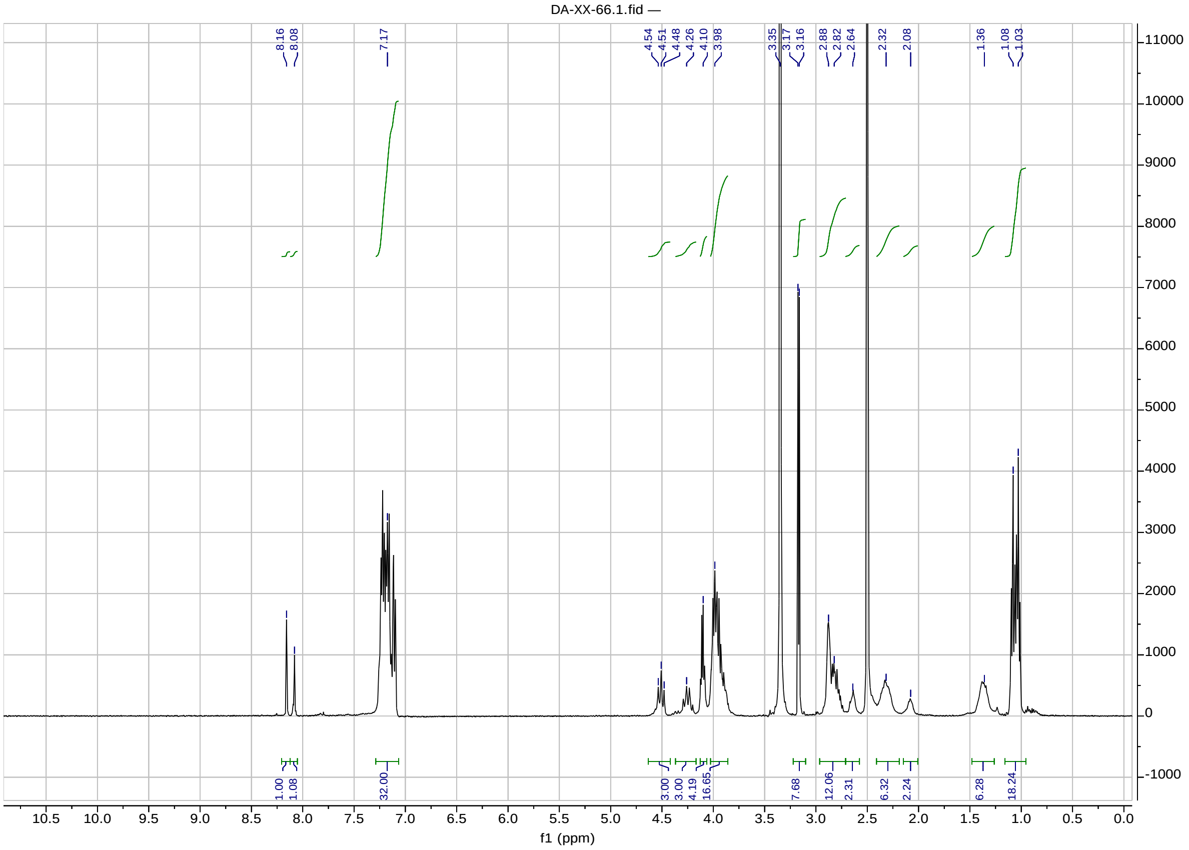

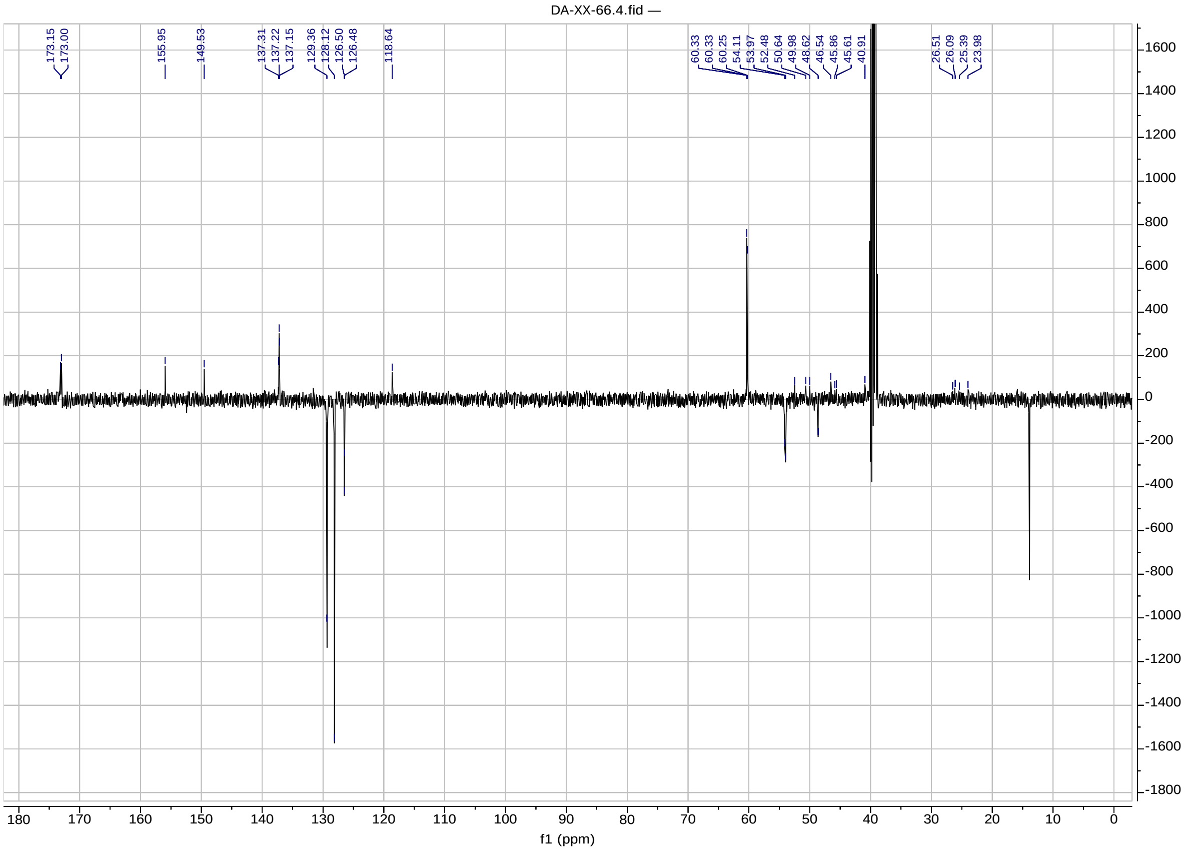

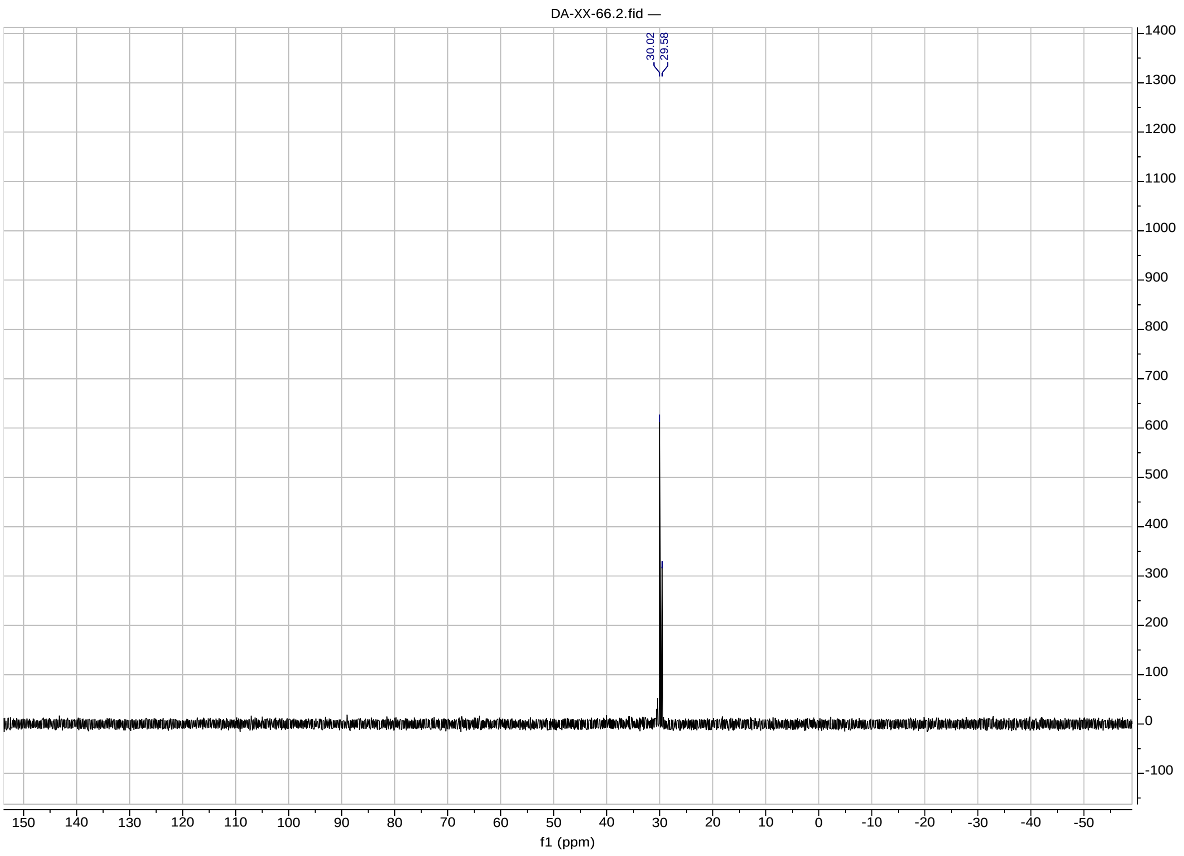
DA-XX-66.2.fid —
30.02
29.58
1400
1300
1200
1100
1000
900
800
700
600
500
400
300
200
100
0
-100
150 140 130 120 110 100 90 80 70 60 50 40 30 20 10 0 -10 -20 -30 -40 -50
f1 (ppm)

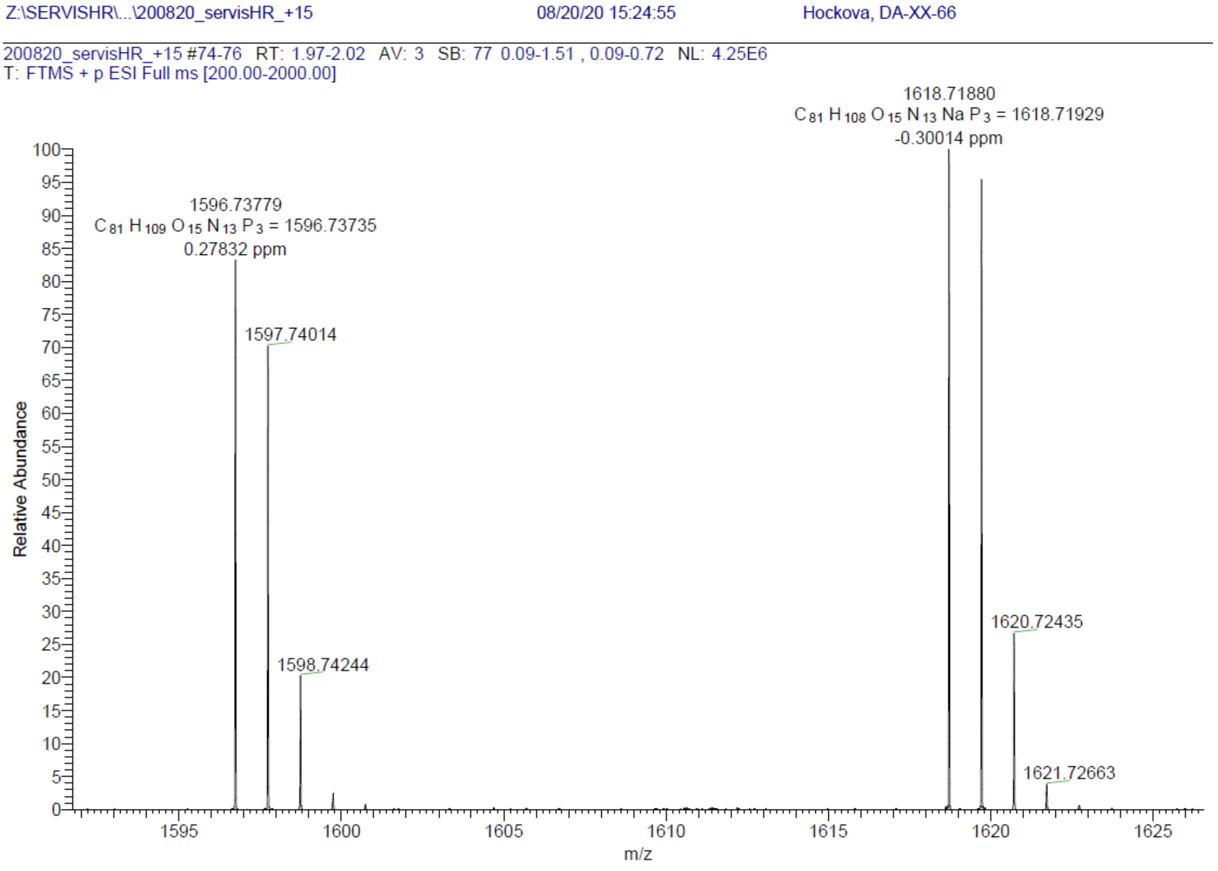
Z:\SERVISHR\...\200820_servisHR_+15
08/20/20 15:24:55
Hockova, DA-XX-66
200820_servisHR_+15 #74-76 RT: 1.97-2.02 AV: 3 SB: 77 0.09-1.51 , 0.09-0.72 NL: 4.25E6
T: FTMS + p ESI Full ms [200.00-2000.00]
1618.71880
C 81 H 108 O 15 N 13 Na P 3 = 1618.71929
-0.30014 ppm
1596.73779
C 81 H 109 O 15 N 13 P 3 = 1596.73735
0.27832 ppm
1597.74014
1598.74244
1620.72435
1621.72663
Relative Abundance
100
95
90
85
80
75
70
65
60
55
50
45
40
35
30
25
20
15
10
5
0
1595 1600 1605 1610 1615 1620 1625
m/z

Supplementary file S3. Raw Images. Original images supporting blot results reported in Figs. 2A, 3 and 4C

S1_raw images

Fig. 2A

v5-tagged APRT1

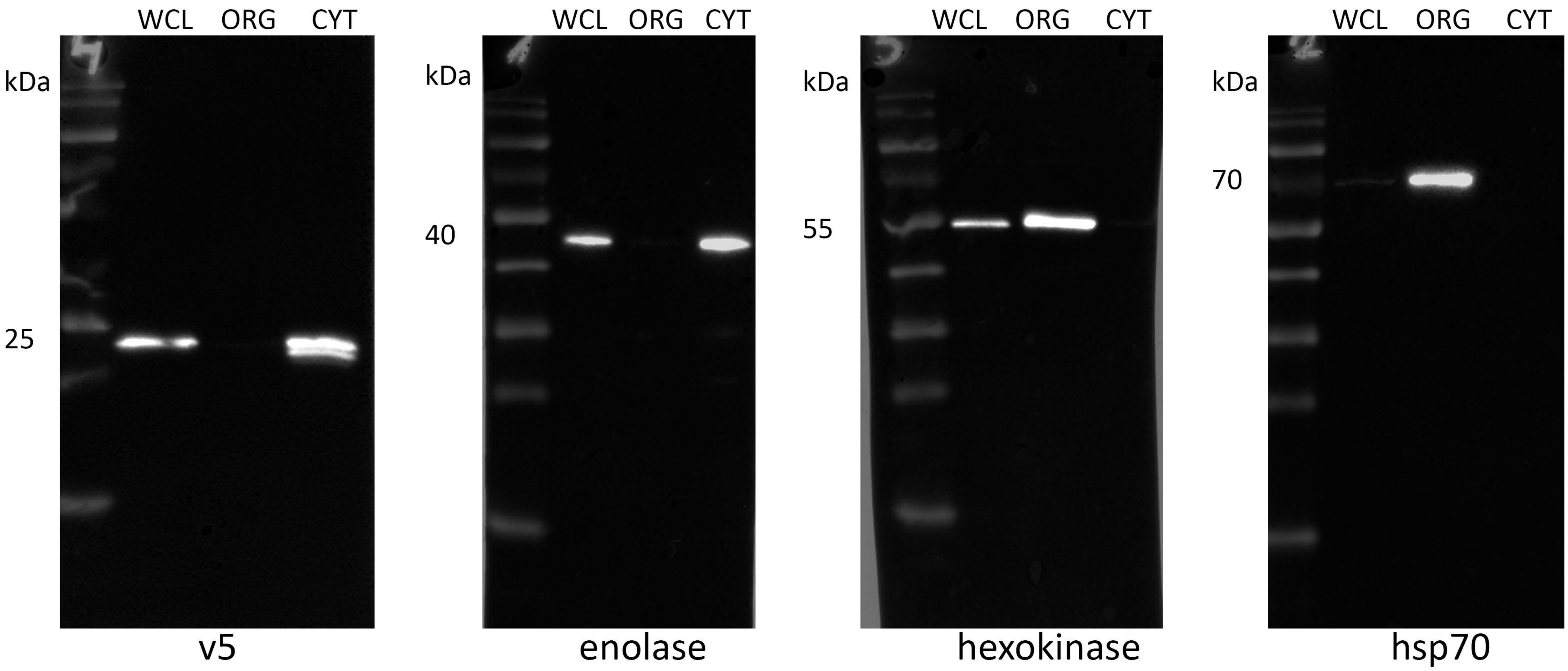

v5-tagged APRT2

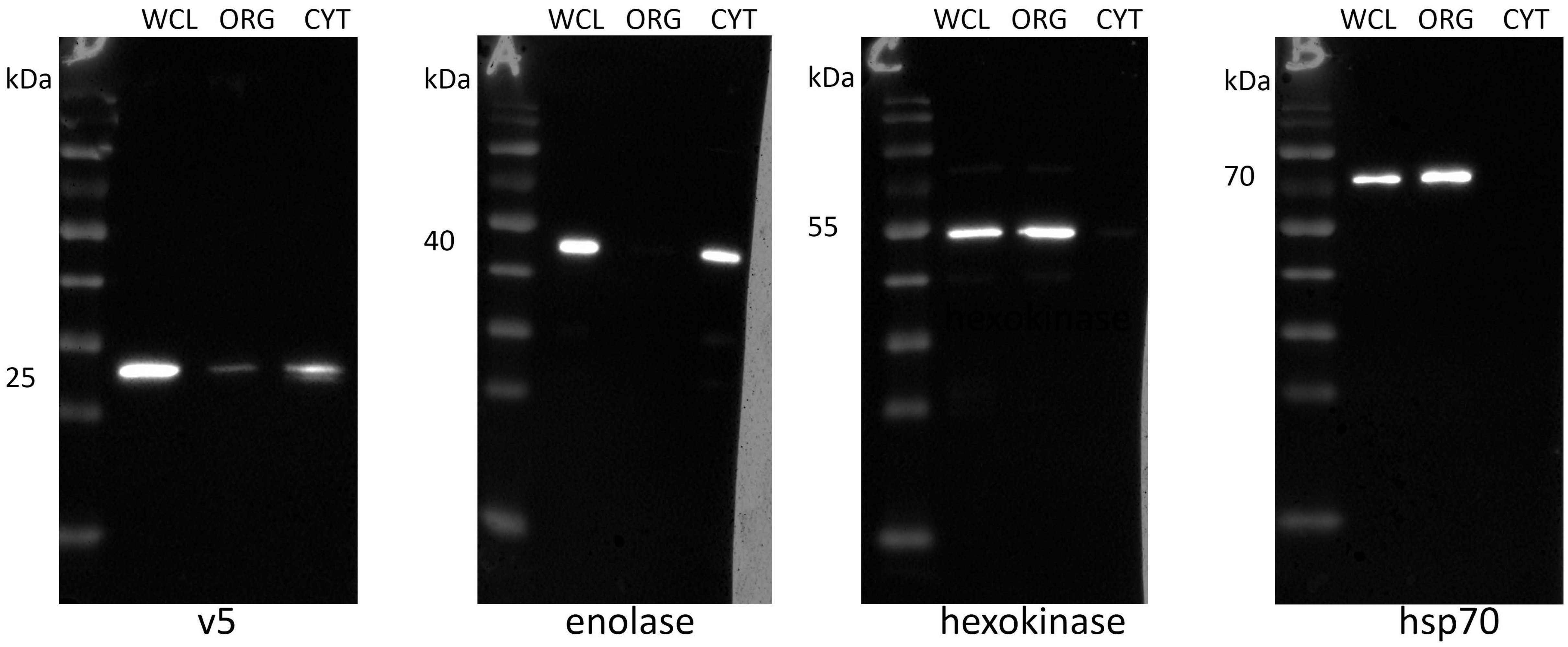

Fig. 3

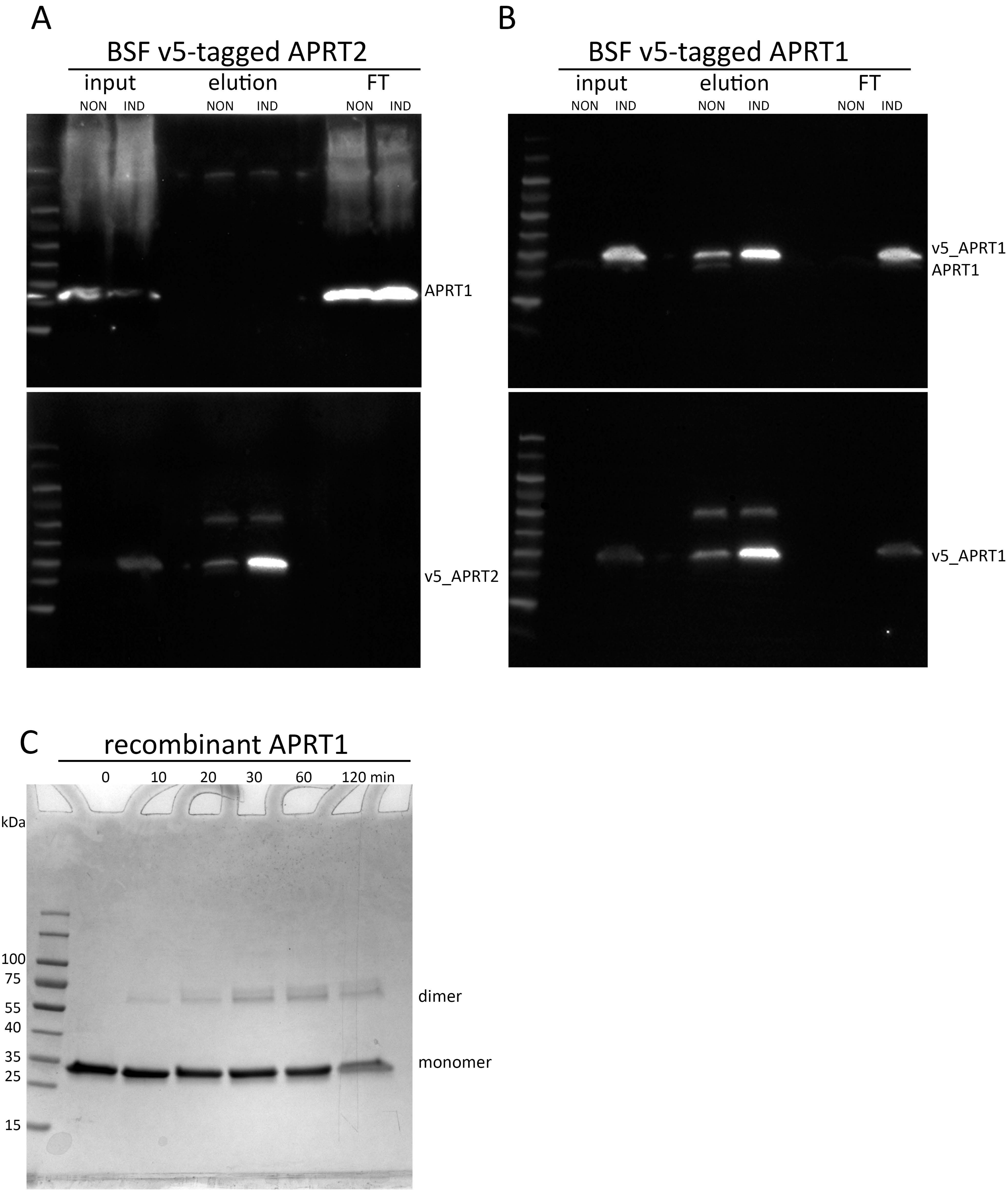
A
BSF v5-tagged APRT2
input
elution
FT
NON
IND
NON
IND
NON
IND
APRT1
v5_APRT2
B
BSF v5-tagged APRT1
input
elution
FT
NON
IND
NON
IND
NON
IND
v5_APRT1
APRT1
v5_APRT1
C
recombinant APRT1
0
10
20
30
60
120 min
kDa
100
75
55
40
35
25
15
dimer
monomer

Fig. 4B

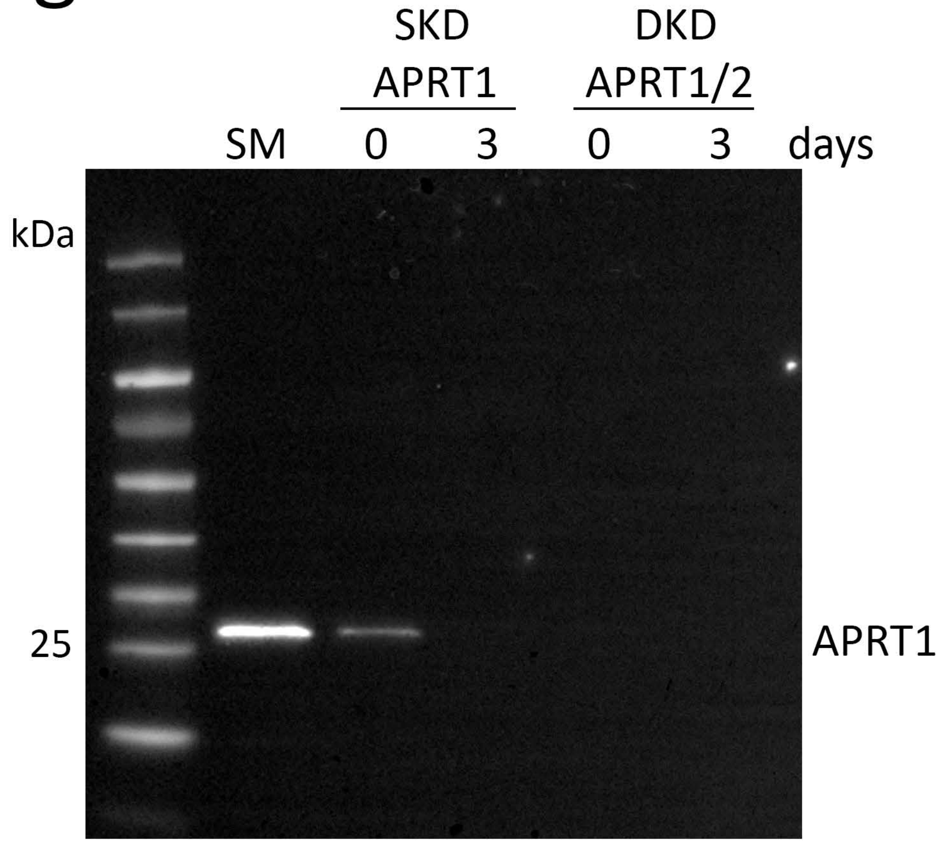
SKD
APRT1
DKD
APRT1/2
SM
0
3
0
3
days
kDa
25
APRT1
SKD
APRT1
DKD
APRT1/2
SM
0
3
0
3
days
kDa
70
hsp70
